# The influence of transcript assembly on the proteogenomics discovery of microproteins

**DOI:** 10.1371/journal.pone.0194518

**Published:** 2018-03-27

**Authors:** Jiao Ma, Alan Saghatelian, Maxim Nikolaievich Shokhirev

**Affiliations:** 1 Clayton Foundation Laboratories for Peptide Biology, Salk Institute for Biological Studies, La Jolla, CA, United States of America; 2 Razavi Newman Integrative Genomics and Bioinformatics Core, Salk Institute for Biological Studies, La Jolla, CA, United States of America; Huazhong University of Science and Technology, CHINA

## Abstract

Proteogenomics methods have identified many non-annotated protein-coding genes in the human genome. Many of the newly discovered protein-coding genes encode peptides and small proteins, referred to collectively as microproteins. Microproteins are produced through ribosome translation of small open reading frames (smORFs). The discovery of many smORFs reveals a blind spot in traditional gene-finding algorithms for these genes. Biological studies have found roles for microproteins in cell biology and physiology, and the potential that there exists additional bioactive microproteins drives the interest in detection and discovery of these molecules. A key step in any proteogenomics workflow is the assembly of RNA-Seq data into likely mRNA transcripts that are then used to create a searchable protein database. Here we demonstrate that specific features of the assembled transcriptome impact microprotein detection by shotgun proteomics. By tailoring transcript assembly for downstream mass spectrometry searching, we show that we can detect more than double the number of high-quality microprotein candidates and introduce a novel open-source **m**RNA **a**ssembler for **p**roteogenomic**s** (MAPS) that incorporates all of these features. By integrating our specialized assembler, MAPS, and a popular generalized assembler into our proteogenomics pipeline, we detect 45 novel human microproteins from a high quality proteogenomics dataset of a human cell line. We then characterize the features of the novel microproteins, identifying two classes of microproteins. Our work highlights the importance of specialized transcriptome assembly upstream of proteomics validation when searching for short and potentially rare and poorly conserved proteins.

## Introduction

Genetic studies in flies led to the discovery of an 11-amino acid peptide that control embryo and limb development named tarsalless (tal) or polished rice (pri) [1–4]. Unlike classical peptide hormones, such as insulin or glucagon, that are produced from the proteolysis of a longer prohormones, tal/pri comes from ribosomal translation of a small 33-nucleotide open reading frame (ORF). The conserved tal/pri peptide demonstrates direct ribosomal production of peptides and small proteins, or microproteins, with fundamental biological activities from small ORFs (smORFs).

The discovery of tal/pri led triggered a rush to systematically discover non-annotated smORFs and microproteins in different organisms. The removal of a length cutoff for gene-finding algorithms led to the prediction of thousands of additional smORFs [5], provided early evidence in support of smORF discovery projects. Ribosome sequencing (Ribo-Seq)—a ribosome footprinting technique that measures the position of the ribosome on RNA that is used to infer translated sequences—and proteogenomics revealed the greatest number of smORFs. Using these methods, hundreds to thousands of smORFs have been discovered in flies [1, 6], mice [7], and humans [8–11]. In aggregate, the discovery of so many non-annotated smORFs revealed a blind spot in gene finding algorithms for protein-coding genes less than 100 codons. Furthermore, some of these newly discovered smORFs have been characterized and shown to have cellular and physiological functions, supporting the idea that smORFs produce many functional microproteins [12–16].

**Fig 1:**
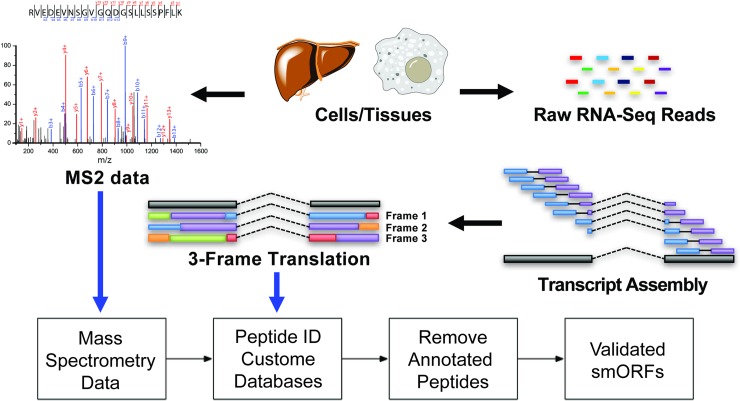
Proteogenomics microprotein discovery pipeline. High quality RNA-Seq data is assembled and three-frame translated to create an *in silico* custom proteomics database. The database is then used to search MS2 spectra to obtain peptide candidates. Short un-annotated peptides with a high quality MS2 spectra are manually curated to produce a list of novel confident microproteins. Since peptide discovery depends on the assembled transcriptome, we propose to optimize transcriptome assembly for peptide discovery.

Proteogenomics is unique among microprotein discovery strategies because this approach provides direct evidence for the microprotein translation through proteomics detection of the resultant peptide. Proteogenomics refers to methods that combine mRNA sequencing (RNA-Seq) and proteomics to the detection non-annotated protein expression. For example, application of proteogenomics to microprotein discovery led to the identification of 365 microproteins in several human cell lines and tissues [8, 9, 17] (Fig 1).

The key computational step for successful microprotein discovery is the creation of a protein database from RNA-Seq data [8, 17]. After collecting high-quality paired end RNA-Seq data, which consists of several hundred million pairs of 100–150 nucleotide sequences from each end of a transcript fragment, the data must be assembled into mRNA transcripts. Transcript assembly begins by alignment of RNA reads onto a reference genome using a splice aware aligner such as STAR [18]. The output from the aligner is converted into mRNAs by transcript assemblers such as Cufflinks [19]. Three frame translation of these mRNAs *in silico* generate a custom proteomics database [20, 21], which is used to build a collection of reference spectra that can be matched to recorded spectra from mass spectrometry runs [9, 17]. Because of the central role of mRNA assembly in this process, we set out to understand and optimize transcript assembly for microprotein detection.

We test the influence of RNA-Seq transcriptome assembly on the proteogenomics discovery of microproteins by developing our own assembler, **m**RNA **A**ssembler for **P**roteogenomic**S** (MAPS). MAPS is a specialized transcript assembler which allows us to optimize for both transcript accuracy and transcriptome diversity, an important factor for downstream proteomics spectral searching. In addition, MAPS extends open reading frames up and downstream to the nearest stop codon, and uses the aligned read sequences to account for cell-line specific mutations to improve the accuracy of open reading frames. Working with simulated realistic RNA-Seq data and published high-quality mouse ENCODE RNA-Seq data, we show that MAPS can improve the diversity of the assembled transcriptome, allowing for a larger recall at the cost of a lower precision. Integration of MAPS into a proteogenomics pipeline led to the discovery of 23 novel microproteins (S1 Table).

## Materials and methods

### HEK293T library preparation and sequencing

Total RNA was isolated using Pure-Link RNA Mini Kit (Life Technologies) according to the manufacturer’s protocol. On-column digestion of genomic DNA was performed using DNase I (NEB). The Agilent Bioanalyzer or Tape Station was used to determine RNA integrity numbers (RIN) prior to library preparation. Total RNA (1ug) was prepared to generate Illumina-compatible sequencing libraries using three different methods: 1) Illumina Stranded mRNA-Seq, 2) Illumina Total RNA-Seq, and 3) KAPA Total RNA-Seq.

Stranded mRNA-Seq libraries were prepared using Illumina TruSeq Stranded mRNA Library Prep Kit according to the manufacturer’s instructions. Briefly, poly-A RNA was isolated using magnetic beads conjugated to poly-T oligonucleotides. The mRNA was then fragmented and reverse-transcribed into cDNA. dUTPs were incorporated, followed by second strand cDNA synthesis. dUTP-incorporated second strand was not amplified. The cDNA library was then end-repaired, index adapter-ligated and PCR amplified. AMPure XP beads (Beckman Coulter) were used to purify nucleic acid after each steps of the library prep.

Total Stranded mRNA-Seq libraries were prepared using the Illumina TruSeq Stranded Total RNA Sample Prep with Ribo-Zero Gold Kit following manufacturer’s instructions. First, cytoplasmic and mitochondrial ribosomal RNA (rRNA) was depleted using biotinylated oligo probes complementary to ribosomal transcripts. Depleted RNA was then prepared into stranded RNA-Seq libraries using same protocol as mentioned above starting at the RNA fragmentation steps.

KAPA Total RNA-Seq libraries were prepared using the KAPA Stranded RNA-Seq Kit with Ribo-erase (KAPA Biosystems). Protocol is like the total RNA-Seq libraries, except prior to RNA fragmentation, rRNA was hybridized to DNA oligos, then treated with RNase H and DNase to remove RNA-DNA duplexes, as well as original DNA oligos. All sequencing libraries were then quantified, pooled and sequenced at paired-end 150 base-pair using the Illumina NextSeq 500 at the Salk NGS Core. Raw sequencing data was demultiplexed and converted into FASTQ files using CASAVA (v1.8.2). Libraries were sequenced at an average depth of 519 million paired reads per library.

### Sequenced read alignment

Sequenced reads were quality-tested using FASTQC (http://www.bioinformatics.babraham.ac.uk/projects/fastqc) and aligned to the hg19[22] human genome using the STAR aligner[18] version 2.4.0k. Mapping was carried out using default parameters (up to 10 mismatches per read, and up to 9 multi-mapping locations per read). The genome index was constructed using the gene annotation supplied with the hg19 Illumina iGenomes collection (http://support.illumina.com/sequencing/sequencing_software/igenome.html) and sjdbOverhang value of 100. Reads mapping to more than one location in the genome were removed prior to transcript assembly. Reads from each method were combined prior to alignment to improve transcript diversity. Published paired RNA-Seq mouse datasets were obtained from the Encyclopedia of DNA elements[23]: ENCSR554PHF, ENCSR164BAZ, ENCSR394YLM, ENCSR518GDK, ENCSR870AQU, ENCSR216KLZ, ENCSR248XKS, ENCSR170SVO, aligned to the mm10 [24] reference genome as described above.

### Transcriptome assembly and database generation

Aligned reads were assembled into transcripts by Cufflinks[19] using default parameters, fragment bias correction, multi-read correction, fr-firststrand library construction, and the hg19 human genome (or mouse mm10 genome) sequence as a guide, or assembled using our new assembler, MAPS, which is described below. MAPS assembly consists of four parts: 1) loading and sorting reads into memory, 2) assembling reads into exons and junctions, 3) assembling exons and junctions into transcripts, 4) outputting transcripts and 3-frame translated peptide sequences for downstream proteomics search. Steps 1 and 2 are straightforward except for two important points: a) read sequences are used to build a consensus sequence database, which is used to improve the accuracy of generated open reading frames by using the read sequence in place of the annotated genome sequence, b) junctions are used to subdivide all contiguous read-covered regions to account for reads in introns which may arise from technical noise or from potentially meaningful biological heterogeneity. This results in the generation of all possible exons with read coverage, split across junction points. All junctions and exons are filtered according to count-based heuristics, which remove artifacts associated with intron definition (only the most probable intron endpoints are kept), read count (all exons and junctions must meet minimum requirements for read support), gaps in read coverage (internal reads are extended to fill gaps), and length (biologically unlikely introns with length > 1,000,000 nt are removed). Transcript assembly then proceeds in an ordered sequence for each exon, adding exons across junctions while optimizing for both read support and distance such that:

$$P_{i+1}=argmax_{p\in P(E_{i})}\left(\frac{C(E_{i},p)}{\sum_{f}C(E_{i},f)\sqrt{D(E_{i},p)}}\right),$$

$$E_{i+1}=argmax_{e\in E(P_{i+1})}X(e),$$

where ***C***(***E_i_,p***) is the read count supporting the junction from the current exon ***E_i_*** and the endpoint ***p***, ***D***(***E_i_,p***) is the distance of the junction from the end of ***E_i_*** to ***p***, ***P*_*i*+1_** is the start point of the new exon, ***E*_*i*+1_**, ***E***(***P*_*i*+1_**) is the set of all exons starting at ***P*_*i*+1_** and ***X***(***e***) is the length-normalized read count of exon ***e***. This process is repeated in this “greedy” fashion to generate the list of the most supported transcripts for any given starting exon while controlling for combinatorial explosion of the solutions using both probability-based filters when selecting candidate exons and endpoints, as well as by filtering the final transcripts. Transcript filtering is based on several factors: number of exons (transcripts are sorted by the number of exons they contain), average exon expression (transcripts must have an average normalized expression higher than a modulated threshold), and similarity to previous transcripts (transcript that show similar internal exon structure are filtered again based on a modulated threshold). These features of exons and transcripts were chosen as filtering criteria to ensure a level of read support for transcripts, while maximizing the diversity of the supported isoforms that were identified, and controlling for overall transcriptome size. The full MAPS code, sample dataset, and instructions are available from (http://www.bitbucket.org/shokhirev/MAPS)

### Testing assemblers using published and generated RNA-Seq

Simulated realistic hg19 reads were generated using the flux-simulator v1.2.1 tool[25] from the RefSeq hg19 gene set assuming 1e7 molecules, an expression K of 0 to increase the number of expressed genes, default reverse transcription, UR RNA fragmentation, default PCR amplification with an error rate of 0.05, no size filtering, 76 nt paired end reads, and 1e8 simulated reads, which were mapped and assembled using Cufflinks or MAPS. Published paired-end 100 nt reads from the Encyclopedia of Genomic Elements consisting of samples from various mouse tissues were mapped and assembled using Cufflinks and MAPS. Comparison to the mouse mm10 RefSeq transcript annotation was carried out by finding the total number of transcripts that were assembled uniquely by Cufflinks or MAPS, or by both assemblers. Since identifying the fraction of overlapping transcripts between two or more transcriptomes requires a threshold for transcript similarity, we defined a transcript that had at least one or more exons that overlapped with the exons of another transcript in a transcriptome as recovered/assembled.

### Microprotein annotation and hierarchical clustering

The average hg19 PhastCons [26] 100-way conservation value was calculated across all exon bases to define average conservation scores (value between 0 and 1000). Average RNA-Seq expression and Ribo-Seq expression values were calculated analogously using CPM normalized read counts from the HEK293T datasets described. All microproteins that started with a methionine start were annotated as having canonical start sites. Hierarchical clustering was carried out using the R v 3.2.2 using the heatmap.2 function assuming distance ***d*** = **1** − ***cor***(***M***), where ***M*** is a log-transformed matrix of the conservation, expression, and length of microproteins and linear AUG-start flag vector. Clustering was performed using the modified Ward method ward.D2.

### Cell culture and small proteome enrichment

HEK293 cells (GE Dharmacon cat#HCL4517) were cultured using DMEM supplemented with 10% fetal bovine serum (FBS). Cells were grown under an atmosphere of 5% CO_2_ at 37°C until confluent. Before cells lysis and enrichment of small proteome, the media was removed from adherent cells by aspiration HEPES-buffered saline (pH 7.5) was used to wash the cells to remove residual media and FBS. Small proteome was enriched from HEK293 cells using two complementary enrichment methods. 1) Cellular proteomes from 4 x 10^7^ cells were extracted by lysis with boiling water. After cooling the samples on ice, the cells were sonicated for 20 bursts at output level 2 with a 30% duty cycle (Branson Sonifier 250; Ultrasonic Convertor). Then the proteome was acid precipitated by addition of acetic acid (to a final concentration of 0.25% by volume), followed by centrifugation at 14,000 x g for 20 min at 4°C. This step precipitates larger proteins to reduce the complexity of the supernatant and enriches lower molecular weight proteins that are then analyzed by LC-MS/MS proteomics for microproteins. 2) Total proteome of 4 x 10^7^ cells were extracted using 50 mM HCl, 0.1% β-mercaptoethanol (β-ME); 0.05% Triton X-100 at room temperature (lysis buffer). The extracts were centrifuged at 25,000 g for 30 min, and supernatants filtered through 5 μM syringe filters. The flow through was then enriched for microproteins by binding and elution using Bond Elute C8 silica cartridges (Agilent Technologies, Santa Clara, CA). Approximately 100 mg sorbent was used per 10 mg total lysate protein. Cartridges were prepared with one column volume methanol and equilibrated with two-column volumes triethylammonium formate (TEAF) buffer, pH 3.0 before the sample was applied. The cartridges were then washed with two column volumes TEAF and the microprotein enriched fraction eluted by the addition of acetonitrile:TEAF pH 3.0 (3:1) and lyophilized using a Savant Speed-Vac concentrator. BCA protein assay (Thermo Scientific) was used to measure protein concentration of each sample after extraction and enrichment.

### Digestion and sample preparation for LC-MS/MS

An aliquot of 100 μg of enriched samples was precipitated with chloroform/methanol extraction. Dried pellets were dissolved in 8 M urea/100 mM TEAB, pH 8.5. Proteins were reduced with 5 mM tris 2-carboxyethylphosphine hydrochloride (TCEP, Sigma-Aldrich) and alkylated with 10 mM iodoacetamide (Sigma-Aldrich). Proteins were digested overnight at 37°C in 2 M urea/100 mM TEAB, pH 8.5, with trypsin (Promega) using 1 ug per 50 ug of protein (1:50). Digestion was stopped with formic acid, 5% final concentration.

### Q-Exactive mass spectrometry analysis

Digests were analyzed by LC-MS using an Easy-nLC1000 (Proxeon) and a Q Exactive mass spectrometer (Thermo Scientific). An EASY-Spray column (Thermo Scientific) 25 cm by 75 μm packed with PepMap C18 2 μm particles was used. Electrospray was performed directly from the tip of the analytical column. Buffer A and B were 0.1% formic acid in water and acetonitrile, respectively, and the solvent flow rate was 300 nl/min. Each sample was run in triplicate. The digested samples were loaded onto the column using an autosampler, and the samples were desalted online using a trapping column. Peptide separation was performed with 6-hour reverse phase gradient. The gradient increases from 5–22% B over 280 min, 22–32% B over 60 min, 32–90% B over 10 min, followed by a hold at 90% B for 10 min. The column was re-equilibrated with buffer A before injection. The Q Exactive was operated in a data-dependent mode. Full MS1 scans were collected with a mass range of 400 to 1800 m/z at 70k resolution. The 10 most abundant ions per scan were selected for MS/MS with an isolation window of 2 m/z and HCD energy of 25 and resolution of 17.5k. Maximum fill times were 60 and 120 ms for MS and MS/MS scans, respectively. An underfill ratio of 0.1% was utilized for peak selection, dynamic exclusion was enabled for 15s and unassigned and singly charged ions were excluded. Data was collected with sensitive setting for AGC of MS and MS/MS scans at 5e6 and 5e6, respectively, and maximum injection times of 120 ms and 500 ms for MS and MS/MS scans respectively.

### Data analysis to identify microproteins

Tandem mass spectra were extracted from raw files using RawConverter 1.0.0.0 and searched with ProLuCID using Integrated Proteomics Pipeline–IP2 (Integrated Proteomics Applications). We used four custom databases created from the in silico 3-frame translation of RNA-Seq data from HEK293 cells described above. The search space included all fully-tryptic and half-tryptic peptide candidates. Carbamidomethylation on cysteine was considered as a static modification. Data files from technical triplicates were combined and searched by ProLuCID. Data was searched with 50-ppm precursor ion tolerance then filtered to 10-ppm, and 50-ppm fragment ion tolerance with a maximum of two internal missed cleavages using the custom databases. Identified spectra were filtered and grouped into proteins using DTASelect. Proteins and microproteins required, at least, one peptide to be identified with a setting of less than 1% FDR.

To identify microproteins, data files from technical duplicates were combined and searched by ProLuCID. Data was searched with 50-ppm precursor ion tolerance then filtered to 10-ppm fragment ion tolerance with a maximum of two internal missed cleavages using only the custom database. The results from the custom database search were then filtered against the Swiss-Prot release date December 2013 human protein database using a string-searching algorithm to remove any annotated peptides. We visually inspect the MS2 spectra for all the smORF/microprotein peptides to validate the assignment. In particular, we required that any critical amino acid residues that uniquely distinguish the peptide detected in the MS2 data.

The next step is to determine whether the non-annotated peptides are from smORFs or not. The non-annotated peptides are searched against NCBI Human Reference Sequence Database (RefSeq) using tBLASTn, which identifies an annotated transcript that could have produced the microprotein. After identifying an RNA and sequence that encodes the peptide, we annotate the downstream in-frame stop codon, and then try to identify the upstream in-frame start codon.

We assign start codons to any in-frame ATG. If there is no in-frame ATG, we look for an in-frame near-cognate codon (i.e. ACG, AAG, CUG, etc.) in a Kozak sequence to assign as the start codon. Lastly, if an in-frame ATG or near-cognate start codon cannot be found, we identify the upstream in-frame stop codon, and if the distance between the upstream and downstream in-frame stop codons is less than 150 codons, we annotated the gene as a smORF. If the peptides did not match to any RNA sequences with the RefSeq RNA database, it means that they were derived from RNAs that were present in the RNA-Seq data but not in the RefSeq database. For these peptides, we repeat these steps for assigning the smORF using RNAs from the RNA-Seq database. Full list of microproteins detected are presented in S1 Table. For candidate verification, synthetic peptides were purchased from JPT at ~30 nmol crude. The peptides were reconstituted in water and mixed to have final concentration of 50 fmol/ul for each peptide in the mixture and ProLucid was used to search the synthetic data with the same parameters as described above. MS2 spectra of the synthetic peptides were manually validated to show that the fragmentation patterns are the same as the endogenous peptides detected. Annotated MS-Spectra and corresponding spectra from synthetic controls are shown in Fig F in S1 File.

Additional methods details are described in previous studies [8,9].

## Results and discussion

### MAPS for tunable mRNA transcript assembly

Proteogenomic identification of novel microproteins relies on identifying putative small open reading frames (smORFs) from assembled transcripts, which can be *in silico* translated and cross-referenced against measured mass spectra. Inspiration for MAPS came after we tested the overall quality of assemblies from several generalized transcript assemblers, Cufflinks [19], genome-guided Trinity [27], and StringTie [28] on our high-depth 293T RNA-Seq datasets (Paired-end 150, ~ 500 million reads). After optimizing each assembler for our data, we found that while in general all three assemblers were able to reconstruct annotated highly expressed transcripts, there were many examples where low-expressed transcripts were misassembled (Fig A in S1 File). Given that these assemblers are not designed explicitly for proteogenomics and had trouble assembling low-abundance annotated transcripts, we developed a novel transcript assembler, MAPS, which we designed from the bottom-up to assemble RNA-Seq data into a collection of ORFs while optimizing for both accuracy and transcriptome diversity (Fig 1A). We selected Cufflinks as a comparison because of its popularity and overall ability to assemble transcripts across a wide range of read coverages. While the goal of this study is to point out the importance of transcript assembly features in the context of proteogenomic discovery of microproteins, an in-depth analysis across a panel of assemblers and across multiple proteogenomics datasets is outside the scope of this study.

Extant de novo and genome-guided assemblers such as Cufflinks, Trinity, or StringTie were developed for transcript identification not proteogenomics. As a result, these general-purpose transcript assemblers minimize false-positives of the assembly at the cost of transcriptome diversity and recall [28]. For example, Cufflinks finds the minimal set of transcripts that describe the reads, omitting possible rare transcripts, which may encode microproteins [19]. Furthermore, they specifically remove typical experimental artifacts, which further limits the diversity of the assembled transcriptome. For example, Cufflinks removes low-abundant transcripts with retained introns [19].

In addition, general-purpose assemblers require post-processing to generate peptide databases that can be used for proteomics searching. We hypothesized that incorporating information from the read and flanking genome sequence should improve the accuracy of the *in silico* translated peptides. For instance, some cell lines might contain point mutations in genes leading to differences in the sequence of a gene from RNA-Seq data from the sequence in the reference genome. For proteogenomics, genome mutations will lead to changes in the proteome and should be incorporated during *in silico* 3-frame translation of assembled transcripts to accurately reflect cell line specific peptides, and avoid misclassifying mutations in known proteins as novel microproteins.

In addition, partial sequence coverage of low-expressed genes is a common problem for mRNA-Seq [29]. As a result, extending assembled open reading frames up- and down-stream at the ends of transcripts can help improve coverage by using the annotated genome sequence to help impute ORF sequence to the nearest stop codon.

Finally, by design Cufflinks, StringTie, Trinity, and other generalized assemblers do not explicitly control for the assembled transcriptome size, which is crucial for downstream proteomics searching. All parameters must be manually optimized for each dataset to avoid biasing downstream searching, while maintaining accurate transcript reconstruction.

**Fig 2:**
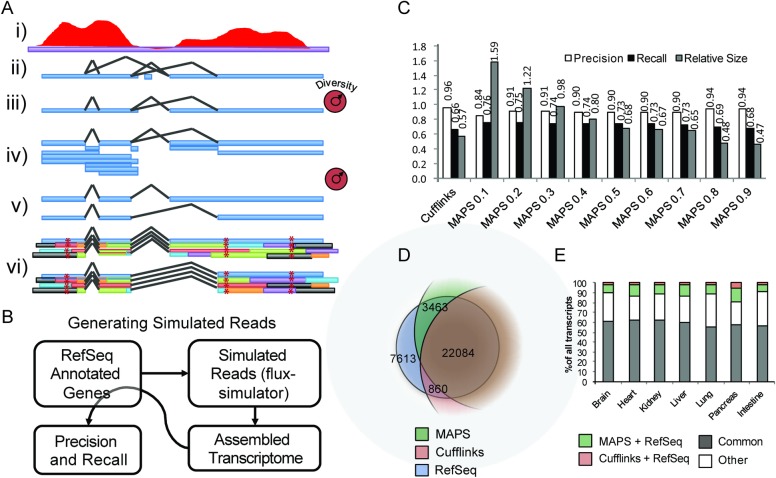
Building and testing a tunable ORF assembler. A) Overview of MAPS, A novel tunable ORF assembler. High-depth paired-end reads (i) are used to identify all possible supported contigs and junctions (ii), which are then filtered (iii) and used to generate all possible supported exons (iv). Transcripts are then assembled using greedy heuristics and filtered based on read support (v). The resulting transcripts are then *in silico* translated for all frames to produce a set of supported ORFs using the consensus read sequence and the annotated genomes to further extend the ORFs (vi). B) Workflow for determining precision and recall of the assembled transcripts using realistic simulated RNA-Seq reads generated from annotated RefSeq genes. C) Simulated reads were assembled using a generalized assembler, Cufflinks, and MAPS with varying stringency (diversity) settings, and the precision, recall, and relative size of the resulting transcriptomes are shown. D) Plot showing the relative fractions of RefSeq transcripts assembled with MAPS (Stringency = 0.3) and Cufflinks using the simulated reads workflow in B). E) ENCODE paired end mouse RNA-Seq datasets for varying tissues were assembled using MAPS and Cufflinks and fractions of shared transcripts are shown. Colors represent transcripts overlapping between just MAPS assembly and RefSeq annotated genes (green), transcripts overlapping between just Cufflinks and RefSeq annotated genes (pink), transcripts overlapping between RefSeq, MAPS, and Cufflinks (gray), and all other transcripts that are assembled but not annotated in RefSeq (white).

Therefore, we developed a new assembler called mRNA Assembler for ProteogenomicS (MAPS) to explore the influence of these considerations on microprotein discovery (Fig 2A). From RNA-Seq mapped reads, MAPS assembles a collection of open reading frames with a tunable stringency (transcriptome diversity). Specifically, the diversity of the assembled transcriptome is controlled by filtering the assembled exons and junctions based on the read support, by filtering how many transcripts are assembled using the remaining exons and junctions, and by filtering the final set of assembled transcripts based on similarity to other assembled transcripts. With this feature, MAPS optimizes for read support as well as transcriptome diversity. This is a key feature for downstream proteomics searching, which scores potential peptide candidates on their spectral similarity to all other assembled peptides [30]. Further, MAPS includes *in silico* translation and uses the read sequence to account for sample-specific mutations (i.e. mutations in a cell line or tissue) providing a more accurate representation of the proteome in the resultant database. MAPS also extends assembled open reading frames up- and down-stream using the annotated genome sequence to enable the detection of microproteins from smORFs at the ends of poorly expressing transcripts. Similar to Cufflinks and other genome-guided assemblers, MAPS relies on state of the art read mappers such as STAR to solve the difficult problem of read alignment to the genome.

We next setup a global test for transcript assembly using a simulated RNA-Seq dataset, generated from the human RefSeq hg19 to mimic real RNA-Seq data and enable us to test the performance of the different assemblers using the flux-simulator pipeline [25]. The data from flux-simulator is assembled using MAPS and Cufflinks (Fig 2B). We evaluated the assemblers by checking for any exon overlap between the assembled transcripts and the reference human genes that were used to simulate the reads, and found that there was a higher fraction of overlap between MAPS assembled transcripts and human transcripts compared to Cufflinks assembled transcripts. (Fig 2C, dark bars). Furthermore, we found that modulating the internal diversity parameter of MAPS allowed us to find a balance between recall (TP/TP+FN), precision (TP/TP+FP), and transcriptome size, and selected a MAPS stringency value of 0.3 for these tests because it resulted in a transcriptome comparable to the reference human transcriptome. The tradeoff for a higher recall was a lower precision, which we felt was acceptable since we use proteomics to verify protein-coding transcripts. Using this selected stringency parameter of 0.3, MAPS could assemble 3,463 more transcripts using the reads generated from the RefSeq dataset. Compared to that, Cufllinks only generated 860 additional transcripts using the same dataset (Fig 2D).

We also obtained, mapped, and assembled a collection of mouse RNA-Seq datasets from ENCODE using both Cufflinks and MAPS, and quantified the overlap of each assembly as compared to the annotated mm10 RefSeq transcriptome (Fig 2E). The results show that for all mouse tissues, MAPS recalls a higher percentage of the annotated mouse genes (green bars), compared to Cufflinks assembly (red bars). Therefore, we concluded that MAPS assembles a higher fraction of the known transcripts in both simulated and biological high-quality RNA-Seq datasets.

### Using MAPS for microprotein discovery

We utilized MAPS and Cufflinks to analyze two proteomics datasets collected from HEK293T cells [17] to detect microproteins. The two datasets published were from the same cells but underwent different enrichment conditions to separate microproteins from the rest of the proteome [17]. For one dataset microproteins were enriched using the acid precipitation method (ACID) that kept microproteins in solution. In the second dataset, a C8 solid phase extraction (C8-SPE) column was used to enrich microproteins (Fig B in S1 File).

**Fig 3:**
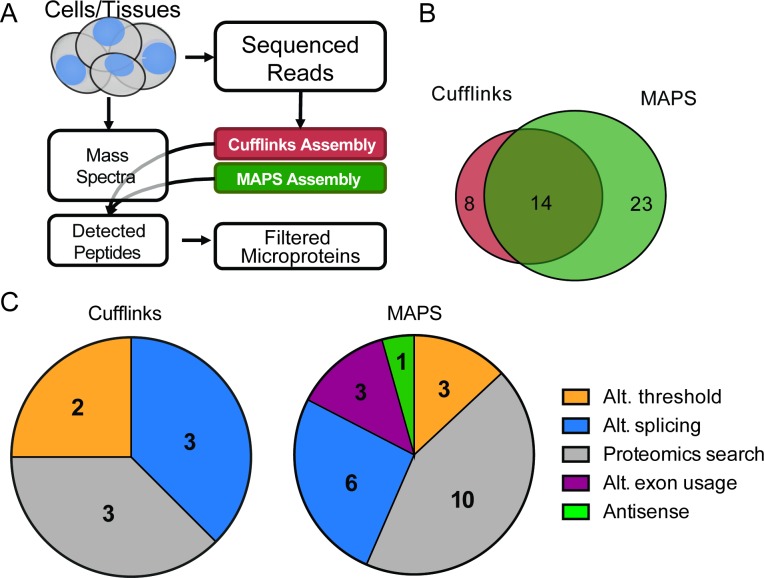
MAPS and Cufflinks find complementary micropreteins. A) Proposed microprotein discovery workflow incorporating Cufflinks and MAPS assemblies to improve microprotein discovery B) Total number of unique curated microproteins detected by mass spectrometry in HEK293T cells. Cell lysate was enriched using ACID or C8-SPE Column (data combined), and proteomics data was searched against Cufflinks or MAPS assembled transcriptome that was translated in three frames. C) Distribution of microproteins uniquely detected in Cufflinks database search (eight microproteins, left) and MAPS database search (23 microproteins, right), describing reasons why they were not detected in the alternate. Alt. Threshold: microprotein-containing transcript was filtered from one assembly while kept in the other due to differences in abundance thresholds, Alt. splicing: microprotein was translated from a transcript with alternate splicing event, Proteomics search: microproteins were not detected due to similar sequence present during MS search, Alt. exon usage: microproteins arise from extension of exons, Anti-sense: transcript on only one strand is assembled.

We analyzed these proteomics datasets with a custom 3-frame translated database that came from RNA-Seq data that was assembled using Cufflinks or MAPS (Fig 3A). Using the Cufflinks transcriptome, we identified 3,688 and 5,840 unique peptides from the ACID and C8-SPE proteomics datasets, respectively (Fig C panel A in S1 File). With the MAPS-assembled database, we identified 3,713 and 5,968 unique peptides from the ACID and C8-SPE proteomics datasets, respectively (Fig C panel A in S1 File). Most of the detected peptides were shared between the MAPS-derived and Cufflinks-derived databases, with 81% (ACID) and 80% (C8-SPE) of the total detected peptides shared between the MAPS- and Cufflinks-derived databases (Fig C in S1 File). This large overlap in the overall detected peptides suggested that in general both MAPS and Cufflinks could reconstruct comparable protein databases for proteomics searching.

We observe the biggest differences between MAPS and Cufflinks when we look at novel microproteins. Combining the ACID and C8-SPE results, there are a total of 37 new microproteins detected using MAPS, and 22 non-annotated microproteins identified by using Cufflinks (Fig 3B). Unlike the case with the entire proteome, where 80% of the peptides were detected by MAPS and Cufflinks, only 14 novel microproteins out of a total of 45 overlapped between the MAPS and Cufflinks database searches (31%). Furthermore, 23 microproteins were only detected using the MAPS database and eight were only detected by Cufflinks (Fig 3B).

**Fig 4:**
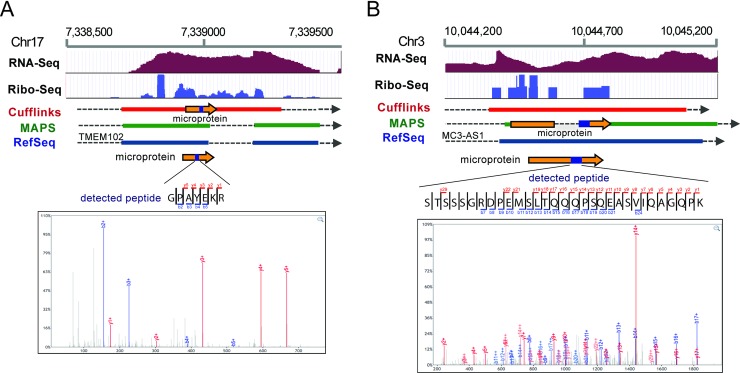
Examples of two microproteins that were uniquely detected in the Cufflinks or the MAPS (right) assemblies. Tracks show HEK293T RNA-Seq, Ribo-Seq read coverage and RefSeq genes, Cufflinks and MAPS assembled transcripts, colored boxes represent exons, dotted lines represent intronic regions, and double white arrows represent continuation of the transcript. Orange arrow represents where microproteins are translated and a blue box within the arrow shows where the detected peptides are. Detected peptides and their MS2 spectra are shown. A) An example of a microprotein that was uniquely detected in Cufflinks assembled transcriptome but not MAPS. Cufflinks assembled a transcript that is unique microprotein translation, where part of the microprotein extends into intronic region of an annotated gene. B) An example of a microprotein that was uniquely detected in MAPS assembled database. The microprotein is translated from a transcript with alternate splicing of a known gene assembled by MAPS, while Cufflinks failed to assemble this transcript.

These differences emerged for several reasons. Of the eight Cufflinks-specific microproteins discovered, two did not make it into the MAPS assembly because they came from short single-exon transcripts (<1000 bp), which are required to have a higher read support in MAPS compared to longer or multi-exon transcripts (Fig 3C, left). This filter was added to MAPS to dramatically decrease the database size, which would otherwise consist primarily of single-exon low-abundance transcripts. Three of the other Cufflinks-specific microproteins were from alternate exon assemblies, where an alternate splice junction was found, that was specific to the Cufflinks assembly (Fig 3C, left). The last three microproteins that were only detected in the Cufflinks analysis were part of the MAPS assembly but not called during the proteomics search due to the presence of similarly scoring peptides (Figs 3C and 4A).

In comparison, only three of the MAPS-specific microproteins were not present in the Cufflinks assembly due to low read count, while six had unique splicing patterns due to alternate junction usage (Fig 4B), three came from assembly of a longer exon, 10 were present but not detected during the proteomics search likely due to the presence of similarly scoring peptides being assembled (Fig 3C, right). Finally there was one MAPS-specific microproteins detected that is coded for by an anti-sense transcript coming from the opposite strand, which was not part of the Cufflinks assembly (Fig 3C, right).

**Fig 5:**
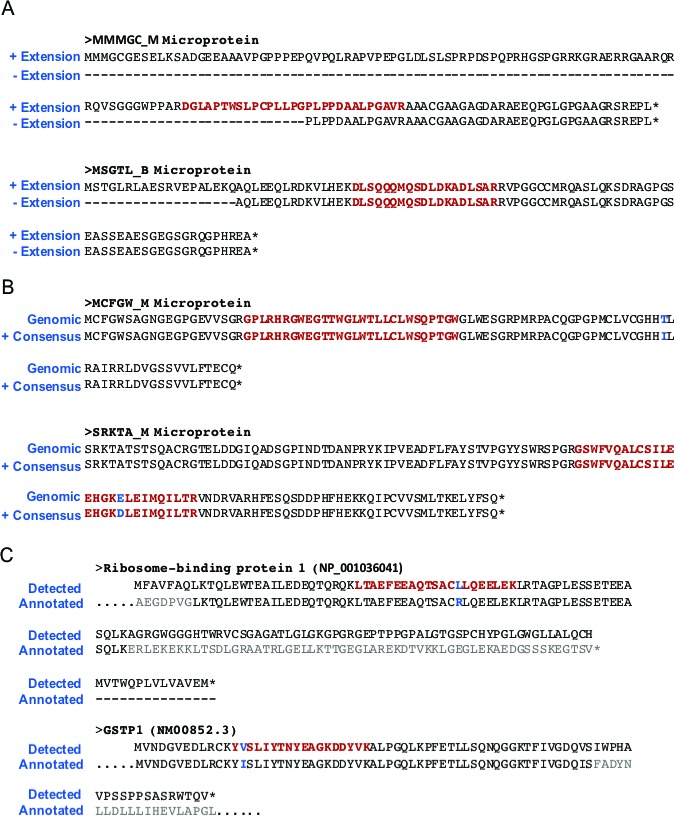
Examples of improvement in accuracy of microprotein detection due to ORF extension and consensus read sequence. A) Examples of ORF extension improving the accuracy of detected microproteins. Without extension, a truncated microprotein would be inaccurately reported with a non-AUG start site. B) Examples of cell-line specific SNPs improving the accuracy of detected microproteins. C) Examples where cell-line specific SNPs resulted in the filtering of two seemingly novel SEPs, which are actually cell-line specific mutants of annotated proteins.

To further explore the benefit of extending open reading frames up or downstream to the next stop codon, as well as the benefit of using the read consensus sequence to account for cell-line specific mutations, we tested what would happen if we removed these features from the MAPS assembly process. When extending ORFs to the nearest STOP codon up and downstream, we observed that 3 of the detected microproteins were directly affected. One microprotein was detected only because the ORF extension included the detected LC-MS peptide, and the two other microproteins were identified because the extension included an upstream methionine start site (Fig 5A). In addition, there were five microproteins detected in the MAPS assembly due to mutations in the HEK293T cells that differed from the reference genome. One of these microproteins was novel and would not have been detected otherwise (Fig 5B), while the four others were cell line specific mutants of annotated proteins, differing by one amino acid from annotated sequence but originating in frame of an annotated exon, which allowed us to remove them from our final curated list as false positives (Fig 5C). Furthermore, while the detected peptide did not contain an SNP, using the read consensus sequence allowed us to correct the sequence of 2 detected microproteins (Fig 5B, MCFGW_M microprotein).

### Analysis of detected smORFs and microproteins

**Fig 6:**
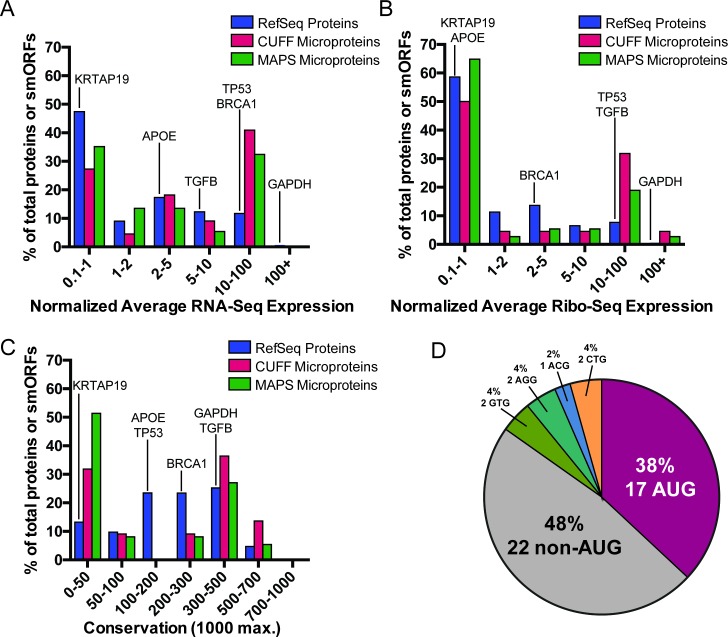
smORF/microprotein annotation. A-B) Distribution of total normalized expression in A) RNA-Seq and B) Ribo-Seq datasets. C) Distributions of average PhastCons 100-way conservation scores are shown. D) Proportion of canonical AUG start sites for detected microproteins. A near cognate start codon (one nucleotide difference from AUG) was assigned if it was in a Kozak consensus sequence. Example RefSeq genes are highlighted for reference.

We next evaluated the length, expression, ribosome occupancy, conservation, and start-codon presence of the 45 smORFs detected with the new pipeline. In general, detected smORF RNAs have average expression levels similar if not higher than those of average RefSeq genes, and there was no detectable difference in expression level between smORF RNAs detected using MAPS or Cufflinks assembly approaches (Fig 6A). Detected smORFs also showed similar or higher levels of average normalized Ribo-Seq occupancy compared to RefSeq annotated genes (Fig 6B).

While most RefSeq genes show a relatively flat distribution of average PhastCons 100-way conservation scores between 0 and 500, smORFs of detected microproteins tended to have either low (0–50, compare to KTRAP19) or high (300–500, compare to GAPDH or TGFB) average conservation scores, and smORFs discovered using the MAPS assembly tended to have more low-conservation scores compared to smORFs discovered using the Cufflinks assembly (Fig 6C). Similar to other smORFs reported in previous studies, we find that two-thirds of smORFs encoding detected microproteins do not have an AUG start codon [7, 8, 17] (Fig 6D). Finally, while we detected microproteins between 16 and 149 aa using our microprotein discovery pipeline, the median microprotein length was 62 amino acids and more than two thirds of microproteins were less than 100 amino acids in length (Fig C in S1 File).

Next, since there were 14 microproteins that were detected using both assembly methods, we wanted to further explore how the characteristics of these overlapping microproteins differed from microproteins in general. Our analysis revealed that while most microprotein mRNAs tend to have a similar distribution of RNA-Seq expression and normalized Ribo-Seq expression compared to the distributions of annotated RefSeq genes, the overlapping smORFs tend to have higher RNA-Seq and Ribo-Seq normalized average expression (Fig D in S1 File).

Microprotein analysis using a hierarchical clustering approach (Fig E in S1 File) revealed primarily two groups of microproteins: those that have high levels of conservation and expression, and tend to start with a canonical AUG, and those that show low levels of conservation, expression, and tend to start from non-canonical codons, suggesting that microproteins may play both general well-conserved roles, or specialized roles as all detected microproteins had RNA-Seq and proteomics support. We also found that conserved microproteins separate into three main groups: those with moderate length and high RNA-Seq and Ribo-Seq coverage; those that are short, have relatively low expression; and those that are long and have moderate levels of expression. While most of the microproteins detected using both Cufflinks and MAPS-derived assemblies tend to have higher expression and are more highly conserved on average (9/14), there were five that showed relatively low levels of expression and three that showed very low conservation levels, suggesting that this variability in microprotein detection is due to more than just assembler choice. Annotated MS2 spectra for all the detected unique peptides have high sequence coverage (Fig F in S1 File), finally we also purchased synthetic peptides to validate some detected peptides by MS2 spectral matching (Fig F in S1 File).

## Conclusions

Microproteins represent a novel class of potentially functional peptides and small proteins which have already been shown to be essential for fly development [1, 3], mitochondrial function [31], and muscle function [12, 32]. Discovering microproteins and the smORFs that encode them is challenging, however, because smORFs can start from non-canonical codons, may be translated from the same genomic sequence as well-characterized annotated ORFs, and microproteins are difficult to detect because they only generate a few peptides for proteomics detection and are at low levels. Thus, a reliable microprotein and smORF strategy is a continuing problem. Computational studies have predicted that potentially thousands of microproteins may be lurking in the human genetic code based on sequence conservation studies [5]; however, these predicted smORFs likely contain false positives. Since we are interested in characterizing the biological activity of microproteins, we believed that the microproteins of the greatest interest to us would be stable and long-lived enough to be detected by proteomics. Therefore, we opted for a proteogenomics approach since this would identify stable microproteins, and simultaneously validate the translation of a smORF. We have optimized several experimental steps in this platform including the microprotein isolation and fractionation and mass spectrometry proteomics methods to identify additional microproteins and smORFs [8, 17].

General-purpose transcript assemblers are designed to provide a picture of the transcriptome and contain several key steps to minimize false positives [33]. For example, for a given threshold, Cufflinks produces only the most-supported transcripts, often omitting other possible but less likely transcripts. While this ensures that abundant transcripts are assembled, rare transcripts that have an alternate splice pattern and lower abundance may be missed.

We hypothesized that an ORF assembler that optimizes for transcriptome diversity and takes the sequence information into account could provide the ability to discover additional smORFs and microproteins by proteogenomics. To test this, we developed MAPS, a novel assembler for proteogenomics applications (Fig 2A). The two primary differences between MAPS and general-purpose assemblers is the optimization for transcriptome diversity during transcript assembly, thereby allowing MAPS to find rare transcripts, and the additional post-processing steps that utilize mRNA sequence in conjunction with genomic sequence to account for cell-line specific mutations and extend open reading frames.

In addition, by not imposing assumptions on the start codon usage, conservation, or peptide usage a priori, instead relying directly on the data to build a case for each peptide, MAPS produces a list of short non-annotated smORFs that are validated as microproteins by high-quality proteomics. Our approach revealed that MAPS (Fig 2A) assembles a larger proportion of the true transcriptome while controlling for transcriptome size at the cost of a slightly lower precision (i.e. more false positives). Also, MAPS tends to recover a higher proportion of the mouse genome from publically available ENCODE RNA-Seq datasets compared to a general-purpose assembler (Fig 2). The greater number of false positives is tolerable because the result of the pipeline is a microprotein, such that every smORF will have genomic and proteomic data to support its existence and translation. This means that MAPS may be more appropriate when one is looking to assemble low abundant or alternatively spliced transcripts from the RNA-Seq data by relying on orthogonal datasets to control the false-positive rate (i.e. proteomics or Ribo-Seq).

Our data shows that optimization of transcriptome diversity, extension of open reading frames up- and down-stream based on genomic sequence, and using SNP information encoded in the RNA-Seq reads, as well as applying multiple assemblers can increase the number of microproteins discovered, the goal underlying the development of MAPS in the first place. Most assembled peptides are identical between Cufflinks and MAPS (Fig C in S1 File), but MAPS does find an additional 23 microproteins compared to Cufflinks (23 vs 8). Combining both assemblies further expanded the pool of microprotein candidates to 45, 37 from MAPS and 22 from Cufflinks, with 14 assembled by both (Fig 3 and S1 Table). In addition to microproteins arising from novel splice-isoforms (Figs 3C and 4), there were three microproteins which were discovered due to use of an extended exon, three that did not pass the Cufflinks abundance thresholds, and one that came from an anti-sense transcript being assembled by MAPS (Fig 3C), suggesting that this approach may be better suited for finding proteins coded by unconventional ORFs. Imputing the full length ORF by extending the sequence to the nearest stop codon up- and down-stream also improved MAPS microprotein discovery and allowed more accurate identification of the start codon (Fig 5A). In addition, we observed that taking the read sequence into account improves both microprotein discovery, and potential downstream validation, enabling cell-line specific microprotein detection (Fig 5B and 5C). Combined with results from the general-purpose Cufflinks assembler, we feel that MAPS improved the accuracy of our proteogenomic microprotein discovery pipeline.

Our analysis of the identified microproteins revealed dichotomies in the characteristics of detected microproteins. We observed microproteins that had either low or uncharacteristically high expression (both mRNA and ribosome footprinting), as compared to all RefSeq annotated genes (Fig 6A and 6B). We also observed that large proportion of microproteins showed relatively low conservation scores compared to typical genes, while another set seemed to show higher than average conservation signatures, again suggesting that there may be two classes of microproteins detected. Furthermore, we again confirmed that many microproteins that were detected did not start with an AUG start codon, suggesting that alternate codon usage may play an important role in microprotein translation and further highlighting the importance of an unbiased search (Fig 6D). Finally, we reveal that microproteins detected with both assembly methods are typically more conserved and have higher average expression (orange bars, Fig D in S1 File), but include microproteins that are poorly expressed and conserved (Fig E in S1 File), further highlighting the need for specialized assembly methods and orthogonal dataset cross-validation for novel microprotein discovery.

Importantly, while the pipeline was designed for short peptide discovery, it can be arbitrarily adapted for use for detecting longer proteins, and the new MAPS tool can be directly applied to non-coding RNA detection, rare isoform detection, and novel transcript detection, and includes standard features for transcript abundance estimation and SNP detection. Moving forward, directly integrating LC-MS/MS spectral matching into MAPS and the incorporation of other pre-filtering steps should further improve microprotein detection.

## Supporting information

S1 File Supporting figures. Figures A-F.
(PDF)

S1 Table Full list of 45 microproteins detected. Excel file containing annotated list of microproteins detected.
(XLSX)
