# Supplementary material for: The influence of transcript assembly on the proteogenomics discovery of microproteins
Source: PLoS One. 2018 Mar 27;13(3):e0194518. doi: 10.1371/journal.pone.0194518 (PMC5870951; doi:10.1371/journal.pone.0194518)
Supplement: S1 File — Figures A-F. (PDF) [file pone.0194518.s001.pdf]

Supporting materials for:

The Influence of Transcript Assembly on Proteogenomics Discovery of Microproteins

Jiao Ma[1], Alan Saghatelian[1], Maxim Nikolaievich Shokhirev[1,2*]

[1] Clayton Foundation Laboratories for Peptide Biology, Salk Institute for Biological Studies, La Jolla, CA, USA

[2] Razavi Newman Integrative Genomics and Bioinformatics Core, Salk Institute for Biological Studies, La Jolla, CA, USA

*Corresponding author

Email: mshokhirev@salk.edu

This PDF includes:
Figures A-F

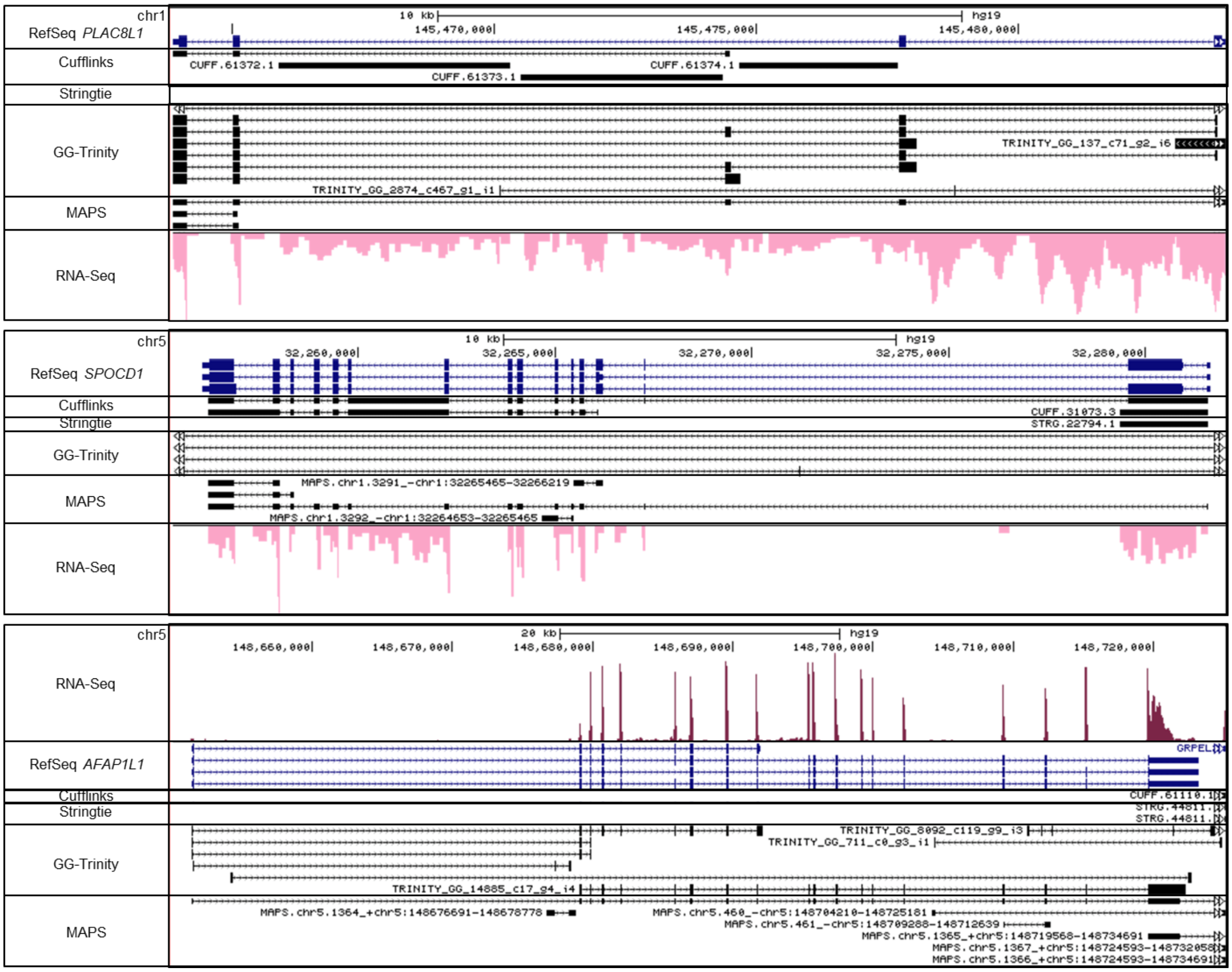

Fig A: **Motivating Examples: Assembly of the *PLAC8L1*, *SPOCD1*, and *AFAP1L1* genes.** High quality RNA-Seq data from HEK293T cells was used to assemble transcripts using Cufflinks (83,930), Genome-guided Trinity (GG-Trinity) followed by GMAP alignment to the human genome (81,410), StringTie (86,123), and the mRNA assembler for proteogenomics, MAPS (85,124). Tracks show the normalized RNA-Seq read densities, RefSeq annotated gene structures, and assembled transcripts. Boxes represent exons, and lines represent introns. Double arrows indicate that transcripts continue beyond the plot boundaries.

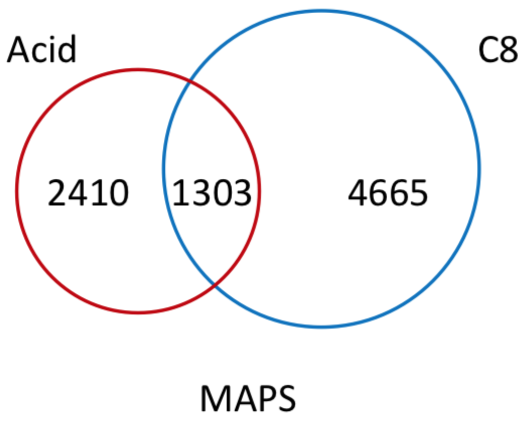

Fig B: Acid and C8 sample preparation results in complementary peptide detection. Venn diagrams show the number of unique detected in acid precipitated and C8 column enriched samples searched against Cufflinks and MAPS assembled custom databases. Please see ref 17 for additional information.

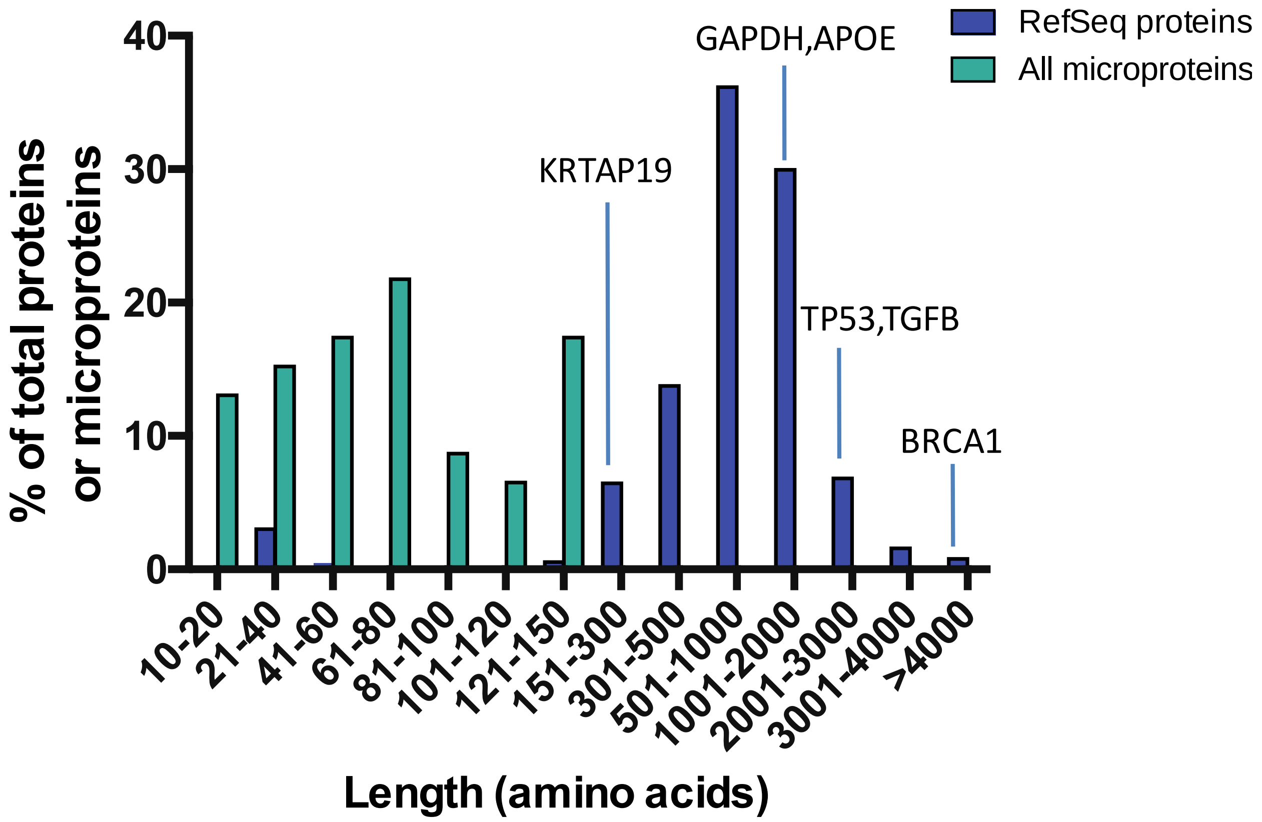

Fig C: **Microprotein length distributions compared to all RefSeq annotated proteins.**

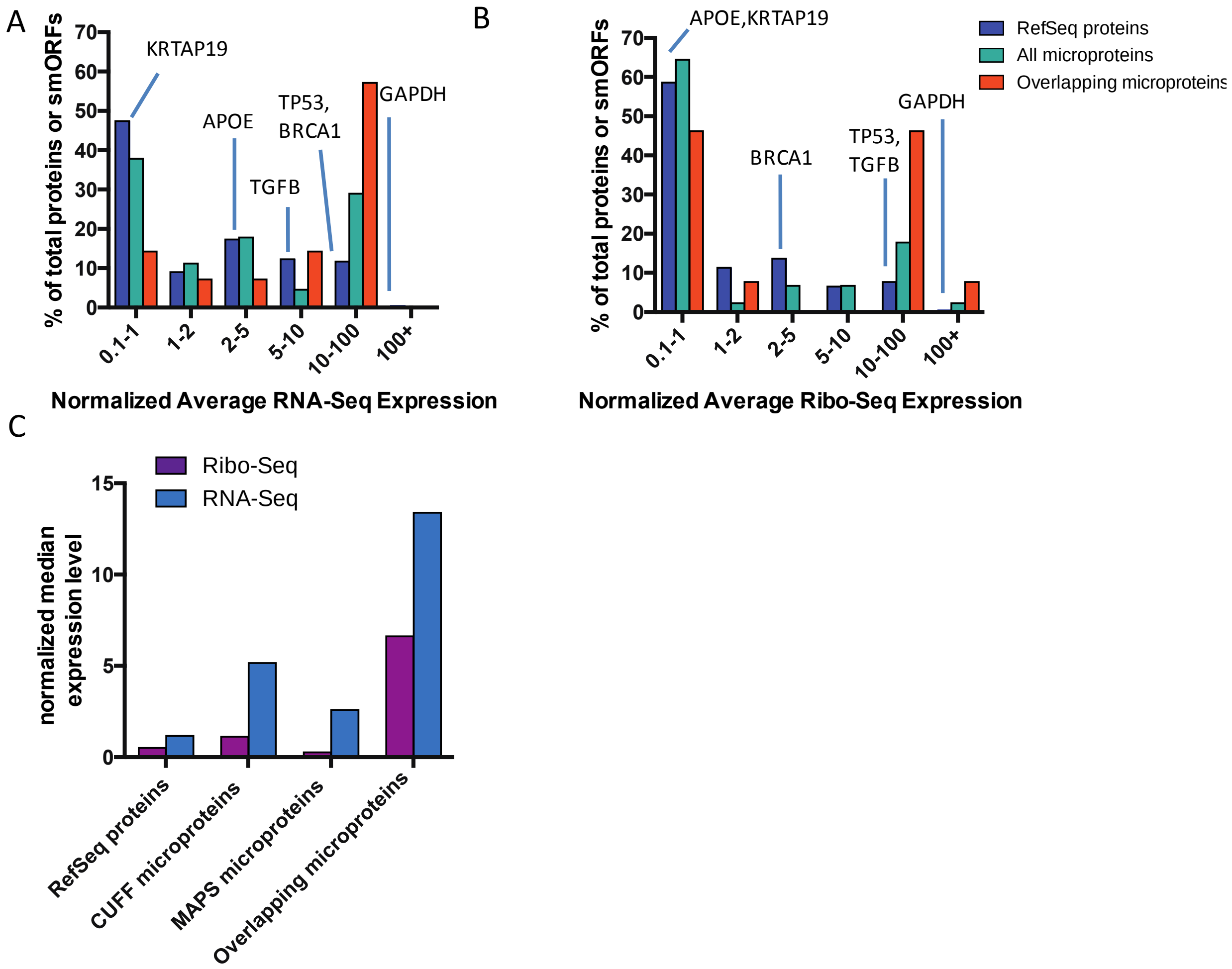

Fig D: Microproteins detected in both assemblies show higher expression. A,B) Distributions of normalized average RNA-Seq and Ribo-Seq expression for all RefSeq protein-coding genes, all microproteins, or microproteins detected in both assemblies. C) Plot showing normalized median RNA-Seq and Ribo-Seq expression for all RefSeq genes, Cufflinks microproteins, MAPS microproteins, and overlapping microproteins.

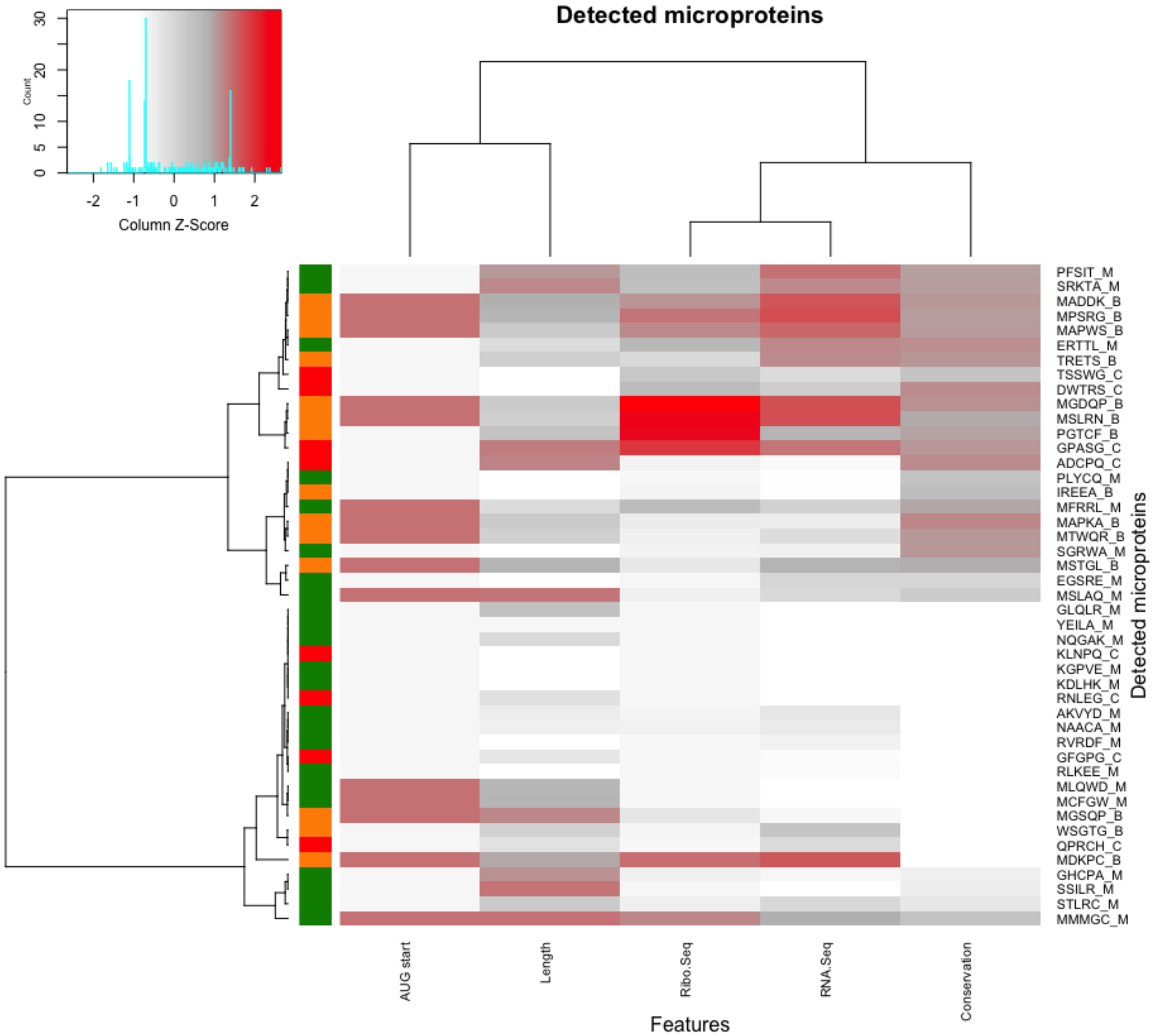

Fig E: Clustering of all detected microproteins by feature. Hierarchical clustering of all microproteins by normalized log-transformed conservation score, normalized log-transformed RNA-Seq expression, normalized log-transformed Ribo-Seq expression, normalized log-transformed length, and the presence of an AUG start. Clustering is based on correlation of these features across microproteins. Red indicates higher than average (z-normalized) values while white indicates lower than average values. Microproteins are labeled according to the first 5 aa in the protein and whether it was detected in the Cufflinks-derived assembly (C), MAPS-derived assembly (M), or both (B).

Experimental

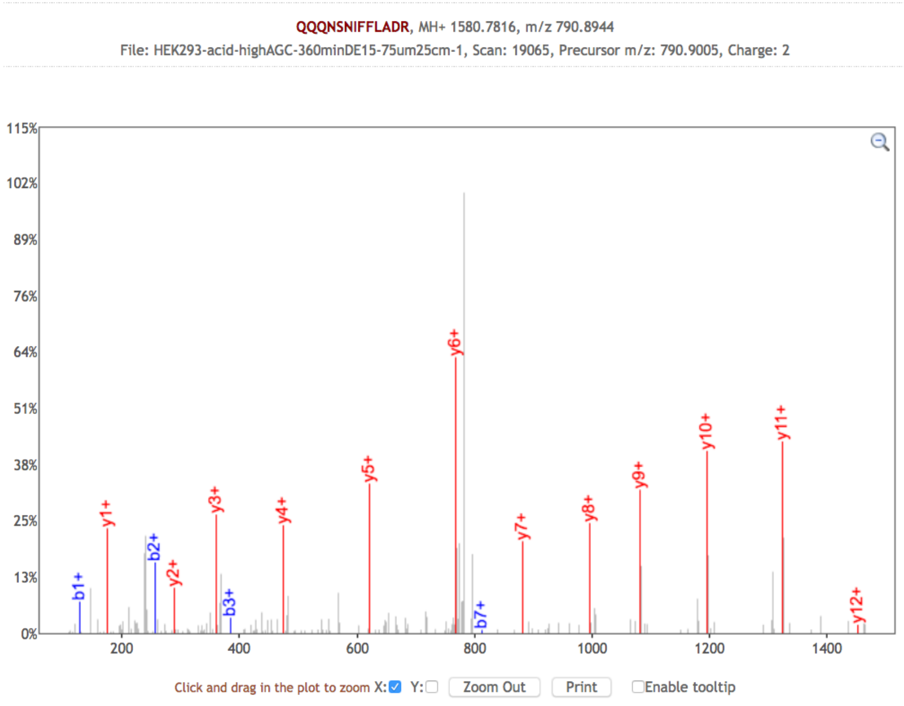

b+	#	Seq	#	y+
129.0659	1	Q	13	
257.1244	2	Q	12	1452.7230
385.1830	3	Q	11	1324.6644
499.2259	4	N	10	1196.6058
586.2580	5	S	9	1082.5629
700.3009	6	N	8	995.5309
813.3850	7	I	7	881.4880
960.4534	8	F	6	768.4039
1107.5218	9	F	5	621.3355
1220.6058	10	L	4	474.2671
1291.6430	11	A	3	361.1830
1406.6699	12	D	2	290.1459
	13	R	1	175.1190

[Click] to move table

Static Modifications:
C: 57.02146

Synthetic

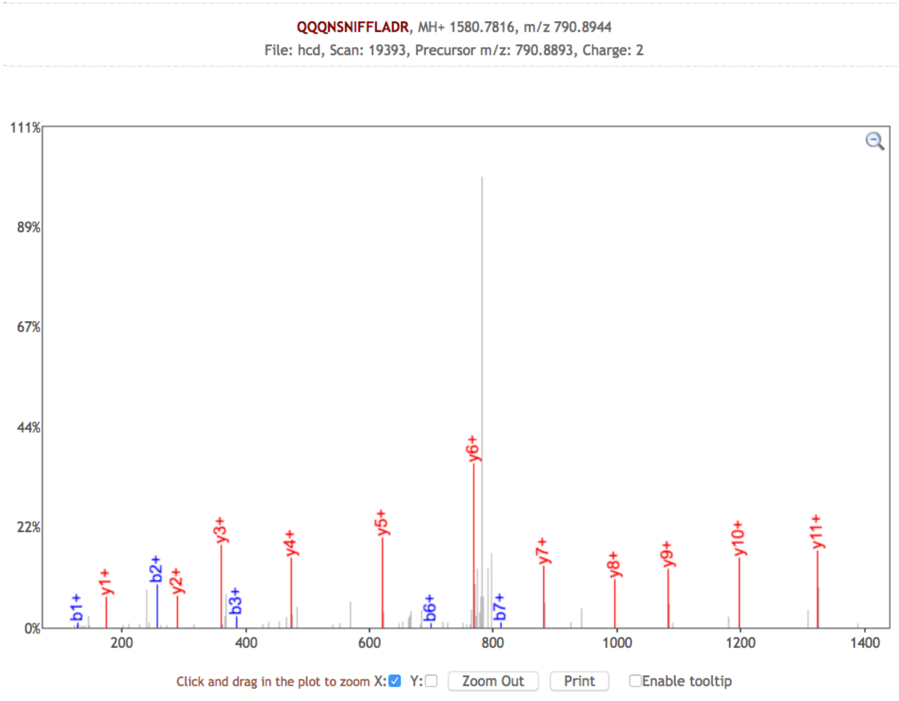

b+	#	Seq	#	y+
129.0659	1	Q	13	
257.1244	2	Q	12	1452.7230
385.1830	3	Q	11	1324.6644
499.2259	4	N	10	1196.6058
586.2580	5	S	9	1082.5629
700.3009	6	N	8	995.5309
813.3850	7	I	7	881.4880
960.4534	8	F	6	768.4039
1107.5218	9	F	5	621.3355
1220.6058	10	L	4	474.2671
1291.6430	11	A	3	361.1830
1406.6699	12	D	2	290.1459
	13	R	1	175.1190

[Click] to move table

Static Modifications:
C: 57.02146

Experimental

RDPWYSTVGLLPPVR, MH+ 1755.9541, m/z 585.9895

File: HEK293-acid-highAGC-360minDE15-75um25cm-1, Scan: 23596, Precursor m/z: 585.9931, Charge: 3

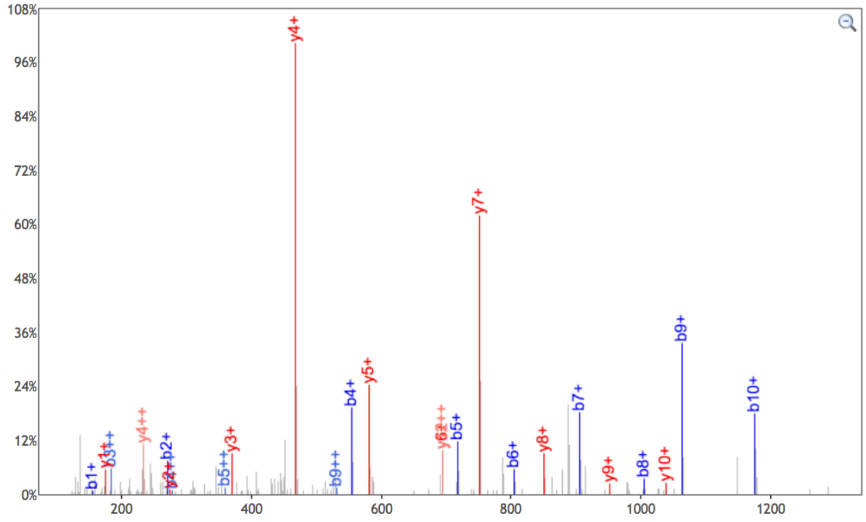

b+	b2+	#	Seq	#	y+	y2+
157.1084	79.0578	1	R	15		
272.1353	136.5713	2	D	14	1599.8530	800.4301
369.1881	185.0977	3	P	13	1484.8260	742.9166
555.2674	278.1373	4	W	12	1387.7732	694.3903
718.3307	359.6690	5	Y	11	1201.6939	601.3506
805.3628	403.1850	6	S	10	1038.6306	519.8189
906.4104	453.7089	7	T	9	951.5986	476.3029
1005.4789	503.2431	8	V	8	850.5509	425.7791
1062.5003	531.7538	9	G	7	751.4825	376.2449
1175.5844	588.2958	10	L	6	694.4610	347.7341
1288.6684	644.8379	11	L	5	581.3770	291.1921
1385.7212	693.3642	12	P	4	468.2929	234.6501
1482.7740	741.8906	13	P	3	371.2401	186.1237
1581.8424	791.4248	14	V	2	274.1874	137.5973
		15	R	1	175.1190	88.0631

[Click] to move table

Static Modifications:
C: 57.02146

Synthetic

RDPWYSTVGLLPPVR, MH+ 1755.9541, m/z 585.9895

File: 20171204_ASJM_24peptides, Scan: 12941, Precursor m/z: 585.9953, Charge: 3

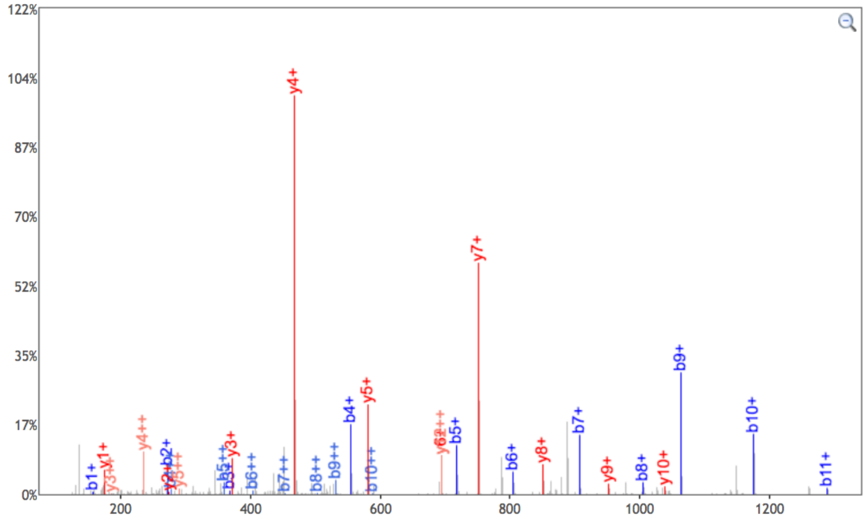

b+	b2+	#	Seq	#	y+	y2+
157.1084	79.0578	1	R	15		
272.1353	136.5713	2	D	14	1599.8530	800.4301
369.1881	185.0977	3	P	13	1484.8260	742.9166
555.2674	278.1373	4	W	12	1387.7732	694.3903
718.3307	359.6690	5	Y	11	1201.6939	601.3506
805.3628	403.1850	6	S	10	1038.6306	519.8189
906.4104	453.7089	7	T	9	951.5986	476.3029
1005.4789	503.2431	8	V	8	850.5509	425.7791
1062.5003	531.7538	9	G	7	751.4825	376.2449
1175.5844	588.2958	10	L	6	694.4610	347.7341
1288.6684	644.8379	11	L	5	581.3770	291.1921
1385.7212	693.3642	12	P	4	468.2929	234.6501
1482.7740	741.8906	13	P	3	371.2401	186.1237
1581.8424	791.4248	14	V	2	274.1874	137.5973
		15	R	1	175.1190	88.0631

[Click] to move table

Static Modifications:
C: 57.02146

Experimental

DLSQQQMQSDLDKADLSAR, MH+ 2149.0190, m/z 717.0112

File: HEK293-acid-highAGC-360minDE15-75um25cm-1, Scan: 16233, Precursor m/z: 717.0156, Charge: 3

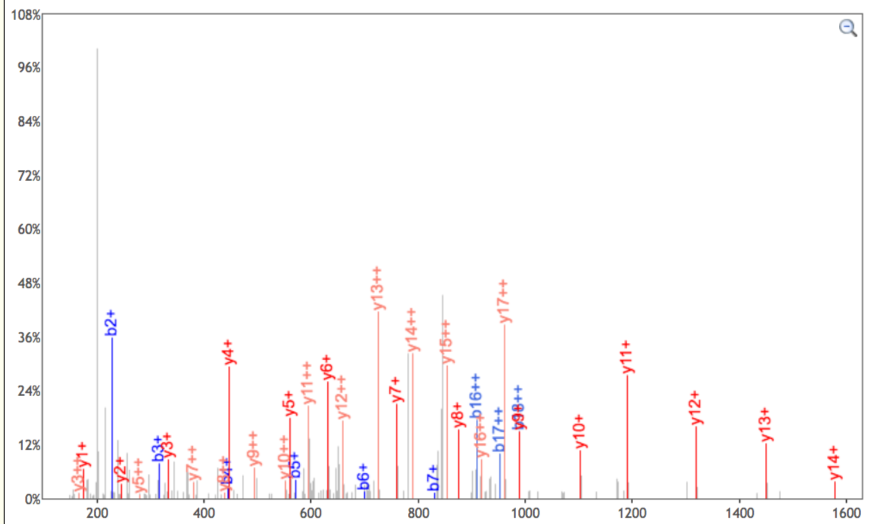

b+	b2+	#	Seq	#	y+	y2+
116.0342	58.5207	1	D	19		
229.1183	115.0628	2	L	18	2033.9920	1017.4997
316.1503	158.5788	3	S	17	1920.9080	960.9576
444.2089	222.6081	4	Q	16	1833.8760	917.4416
572.2675	286.6374	5	Q	15	1705.8174	853.4123
700.3260	350.6667	6	Q	14	1577.7588	789.3830
831.3665	416.1869	7	M	13	1449.7002	725.3537
959.4251	480.2162	8	Q	12	1318.6597	659.8335
1046.4571	523.7322	9	S	11	1190.6012	595.8042
1161.4841	581.2457	10	D	10	1103.5691	552.2882
1274.5681	637.7877	11	L	9	988.5422	494.7747
1389.5951	695.3012	12	D	8	875.4581	438.2327
1517.6900	759.3487	13	K	7	760.4312	380.7192
1588.7272	794.8672	14	A	6	632.3362	316.6717
1703.7541	852.3807	15	D	5	561.2991	281.1532
1816.8382	908.9227	16	L	4	446.2722	223.6397
1903.8702	952.4387	17	S	3	333.1881	167.0977
1974.9073	987.9573	18	A	2	246.1561	123.5817
		19	R	1	175.1190	88.0631

[Click] to move table

Static Modifications:
C: 57.02146

Synthetic

DLSQQQMQSDLDKADLSAR, MH+ 2149.0190, m/z 717.0112

File: 20171204_ASJM_24peptides, Scan: 10230, Precursor m/z: 717.01666, Charge: 3

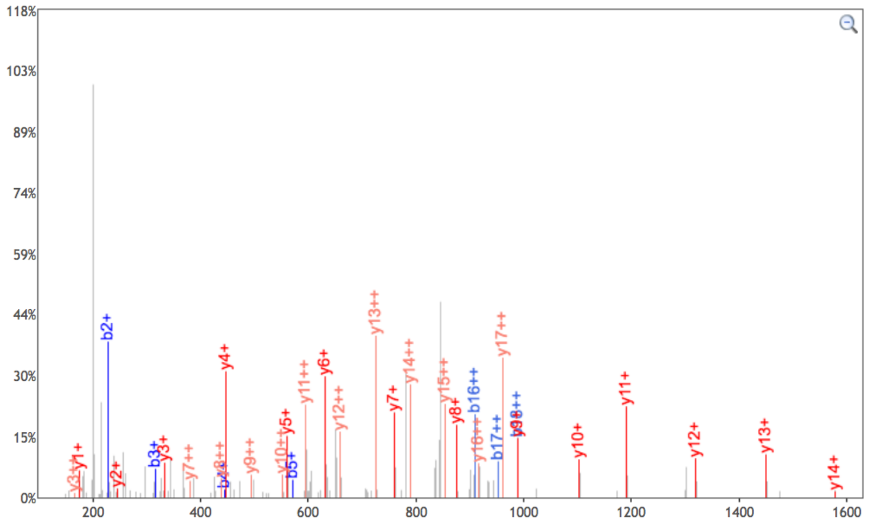

b+	b2+	#	Seq	#	y+	y2+
116.0342	58.5207	1	D	19		
229.1183	115.0628	2	L	18	2033.9920	1017.4997
316.1503	158.5788	3	S	17	1920.9080	960.9576
444.2089	222.6081	4	Q	16	1833.8760	917.4416
572.2675	286.6374	5	Q	15	1705.8174	853.4123
700.3260	350.6667	6	Q	14	1577.7588	789.3830
831.3665	416.1869	7	M	13	1449.7002	725.3537
959.4251	480.2162	8	Q	12	1318.6597	659.8335
1046.4571	523.7322	9	S	11	1190.6012	595.8042
1161.4841	581.2457	10	D	10	1103.5691	552.2882
1274.5681	637.7877	11	L	9	988.5422	494.7747
1389.5951	695.3012	12	D	8	875.4581	438.2327
1517.6900	759.3487	13	K	7	760.4312	380.7192
1588.7272	794.8672	14	A	6	632.3362	316.6717
1703.7541	852.3807	15	D	5	561.2991	281.1532
1816.8382	908.9227	16	L	4	446.2722	223.6397
1903.8702	952.4387	17	S	3	333.1881	167.0977
1974.9073	987.9573	18	A	2	246.1561	123.5817
		19	R	1	175.1190	88.0631

[Click] to move table

Static Modifications:
C: 57.02146

Experimental

DWEGGGGGACHHLPGAR, MH+ 1733.7561, m/z 578.5902
File: HEK293-acid-highAGC-360minDE15-75um25cm-2, Scan: 14378, Precursor m/z: 578.59314, Charge: 3

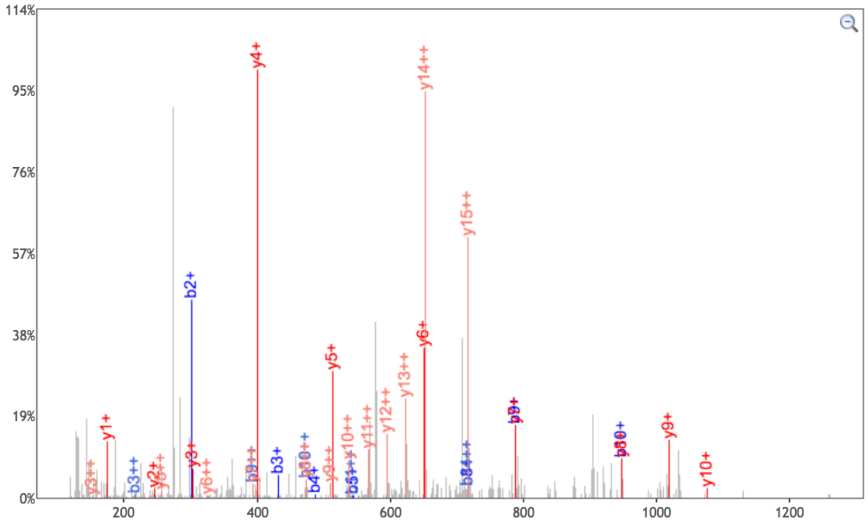

b+	b2+	#	Seq	#	y+	y2+
116.0342	58.5207	1	D	17		
302.1135	151.5604	2	W	16	1618.7292	809.8682
431.1561	216.0817	3	E	15	1432.6499	716.8286
488.1776	244.5924	4	G	14	1303.6073	652.3073
545.1991	273.1032	5	G	13	1246.5858	623.7965
602.2205	301.6139	6	G	12	1189.5643	595.2858
659.2420	330.1246	7	G	11	1132.5429	566.7751
716.2634	358.6354	8	G	10	1075.5214	538.2643
787.3006	394.1539	9	A	9	1018.4999	509.7536
947.3312	474.1692	10	C	8	947.4628	474.2351
1084.3901	542.6987	11	H	7	787.4322	394.2197
1221.4490	611.2282	12	H	6	650.3733	325.6903
1334.5331	667.7702	13	L	5	513.3144	257.1608
1431.5859	716.2966	14	P	4	400.2303	200.6188
1488.6073	744.8073	15	G	3	303.1775	152.0924
1559.6444	780.3259	16	A	2	246.1561	123.5817
		17	R	1	175.1190	88.0631

[Click] to move table

Static Modifications:
C: 57.02146

Synthetic

DWEGGGGGACHHLPGAR, MH+ 1733.7561, m/z 578.5902
File: 20171204_ASJM_24peptides, Scan: 7273, Precursor m/z: 578.59296, Charge: 3

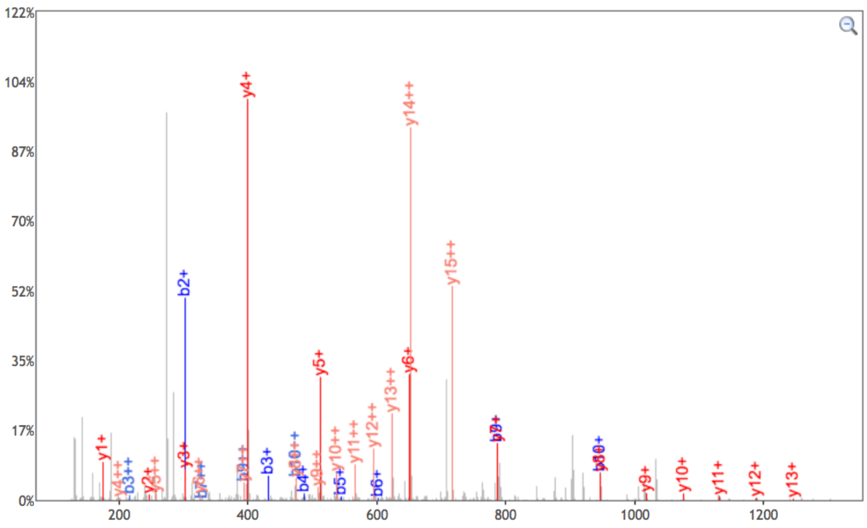

b+	b2+	#	Seq	#	y+	y2+
116.0342	58.5207	1	D	17		
302.1135	151.5604	2	W	16	1618.7292	809.8682
431.1561	216.0817	3	E	15	1432.6499	716.8286
488.1776	244.5924	4	G	14	1303.6073	652.3073
545.1991	273.1032	5	G	13	1246.5858	623.7965
602.2205	301.6139	6	G	12	1189.5643	595.2858
659.2420	330.1246	7	G	11	1132.5429	566.7751
716.2634	358.6354	8	G	10	1075.5214	538.2643
787.3006	394.1539	9	A	9	1018.4999	509.7536
947.3312	474.1692	10	C	8	947.4628	474.2351
1084.3901	542.6987	11	H	7	787.4322	394.2197
1221.4490	611.2282	12	H	6	650.3733	325.6903
1334.5331	667.7702	13	L	5	513.3144	257.1608
1431.5859	716.2966	14	P	4	400.2303	200.6188
1488.6073	744.8073	15	G	3	303.1775	152.0924
1559.6444	780.3259	16	A	2	246.1561	123.5817
		17	R	1	175.1190	88.0631

[Click] to move table

Static Modifications:
C: 57.02146

Experimental

NDDIPEQDSLGLSNLQK, MH+ 1885.9138, m/z 943.4605
File: HEK293-C8-highAGC-360minDE15-75um15cm-1, Scan: 16408, Precursor m/z: 943.4624, Charge: 2

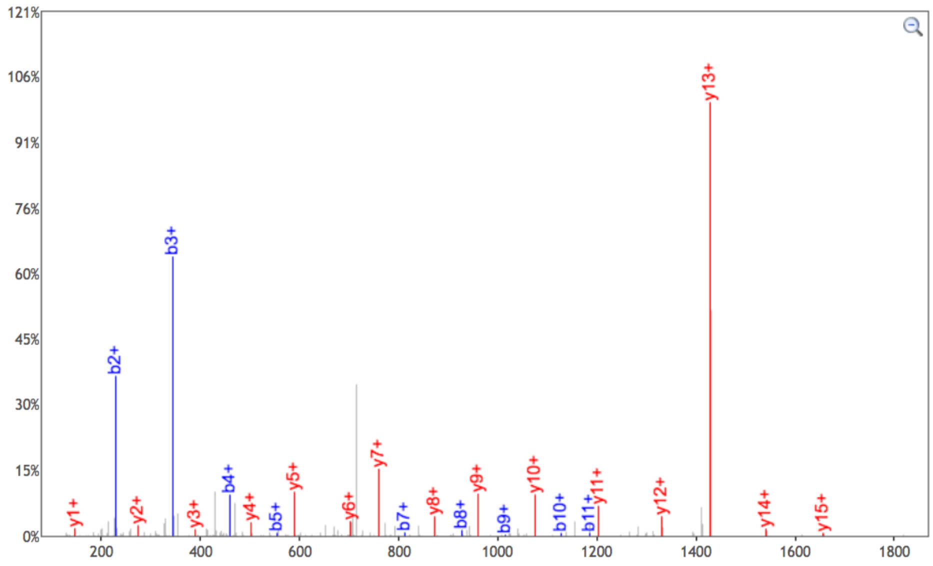

Click and drag in the plot to zoom X: Y: Zoom Out Print Enable tooltip

b+	#	Seq	#	y+
115.0502	1	N	17	
230.0771	2	D	16	1771.8708
345.1041	3	D	15	1656.8439
458.1882	4	I	14	1541.8170
555.2409	5	P	13	1428.7329
684.2835	6	E	12	1331.6801
812.3421	7	Q	11	1202.6375
927.3690	8	D	10	1074.5790
1014.4011	9	S	9	959.5520
1127.4851	10	L	8	872.5200
1184.5066	11	G	7	759.4359
1297.5907	12	L	6	702.4145
1384.6227	13	S	5	589.3304
1498.6656	14	N	4	502.2984
1611.7497	15	L	3	388.2554
1739.8082	16	Q	2	275.1714
	17	K	1	147.1128

[Click] to move table

Static Modifications:
C: 57.02146

Synthetic

NDDIPEQDSLGLSNLQK, MH+ 1885.9138, m/z 943.4605
File: hcd, Scan: 16037, Precursor m/z: 943.4552, Charge: 2

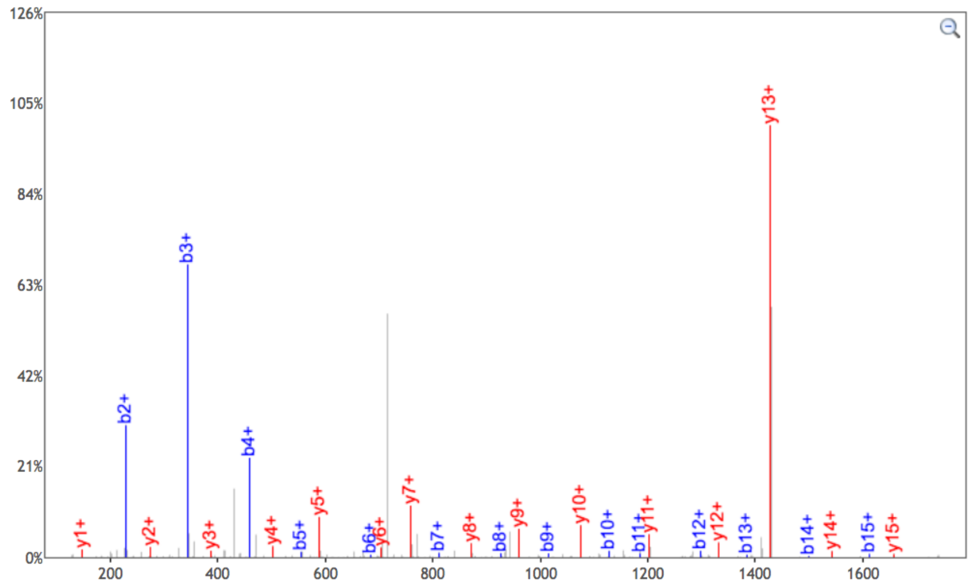

b+	#	Seq	#	y+
115.0502	1	N	17	
230.0771	2	D	16	1771.8708
345.1041	3	D	15	1656.8439
458.1882	4	I	14	1541.8170
555.2409	5	P	13	1428.7329
684.2835	6	E	12	1331.6801
812.3421	7	Q	11	1202.6375
927.3690	8	D	10	1074.5790
1014.4011	9	S	9	959.5520
1127.4851	10	L	8	872.5200
1184.5066	11	G	7	759.4359
1297.5907	12	L	6	702.4145
1384.6227	13	S	5	589.3304
1498.6656	14	N	4	502.2984
1611.7497	15	L	3	388.2554
1739.8082	16	Q	2	275.1714
	17	K	1	147.1128

[Click] to move table

Static Modifications:
C: 57.02146

Experimental

YTDRDFYFASIR, MH+ 1553.7383, m/z 518.5843
File: HEK293-C8-highAGC-360minDE15-75um15cm-1, Scan: 14347, Precursor m/z: 518.58636, Charge: 3

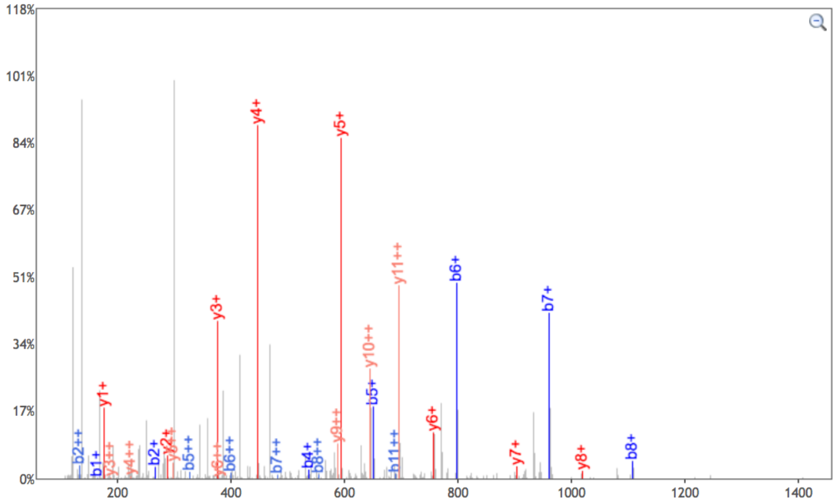

b+	b2+	#	Seq	#	y+	y2+
164.0706	82.5389	1	Y	12		
265.1183	133.0628	2	T	11	1390.6750	695.8411
380.1452	190.5763	3	D	10	1289.6273	645.3173
536.2463	268.6268	4	R	9	1174.6004	587.8038
651.2733	326.1403	5	D	8	1018.4993	509.7533
798.3417	399.6745	6	F	7	903.4723	452.2398
961.4050	481.2061	7	Y	6	756.4039	378.7056
1108.4734	554.7404	8	F	5	593.3406	297.1739
1179.5106	590.2589	9	A	4	446.2722	223.6397
1266.5426	633.7749	10	S	3	375.2350	188.1212
1379.6266	690.3170	11	I	2	288.2030	144.6051
		12	R	1	175.1190	88.0631

[Click] to move table

Static Modifications:
C: 57.02146

Synthetic

YTDRDFYFASIR, MH+ 1553.7383, m/z 518.5843
File: hcd, Scan: 17109, Precursor m/z: 518.58185, Charge: 3

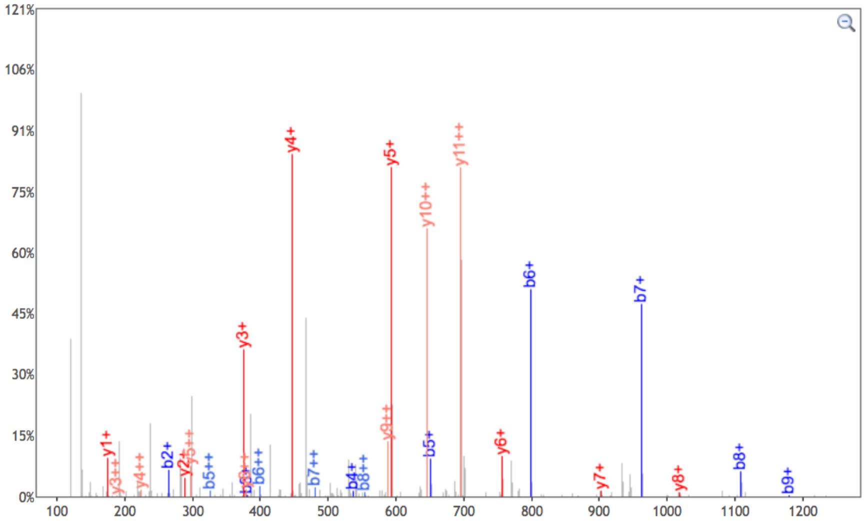

b+	b2+	#	Seq	#	y+	y2+
164.0706	82.5389	1	Y	12		
265.1183	133.0628	2	T	11	1390.6750	695.8411
380.1452	190.5763	3	D	10	1289.6273	645.3173
536.2463	268.6268	4	R	9	1174.6004	587.8038
651.2733	326.1403	5	D	8	1018.4993	509.7533
798.3417	399.6745	6	F	7	903.4723	452.2398
961.4050	481.2061	7	Y	6	756.4039	378.7056
1108.4734	554.7404	8	F	5	593.3406	297.1739
1179.5106	590.2589	9	A	4	446.2722	223.6397
1266.5426	633.7749	10	S	3	375.2350	188.1212
1379.6266	690.3170	11	I	2	288.2030	144.6051
		12	R	1	175.1190	88.0631

[Click] to move table

Static Modifications:
C: 57.02146

Click and drag in the plot to zoom X: ☑ Y: ☐ Zoom Out Print ☐Enable tooltip

Experimental

MQLVQESEEK, MH+ 1220.5827, m/z 610.7950
File: HEK293-C8-highAGC-360minDE15-75um15cm-1, Scan: 3659, Precursor m/z: 610.7976, Charge: 2

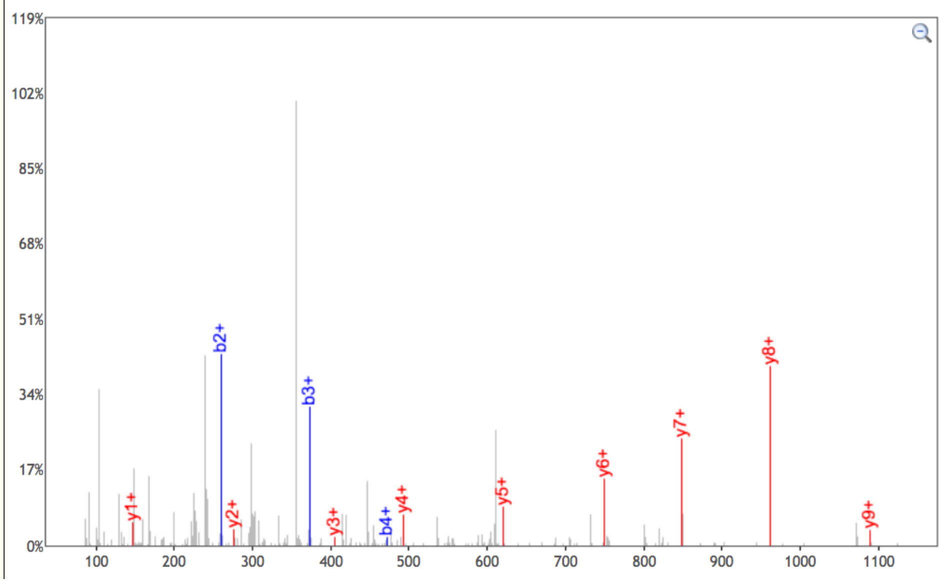

b+	#	Seq	#	y+
132.0478	1	M	10	
260.1063	2	Q	9	1089.5422
373.1904	3	L	8	961.4837
472.2588	4	V	7	848.3996
600.3174	5	Q	6	749.3312
729.3600	6	E	5	621.2726
816.3920	7	S	4	492.2300
945.4346	8	E	3	405.1980
1074.4772	9	E	2	276.1554
	10	K	1	147.1128

[Click] to move table

Static Modifications:
C: 57.02146

Synthetic

MQLVQESEEK, MH+ 1220.5827, m/z 610.7950
File: 20171204_ASJM_24peptides, Scan: 7960, Precursor m/z: 610.7985, Charge: 2

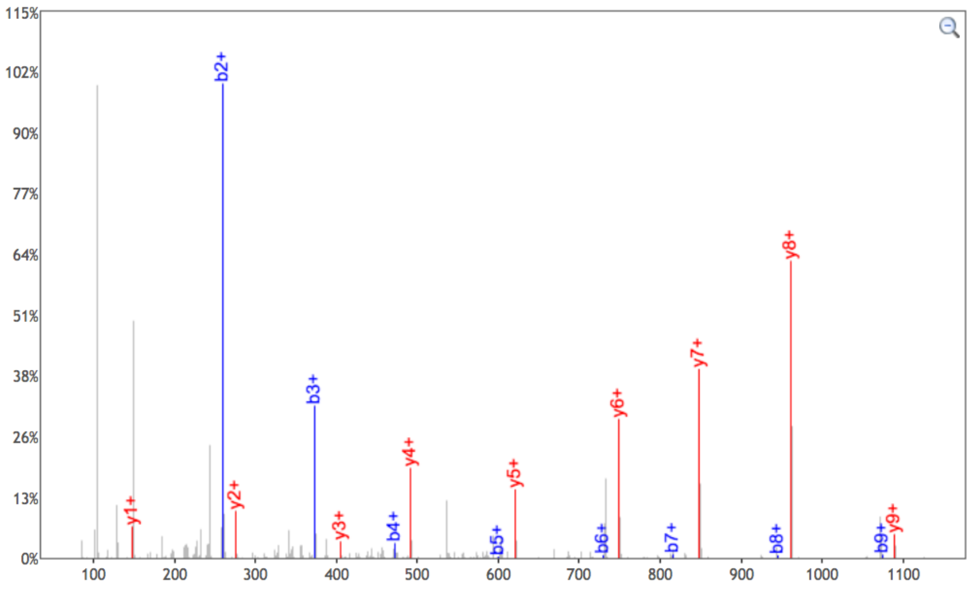

b+	#	Seq	#	y+
132.0478	1	M	10	
260.1063	2	Q	9	1089.5422
373.1904	3	L	8	961.4837
472.2588	4	V	7	848.3996
600.3174	5	Q	6	749.3312
729.3600	6	E	5	621.2726
816.3920	7	S	4	492.2300
945.4346	8	E	3	405.1980
1074.4772	9	E	2	276.1554
	10	K	1	147.1128

[Click] to move table

Static Modifications:
C: 57.02146

Experimental

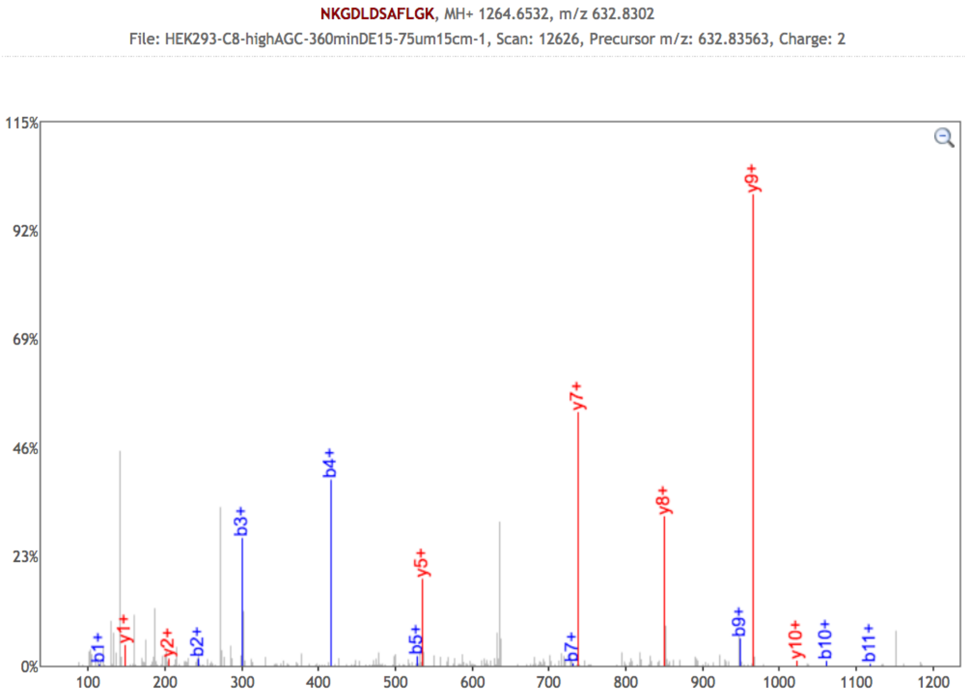

b+	#	Seq	#	y+
115.0502	1	N	12	
243.1452	2	K	11	1150.6103
300.1666	3	G	10	1022.5153
415.1936	4	D	9	965.4938
528.2776	5	L	8	850.4669
643.3046	6	D	7	737.3828
730.3366	7	S	6	622.3559
801.3737	8	A	5	535.3239
948.4421	9	F	4	464.2867
1061.5262	10	L	3	317.2183
1118.5477	11	G	2	204.1343
	12	K	1	147.1128

[Click] to move table

Static Modifications:
C: 57.02146

Synthetic

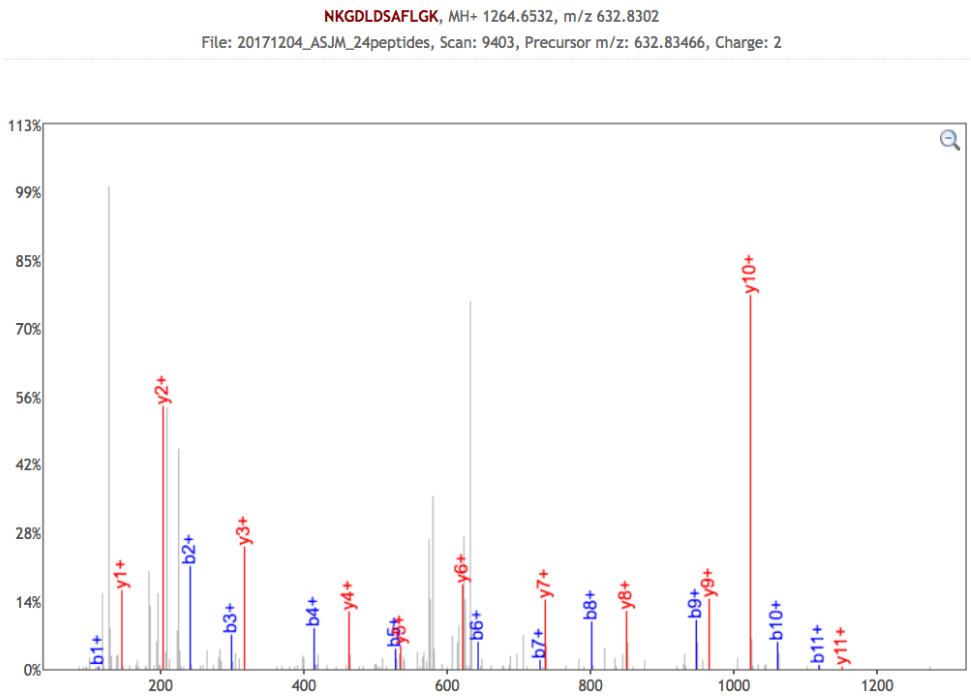

b+	#	Seq	#	y+
115.0502	1	N	12	
243.1452	2	K	11	1150.6103
300.1666	3	G	10	1022.5153
415.1936	4	D	9	965.4938
528.2776	5	L	8	850.4669
643.3046	6	D	7	737.3828
730.3366	7	S	6	622.3559
801.3737	8	A	5	535.3239
948.4421	9	F	4	464.2867
1061.5262	10	L	3	317.2183
1118.5477	11	G	2	204.1343
	12	K	1	147.1128

[Click] to move table

Static Modifications:
C: 57.02146

Experimental

NSTLSEPGSGR, MH+ 1104.5280, m/z 552.7676
File: HEK293-acid-highAGC-360minDE15-75um25cm-2, Scan: 6180, Precursor m/z: 552.773, Charge: 2

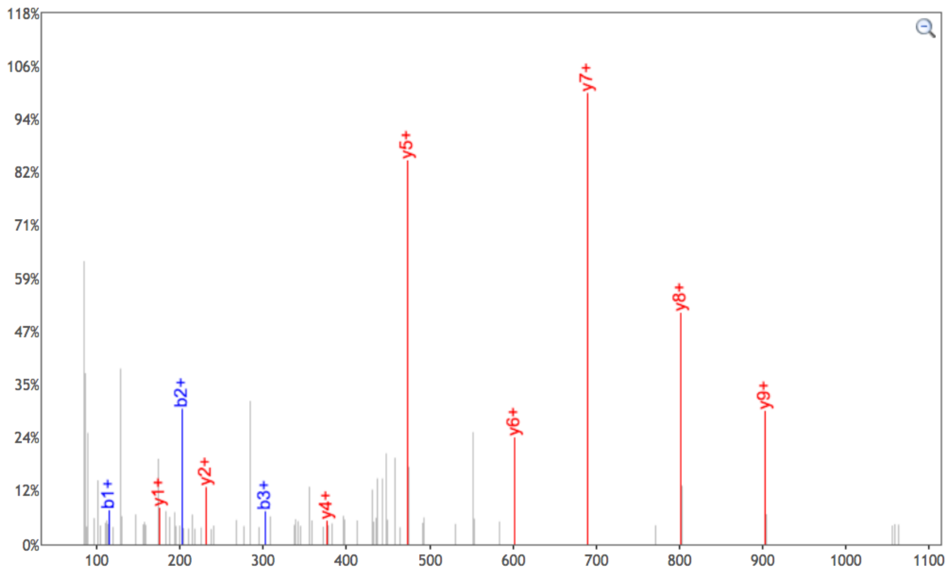

b+	#	Seq	#	y+
115.0502	1	N	11	
202.0822	2	S	10	990.4851
303.1299	3	T	9	903.4530
416.2140	4	L	8	802.4054
503.2460	5	S	7	689.3213
632.2886	6	E	6	602.2893
729.3414	7	P	5	473.2467
786.3628	8	G	4	376.1939
873.3949	9	S	3	319.1724
930.4163	10	G	2	232.1404
	11	R	1	175.1190

[Click] to move table

Static Modifications:
C: 57.02146

Synthetic

NSTLSEPGSGR, MH+ 1104.5280, m/z 552.7676
File: 20171204_ASJM_24peptides, Scan: 5498, Precursor m/z: 552.7697, Charge: 2

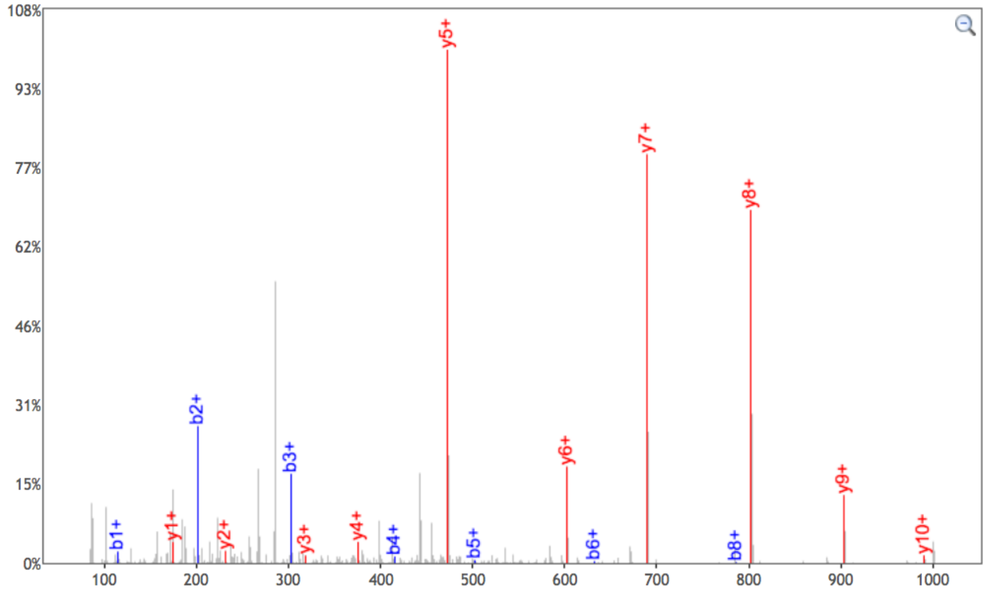

b+	#	Seq	#	y+
115.0502	1	N	11	
202.0822	2	S	10	990.4851
303.1299	3	T	9	903.4530
416.2140	4	L	8	802.4054
503.2460	5	S	7	689.3213
632.2886	6	E	6	602.2893
729.3414	7	P	5	473.2467
786.3628	8	G	4	376.1939
873.3949	9	S	3	319.1724
930.4163	10	G	2	232.1404
	11	R	1	175.1190

[Click] to move table

Static Modifications:
C: 57.02146

Experimental

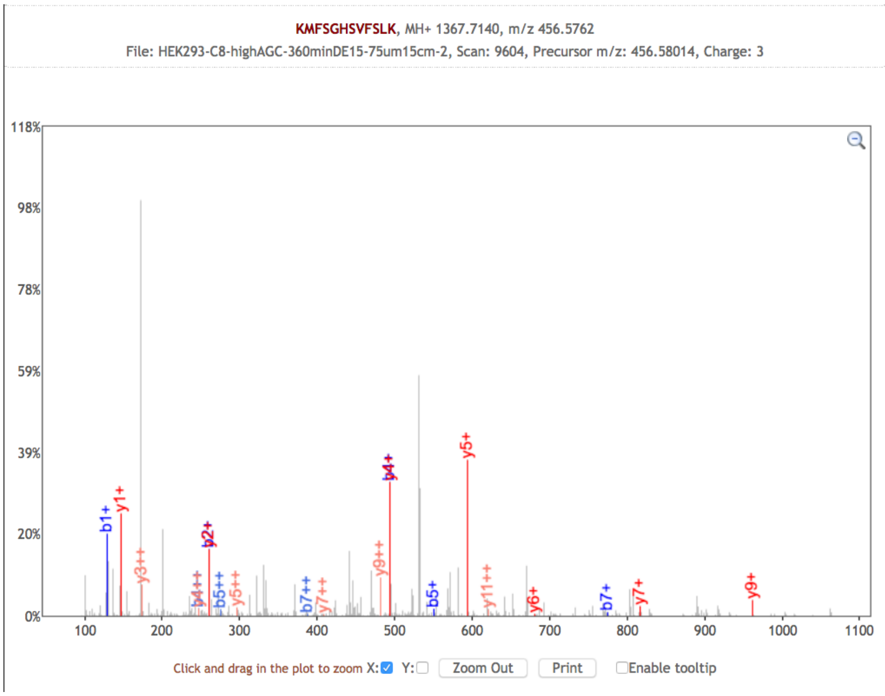

b+	b2+	#	Seq	#	y+	y2+
129.1022	65.0548	1	K	12		
260.1427	130.5750	2	M	11	1239.6191	620.3132
407.2111	204.1092	3	F	10	1108.5786	554.7929
494.2432	247.6252	4	S	9	961.5102	481.2587
551.2646	276.1360	5	G	8	874.4781	437.7427
688.3235	344.6654	6	H	7	817.4567	409.2320
775.3556	388.1814	7	S	6	680.3978	340.7025
874.4240	437.7156	8	V	5	593.3657	297.1865
1021.4924	511.2498	9	F	4	494.2973	247.6523
1108.5244	554.7659	10	S	3	347.2289	174.1181
1221.6085	611.3079	11	L	2	260.1969	130.6021
		12	K	1	147.1128	74.0600

[Click] to move table

Static Modifications:
C: 57.02146

Synthetic

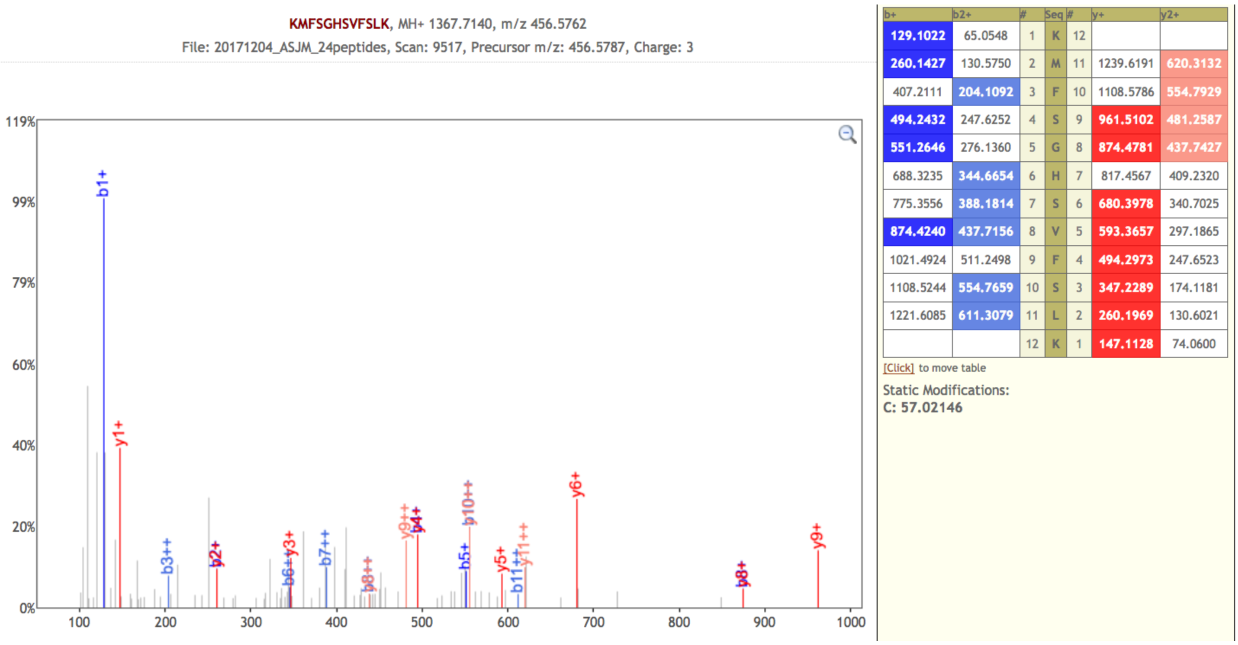

b+	b2+	#	Seq	#	y+	y2+
129.1022	65.0548	1	K	12		
260.1427	130.5750	2	M	11	1239.6191	620.3132
407.2111	204.1092	3	F	10	1108.5786	554.7929
494.2432	247.6252	4	S	9	961.5102	481.2587
551.2646	276.1360	5	G	8	874.4781	437.7427
688.3235	344.6654	6	H	7	817.4567	409.2320
775.3556	388.1814	7	S	6	680.3978	340.7025
874.4240	437.7156	8	V	5	593.3657	297.1865
1021.4924	511.2498	9	F	4	494.2973	247.6523
1108.5244	554.7659	10	S	3	347.2289	174.1181
1221.6085	611.3079	11	L	2	260.1969	130.6021
		12	K	1	147.1128	74.0600

[Click] to move table

Static Modifications:
C: 57.02146

Experimental

TTLLAQSVCAPVR, MH+ 1415.7675, m/z 708.3874

File: HEK293-acid-highAGC-360minDE15-75um25cm-1, Scan: 26727, Precursor m/z: 708.382, Charge: 2

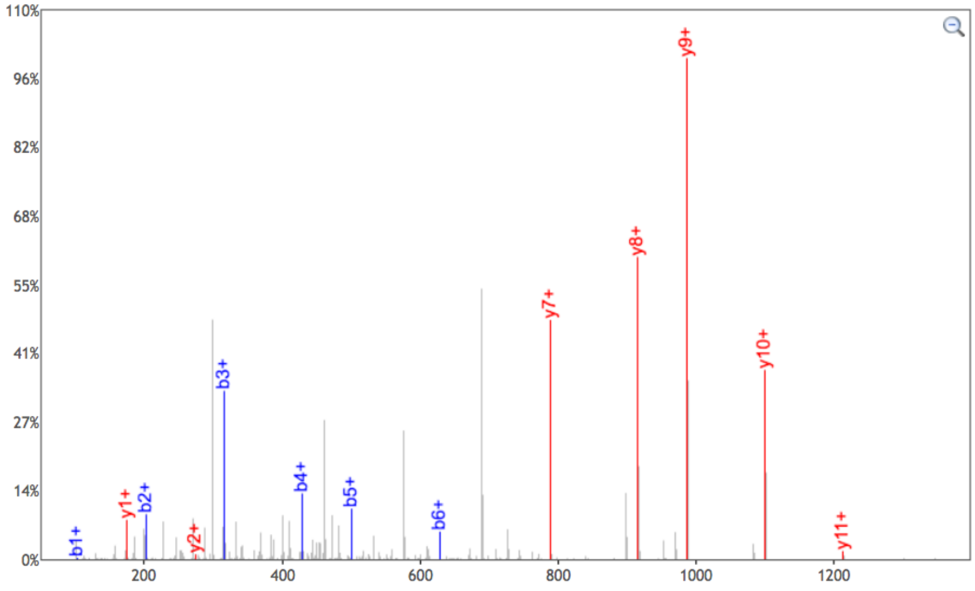

Click and drag in the plot to zoom X: Y: Zoom Out Print Enable tooltip

b+	#	Seq	#	y+
102.0550	1	T	13	
203.1026	2	T	12	1314.7198
316.1867	3	L	11	1213.6721
429.2708	4	L	10	1100.5881
500.3079	5	A	9	987.5040
628.3665	6	Q	8	916.4669
715.3985	7	S	7	788.4083
814.4669	8	V	6	701.3763
974.4975	9	C	5	602.3079
1045.5347	10	A	4	442.2772
1142.5874	11	P	3	371.2401
1241.6558	12	V	2	274.1874
	13	R	1	175.1190

[Click] to move table

Static Modifications:
C: 57.02146

Synthetic

TTLLAQSVCAPVR, MH+ 1415.7675, m/z 708.3874

File: 20171204_ASJM_24peptides, Scan: 9810, Precursor m/z: 708.3909, Charge: 2

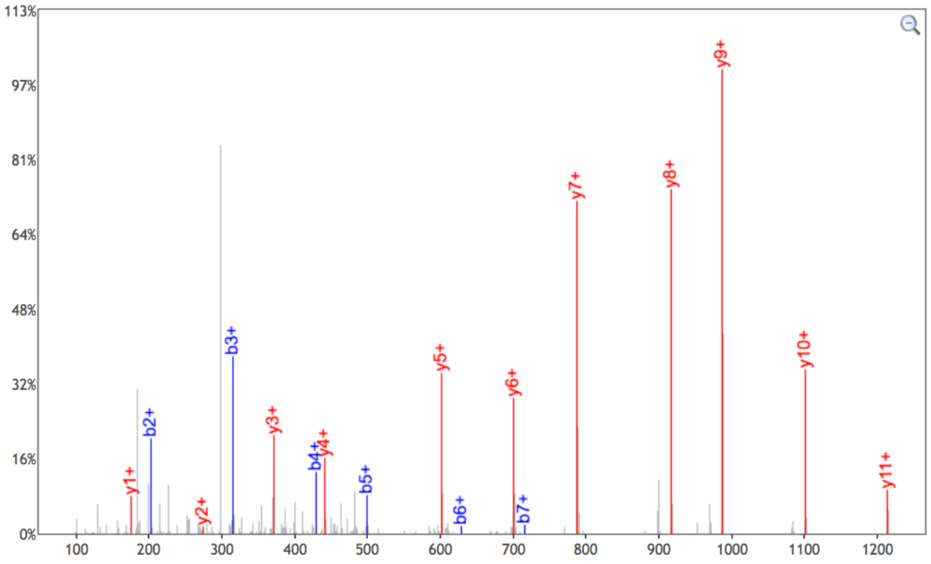

b+	#	Seq	#	y+
102.0550	1	T	13	
203.1026	2	T	12	1314.7198
316.1867	3	L	11	1213.6721
429.2708	4	L	10	1100.5881
500.3079	5	A	9	987.5040
628.3665	6	Q	8	916.4669
715.3985	7	S	7	788.4083
814.4669	8	V	6	701.3763
974.4975	9	C	5	602.3079
1045.5347	10	A	4	442.2772
1142.5874	11	P	3	371.2401
1241.6558	12	V	2	274.1874
	13	R	1	175.1190

[Click] to move table

Static Modifications:
C: 57.02146

Experimental

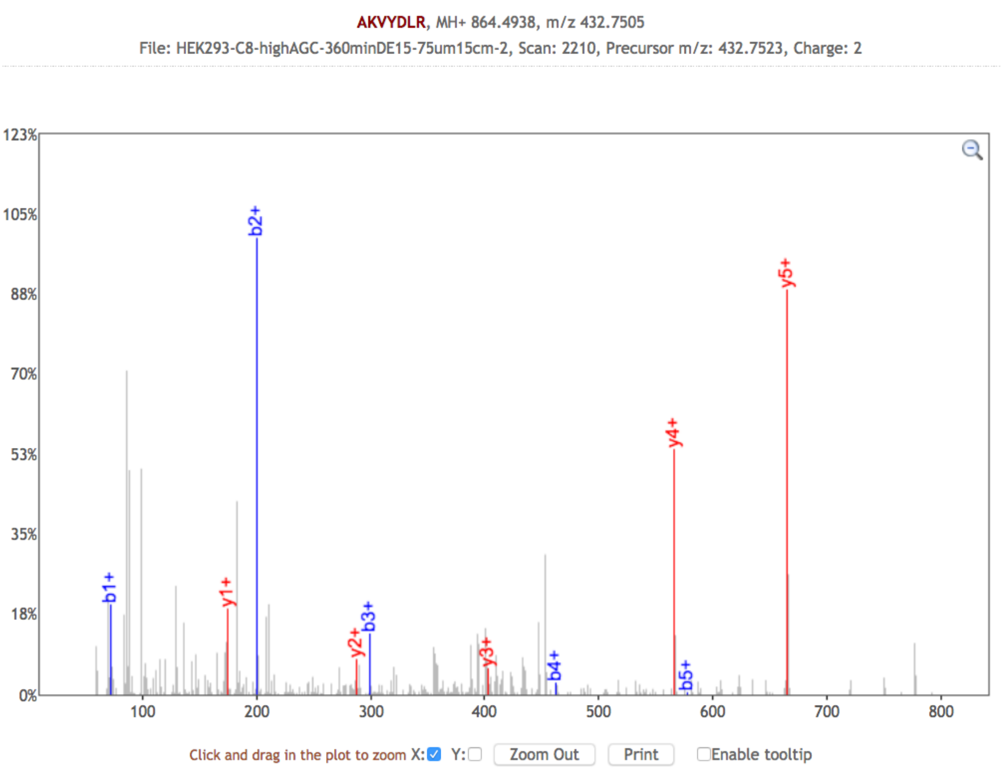

b+	#	Seq	#	y+
72.0444	1	A	7	
200.1394	2	K	6	793.4567
299.2078	3	V	5	665.3617
462.2711	4	Y	4	566.2933
577.2980	5	D	3	403.2300
690.3821	6	L	2	288.2030
	7	R	1	175.1190

[Click] to move table

Static Modifications:
C: 57.02146

Synthetic

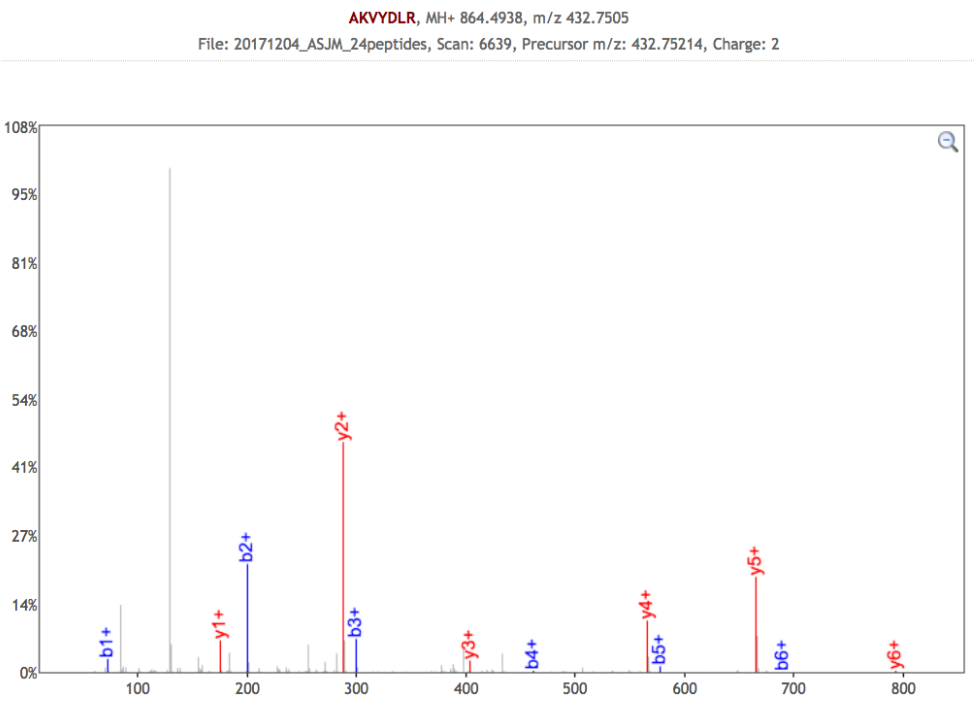

b+	#	Seq	#	y+
72.0444	1	A	7	
200.1394	2	K	6	793.4567
299.2078	3	V	5	665.3617
462.2711	4	Y	4	566.2933
577.2980	5	D	3	403.2300
690.3821	6	L	2	288.2030
	7	R	1	175.1190

[Click] to move table

Static Modifications:
C: 57.02146

QQQNSNIFFLADRTEMLSESK, MH+ 2486.1980, m/z 829.4042

File: HEK293-acid-highAGC-360minDE15-75um25cm-1, Scan: 23174, Precursor m/z: 829.4085, Charge: 3

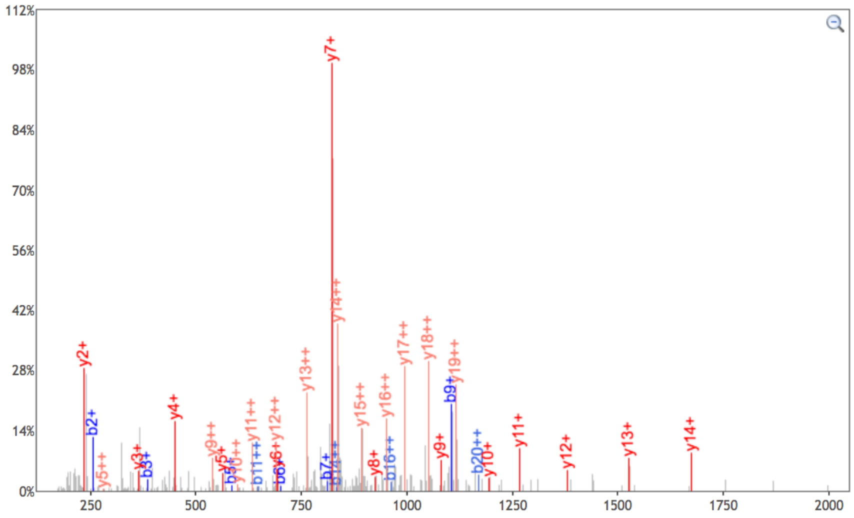

Click and drag in the plot to zoom X: Y: Zoom Out Print Enable tooltip

b+	b2+	#	Seq	#	y+	y2+
129.0659	65.0366	1	Q	21		
257.1244	129.0659	2	Q	20	2358.1394	1179.5734
385.1830	193.0951	3	Q	19	2230.0809	1115.5441
499.2259	250.1166	4	N	18	2102.0223	1051.5148
586.2580	293.6326	5	S	17	1987.9794	994.4933
700.3009	350.6541	6	N	16	1900.9473	950.9773
813.3850	407.1961	7	I	15	1786.9044	893.9558
960.4534	480.7303	8	F	14	1673.8203	837.4138
1107.5218	554.2645	9	F	13	1526.7519	763.8796
1220.6058	610.8066	10	L	12	1379.6835	690.3454
1291.6430	646.3251	11	A	11	1266.5994	633.8034
1406.6699	703.8386	12	D	10	1195.5623	598.2848
1562.7710	781.8891	13	R	9	1080.5354	540.7713
1663.8187	832.4130	14	T	8	924.4343	462.7208
1792.8613	896.9343	15	E	7	823.3866	412.1969
1923.9018	962.4545	16	M	6	694.3440	347.6756
2036.9858	1018.9966	17	L	5	563.3035	282.1554
2124.0179	1062.5126	18	S	4	450.2195	225.6134
2253.0605	1127.0339	19	E	3	363.1874	182.0974
2340.0925	1170.5499	20	S	2	234.1448	117.5761
		21	K	1	147.1128	74.0600

[Click] to move table

Static Modifications:
C: 57.02146

NILDELKKEYQEIENLDK, MH+ 2234.1551, m/z 559.2942

File: HEK293-acid-highAGC-360minDE15-75um25cm-2, Scan: 33196, Precursor m/z: 559.29834, Charge: 4

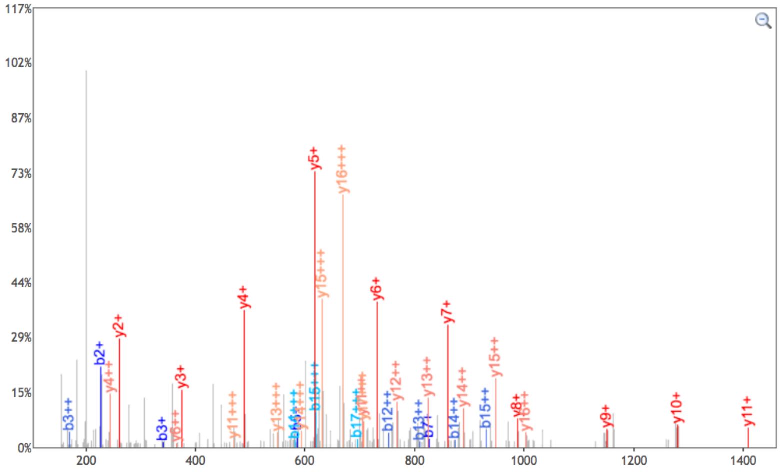

Click and drag in the plot to zoom X: Y: Zoom Out Print Enable tooltip

b+	b2+	b3+	#	Seq	#	y+	y2+	y3+
115.0502	58.0287	39.0216	1	N	18			
228.1343	114.5708	76.7163	2	I	17	2120.1121	1060.5597	707.3756
341.2183	171.1128	114.4110	3	L	16	2007.0281	1004.0177	669.6809
456.2453	228.6263	152.7533	4	D	15	1893.9440	947.4756	631.9862
585.2879	293.1476	195.7675	5	E	14	1778.9171	889.9622	593.6439
698.3719	349.6896	233.4622	6	L	13	1649.8745	825.4409	550.6297
826.4669	413.7371	276.1605	7	K	12	1536.7904	768.8988	512.9350
954.5619	477.7846	318.8588	8	K	11	1408.6955	704.8514	470.2367
1083.6045	542.3059	361.8730	9	E	10	1280.6005	640.8039	427.5383
1246.6678	623.8375	416.2274	10	Y	9	1151.5579	576.2826	384.5241
1374.7264	687.8668	458.9136	11	Q	8	988.4946	494.7509	330.1697
1503.7690	752.3881	501.9278	12	E	7	860.4360	430.7216	287.4835
1616.8530	808.9301	539.6225	13	I	6	731.3934	366.2003	244.4693
1745.8956	873.4514	582.6367	14	E	5	618.3093	309.6583	206.7746
1859.9385	930.4729	620.6510	15	N	4	489.2667	245.1370	163.7604
1973.0226	987.0149	658.3457	16	L	3	375.2238	188.1155	125.7461
2088.0495	1044.5284	696.6880	17	D	2	262.1397	131.5735	88.0514
			18	K	1	147.1128	74.0600	49.7091

[Click] to move table

Static Modifications:
C: 57.02146

IVVDELSNLK, MH+ 1129.6463, m/z 565.3268

File: HEK293-acid-highAGC-360minDE15-75um25cm-2, Scan: 20194, Precursor m/z: 565.3301, Charge: 2

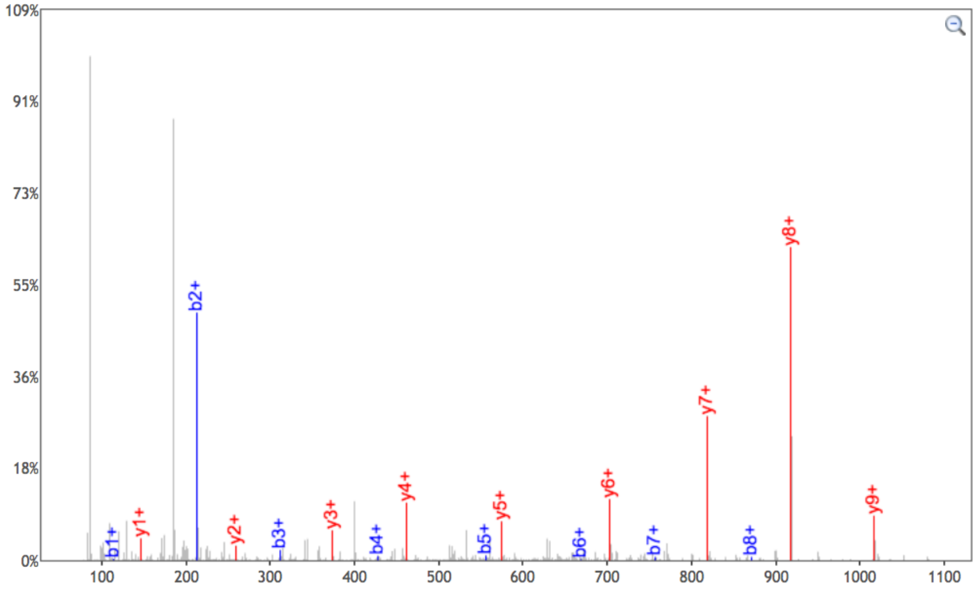

Click and drag in the plot to zoom X: Y: Zoom Out Print Enable tooltip

b+	#	Seq	#	y+
114.0913	1	I	10	
213.1598	2	V	9	1016.5623
312.2282	3	V	8	917.4938
427.2551	4	D	7	818.4254
556.2977	5	E	6	703.3985
669.3818	6	L	5	574.3559
756.4138	7	S	4	461.2718
870.4567	8	N	3	374.2398
983.5408	9	L	2	260.1969
	10	K	1	147.1128

[Click] to move table

Static Modifications:
C: 57.02146

EYQEIENLDK, MH+ 1280.6005, m/z 640.8039

File: HEK293-acid-highAGC-360minDE15-75um25cm-1, Scan: 9631, Precursor m/z: 640.80756, Charge: 2

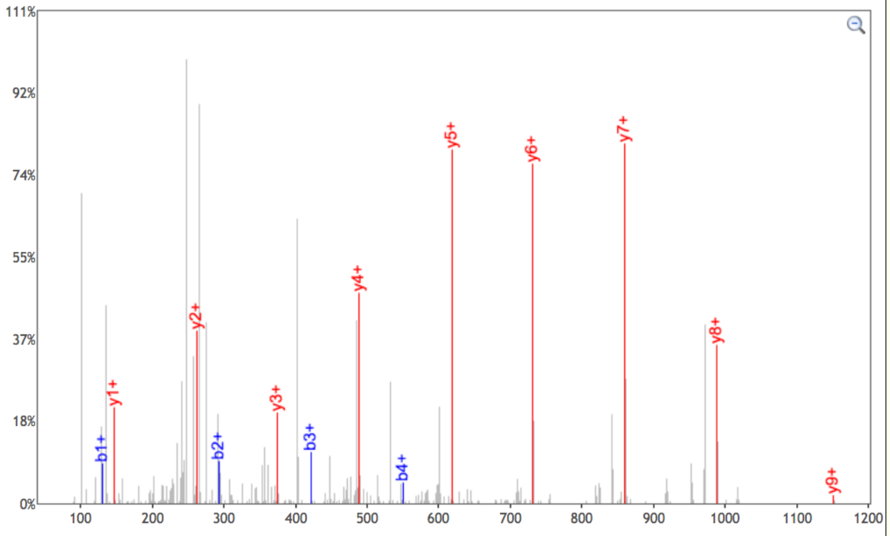

Click and drag in the plot to zoom X: Y: Zoom Out Print Enable tooltip

b+	#	Seq	#	y+
130.0499	1	E	10	
293.1132	2	Y	9	1151.5579
421.1718	3	Q	8	988.4946
550.2144	4	E	7	860.4360
663.2984	5	I	6	731.3934
792.3410	6	E	5	618.3093
906.3840	7	N	4	489.2667
1019.4680	8	L	3	375.2238
1134.4950	9	D	2	262.1397
	10	K	1	147.1128

[Click] to move table

Static Modifications:
C: 57.02146

EYQEIENLDKTK, MH+ 1509.7431, m/z 503.9192
File: HEK293-acid-highAGC-360minDE15-75um25cm-1, Scan: 7852, Precursor m/z: 503.92233, Charge: 3

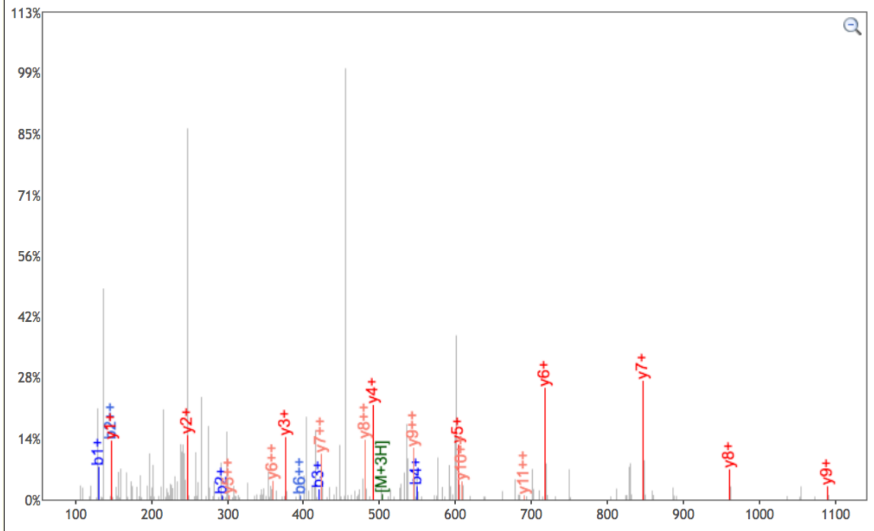

Click and drag in the plot to zoom X: Y: Zoom Out Print Enable tooltip

b+	b2+	#	Seq	#	y+	y2+
130.0499	65.5286	1	E	12		
293.1132	147.0602	2	Y	11	1380.7005	690.8539
421.1718	211.0895	3	Q	10	1217.6372	609.3222
550.2144	275.6108	4	E	9	1089.5786	545.2930
663.2984	332.1529	5	I	8	960.5360	480.7717
792.3410	396.6742	6	E	7	847.4520	424.2296
906.3840	453.6956	7	N	6	718.4094	359.7083
1019.4680	510.2376	8	L	5	604.3665	302.6869
1134.4950	567.7511	9	D	4	491.2824	246.1448
1262.5899	631.7986	10	K	3	376.2554	188.6314
1363.6376	682.3224	11	T	2	248.1605	124.5839
		12	K	1	147.1128	74.0600

[Click] to move table

Static Modifications:
C: 57.02146

RNLEGRMINCFVLWWLHDCTFVK, MH+ 2994.4688, m/z 998.8278
File: HEK293-acid-highAGC-360minDE15-75um25cm-3_150123234949, Scan: 31046, Precursor m/z: 998.8354, Charge: 3

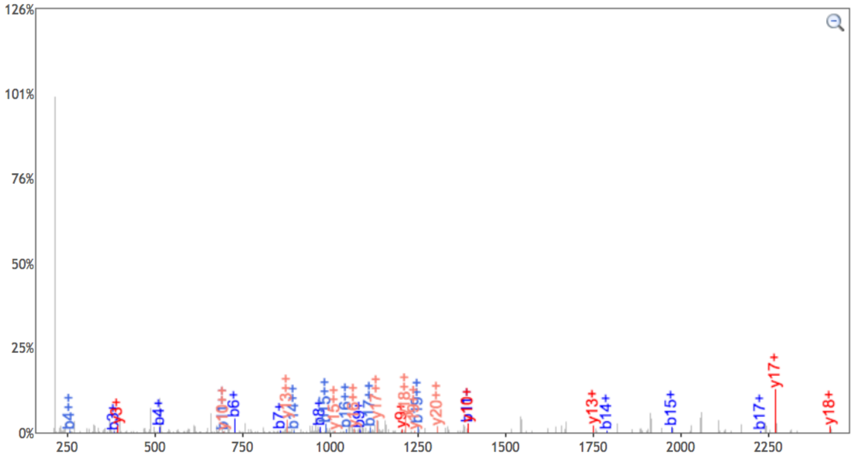

Click and drag in the plot to zoom X: Y: Zoom Out Print Enable tooltip

b+	b2+	#	Seq	#	y+	y2+
157.1084	79.0578	1	R	23		
271.1513	136.0793	2	N	22	2838.3677	1419.6875
384.2354	192.6213	3	L	21	2724.3247	1362.6660
513.2780	257.1426	4	E	20	2611.2407	1306.1240
570.2994	285.6534	5	G	19	2482.1981	1241.6027
726.4005	363.7039	6	R	18	2425.1766	1213.0919
857.4410	429.2242	7	M	17	2269.0755	1135.0414
970.5251	485.7662	8	I	16	2138.0350	1069.5212
1084.5680	542.7876	9	N	15	2024.9510	1012.9791
1244.5987	622.8030	10	C	14	1910.9080	955.9577
1391.6671	696.3372	11	F	13	1750.8774	875.9423
1490.7355	745.8714	12	V	12	1603.8090	802.4081
1603.8196	802.4134	13	L	11	1504.7406	752.8739
1789.8989	895.4531	14	W	10	1391.6565	696.3319
1975.9782	988.4927	15	W	9	1205.5772	603.2922
2089.0622	1045.0348	16	L	8	1019.4979	510.2526
2226.1212	1113.5642	17	H	7	906.4138	453.7105
2341.1481	1171.0777	18	D	6	769.3549	385.1811
2501.1787	1251.0930	19	C	5	654.3280	327.6676
2602.2264	1301.6169	20	T	4	494.2973	247.6523
2749.2948	1375.1511	21	F	3	393.2496	197.1285
2848.3633	1424.6853	22	V	2	246.1812	123.5942
		23	K	1	147.1128	74.0600

[Click] to move table

Static Modifications:

GPLSHAAPNWTPDPAAAVAAGGTSLPPRGGGGI, MH+ 3020.5337, m/z 504.2617

File: HEK293-acid-highAGC-360minDE15-75um25cm-1, Scan: 6121, Precursor m/z: 504.2577, Charge: 6

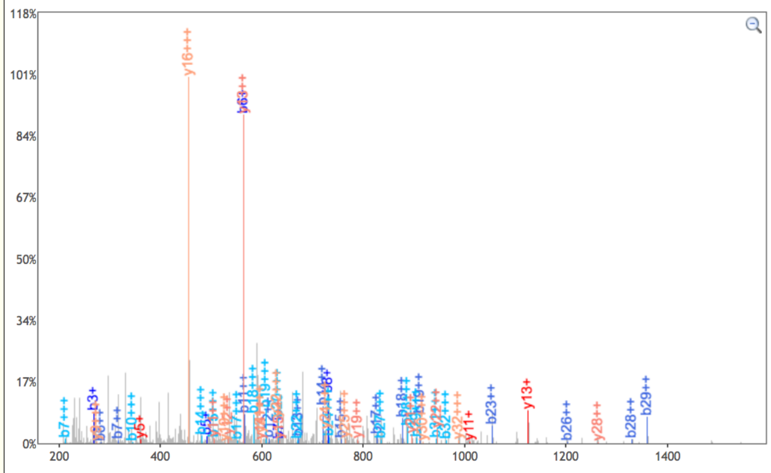

Click and drag in the plot to zoom X: Y: Zoom Out Print Enable tooltip

b+	b2+	b3+	#	Seq	#	y+	y2+	y3+
58.0287	29.5180	20.0144	1	G	33			
155.0815	78.0444	52.3654	2	P	32	2963.5122	1482.2598	988.5089
268.1656	134.5864	90.0600	3	L	31	2866.4595	1433.7334	956.1580
355.1976	178.1024	119.0707	4	S	30	2753.3754	1377.1913	918.4633
492.2565	246.6319	164.7570	5	H	29	2666.3434	1333.6753	889.4526
563.2936	282.1504	188.4361	6	A	28	2529.2845	1265.1459	843.7663
634.3307	317.6690	212.1151	7	A	27	2458.2473	1229.6273	820.0873
731.3835	366.1954	244.4660	8	P	26	2387.2102	1194.1088	796.4083
845.4264	423.2169	282.4803	9	N	25	2290.1575	1145.5824	764.0573
1031.5057	516.2565	344.5068	10	W	24	2176.1145	1088.5609	726.0430
1132.5534	566.7803	378.1893	11	T	23	1990.0352	995.5213	664.0166
1229.6062	615.3067	410.5402	12	P	22	1888.9876	944.9974	630.3340
1344.6331	672.8202	448.8826	13	D	21	1791.9348	896.4710	597.9831
1441.6859	721.3466	481.2335	14	P	20	1676.9078	838.9576	559.6408
1512.7230	756.8651	504.9125	15	A	19	1579.8551	790.4312	527.2899
1583.7601	792.3837	528.5916	16	A	18	1508.8180	754.9126	503.6108
1654.7972	827.9023	552.2706	17	A	17	1437.7809	719.3941	479.9318
1753.8656	877.4365	585.2934	18	V	16	1366.7437	683.8755	456.2528
1824.9028	912.9550	608.9724	19	A	15	1267.6753	634.3413	423.2300
1895.9399	948.4736	632.6515	20	A	14	1196.6382	598.8227	399.5509
1952.9613	976.9843	651.6586	21	G	13	1125.6011	563.3042	375.8719
2009.9828	1005.4950	670.6658	22	G	12	1068.5796	534.7935	356.8647
2111.0305	1056.0189	704.3483	23	T	11	1011.5582	506.2827	337.8576
2198.0625	1099.5349	733.3590	24	S	10	910.5105	455.7589	304.1750
2311.1466	1156.0769	771.0537	25	L	9	823.4785	412.2429	275.1643
2408.1993	1204.6033	803.4046	26	P	8	710.3944	355.7008	237.4697
2505.2521	1253.1297	835.7556	27	P	7	613.3416	307.1745	205.1187
2661.3532	1331.1802	887.7893	28	R	6	516.2889	258.6481	172.7678
2718.3747	1359.6910	906.7964	29	G	5	360.1878	180.5975	120.7341
2775.3961	1388.2017	925.8036	30	G	4	303.1663	152.0868	101.7269
2832.4176	1416.7124	944.8107	31	G	3	246.1448	123.5761	82.7198
2889.4391	1445.2232	963.8179	32	G	2	189.1234	95.0653	63.7126
			33	I	1	132.1019	66.5546	44.7055

[Click] to move table

Static Modifications:
C: 57.02146

EDPHVTHLQVAQDTVTPEAAQISSEEHPQEK, MH+ 3450.6408, m/z 690.9340

File: HEK293-acid-highAGC-360minDE15-75um25cm-1, Scan: 14053, Precursor m/z: 690.9396, Charge: 5

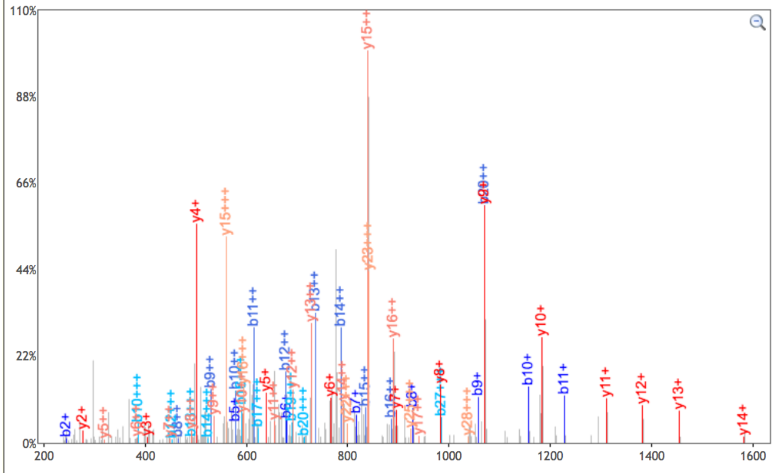

Click and drag in the plot to zoom X: Y: Zoom Out Print Enable tooltip

b+	b2+	b3+	#	Seq	#	y+	y2+	y3+
130.0499	65.5286	44.0215	1	E	31			
245.0768	123.0420	82.3638	2	D	30	3321.5982	1661.3027	1107.8709
342.1296	171.5684	114.7147	3	P	29	3206.5713	1603.7893	1069.5286
479.1885	240.0979	160.4010	4	H	28	3109.5185	1555.2629	1037.1777
578.2569	289.6321	193.4238	5	V	27	2972.4596	1486.7334	991.4914
679.3046	340.1559	227.1064	6	T	26	2873.3912	1437.1992	958.4686
816.3635	408.6854	272.7927	7	H	25	2772.3435	1386.6754	924.7860
929.4476	465.2274	310.4874	8	L	24	2635.2846	1318.1459	879.0997
1057.5061	529.2567	353.1736	9	Q	23	2522.2005	1261.6039	841.4050
1156.5745	578.7909	386.1964	10	V	22	2394.1419	1197.5746	798.7188
1227.6117	614.3095	409.8754	11	A	21	2295.0735	1148.0404	765.6960
1355.6702	678.3388	452.5616	12	Q	20	2224.0364	1112.5218	742.0170
1470.6972	735.8522	490.9039	13	D	19	2095.9778	1048.4926	699.3308
1571.7449	786.3761	524.5865	14	T	18	1980.9509	990.9791	660.9885
1670.8133	835.9103	557.6093	15	V	17	1879.9032	940.4552	627.3059
1771.8610	886.4341	591.2918	16	T	16	1780.8348	890.9210	594.2831
1868.9137	934.9605	623.6428	17	P	15	1679.7871	840.3972	560.6006
1997.9563	999.4818	666.6570	18	E	14	1582.7344	791.8708	528.2496
2068.9934	1035.0003	690.3360	19	A	13	1453.6918	727.3495	485.2354
2140.0305	1070.5189	714.0150	20	A	12	1382.6546	691.8310	461.5564
2268.0891	1134.5482	756.7012	21	Q	11	1311.6175	656.3124	437.8774
2381.1732	1191.0902	794.3959	22	I	10	1183.5590	592.2831	395.1912
2468.2052	1234.6062	823.4066	23	S	9	1070.4749	535.7411	357.4965
2555.2372	1278.1223	852.4173	24	S	8	983.4429	492.2251	328.4858
2684.2798	1342.6436	895.4315	25	E	7	896.4108	448.7091	299.4751
2813.3224	1407.1648	938.4457	26	E	6	767.3682	384.1878	256.4609
2950.3813	1475.6943	984.1320	27	H	5	638.3257	319.6665	213.4467
3047.4341	1524.2207	1016.4829	28	P	4	501.2667	251.1370	167.7604
3175.4927	1588.2500	1059.1691	29	Q	3	404.2140	202.6106	135.4095
3304.5353	1652.7713	1102.1833	30	E	2	276.1554	138.5813	92.7233
			31	K	1	147.1128	74.0600	49.7091

[Click] to move table

Static Modifications:
C: 57.02146

WDYPEGTPNGGSTTLPSAPPPASAGLK, MH+ 2668.2889, m/z 890.1012
File: HEK293-acid-highAGC-360minDE15-75um25cm-1, Scan: 21369, Precursor m/z: 890.1086, Charge: 3

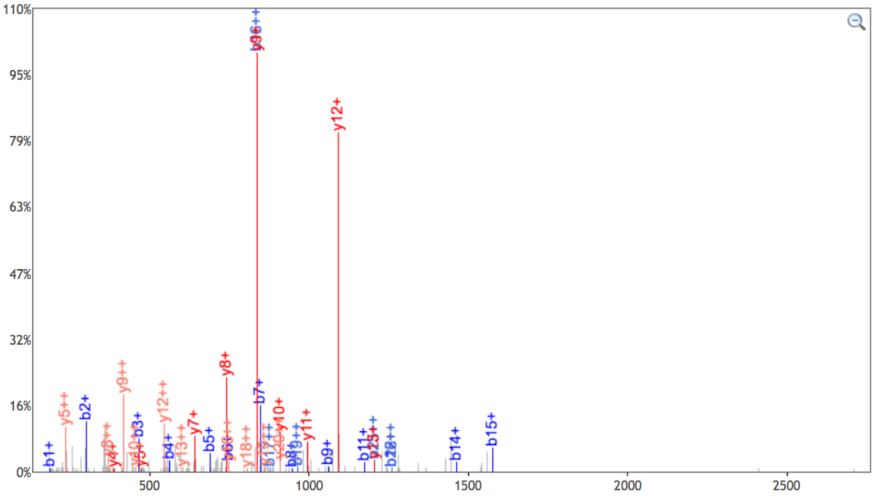

Click and drag in the plot to zoom X: Y: Zoom Out Print Enable tooltip

b+	b2+	#	Seq	#	y+	y2+
187.0866	94.0469	1	W	27		
302.1135	151.5604	2	D	26	2482.2096	1241.6085
465.1769	233.0921	3	Y	25	2367.1827	1184.0950
562.2296	281.6185	4	P	24	2204.1194	1102.5633
691.2722	346.1397	5	E	23	2107.0666	1054.0369
748.2937	374.6505	6	G	22	1978.0240	989.5156
849.3414	425.1743	7	T	21	1921.0025	961.0049
946.3941	473.7007	8	P	20	1819.9549	910.4811
1060.4371	530.7222	9	N	19	1722.9021	861.9547
1117.4585	559.2329	10	G	18	1608.8592	804.9332
1174.4800	587.7436	11	G	17	1551.8377	776.4225
1261.5120	631.2596	12	S	16	1494.8162	747.9118
1362.5597	681.7835	13	T	15	1407.7842	704.3957
1463.6074	732.3073	14	T	14	1306.7365	653.8719
1576.6914	788.8494	15	L	13	1205.6888	603.3481
1673.7442	837.3757	16	P	12	1092.6048	546.8060
1760.7762	880.8917	17	S	11	995.5520	498.2796
1831.8133	916.4103	18	A	10	908.5200	454.7636
1928.8661	964.9367	19	P	9	837.4829	419.2451
2025.9189	1013.4631	20	P	8	740.4301	370.7187
2122.9716	1061.9895	21	P	7	643.3774	322.1923
2194.0087	1097.5080	22	A	6	546.3246	273.6659
2281.0408	1141.0240	23	S	5	475.2875	238.1474
2352.0779	1176.5426	24	A	4	388.2554	194.6314
2409.0993	1205.0533	25	G	3	317.2183	159.1128
2522.1834	1261.5953	26	L	2	260.1969	130.6021
		27	K	1	147.1128	74.0600

[Click] to move table

Static Modifications:
C: 57.02146

SGHEDTGNPGLGFLFHELQEGDNAK, MH+ 2669.2226, m/z 668.0611
File: HEK293-acid-highAGC-360minDE15-75um25cm-2, Scan: 26164, Precursor m/z: 668.06525, Charge: 4

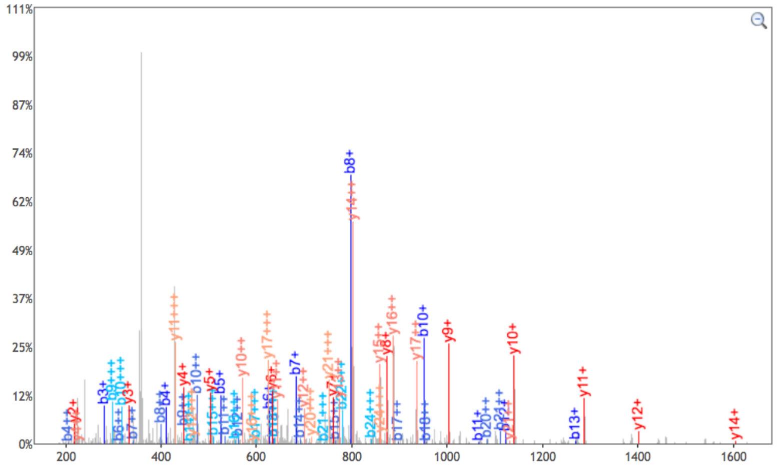

Click and drag in the plot to zoom X: Y: Zoom Out Print Enable tooltip

b+	b2+	b3+	#	Seq	#	y+	y2+	y3+
88.0393	44.5233	30.0180	1	S	25			
145.0608	73.0340	49.0251	2	G	24	2582.1906	1291.5989	861.4017
282.1197	141.5635	94.7114	3	H	23	2525.1692	1263.0882	842.3946
411.1623	206.0848	137.7256	4	E	22	2388.1102	1194.5588	796.7083
526.1892	263.5982	176.0679	5	D	21	2259.0677	1130.0375	753.6941
627.2369	314.1221	209.7505	6	T	20	2144.0407	1072.5240	715.3518
684.2584	342.6328	228.7576	7	G	19	2042.9930	1022.0002	681.6692
798.3013	399.6543	266.7719	8	N	18	1985.9716	993.4894	662.6620
895.3540	448.1807	299.1229	9	P	17	1871.9286	936.4680	624.6477
952.3755	476.6914	318.1300	10	G	16	1774.8759	887.9416	592.2968
1065.4596	533.2334	355.8247	11	L	15	1717.8544	859.4308	573.2897
1122.4810	561.7442	374.8319	12	G	14	1604.7703	802.8888	535.5950
1269.5495	635.2784	423.8547	13	F	13	1547.7489	774.3781	516.5878
1382.6335	691.8204	461.5494	14	L	12	1400.6805	700.8439	467.5650
1529.7019	765.3546	510.5722	15	F	11	1287.5964	644.3018	429.8703
1666.7608	833.8841	556.2585	16	H	10	1140.5280	570.7676	380.8475
1795.8034	898.4054	599.2727	17	E	9	1003.4691	502.2382	335.1612
1908.8875	954.9474	636.9674	18	L	8	874.4265	437.7169	292.1470
2036.9461	1018.9767	679.6535	19	Q	7	761.3424	381.1748	254.4523
2165.9887	1083.4980	722.6677	20	E	6	633.2838	317.1456	211.7661
2223.0101	1112.0087	741.6749	21	G	5	504.2413	252.6243	168.7519
2338.0371	1169.5222	780.0172	22	D	4	447.2198	224.1135	149.7448
2452.0800	1226.5436	818.0315	23	N	3	332.1928	166.6001	111.4025
2523.1171	1262.0622	841.7106	24	A	2	218.1499	109.5786	73.3882
			25	K	1	147.1128	74.0600	49.7091

[Click] to move table

Static Modifications:
C: 57.02146

AVGTLVTEAGGDEEK, MH+ 1475.7224, m/z 738.3648

File: HEK293-acid-highAGC-360minDE15-75um25cm-1, Scan: 8677, Precursor m/z: 738.3697, Charge: 2

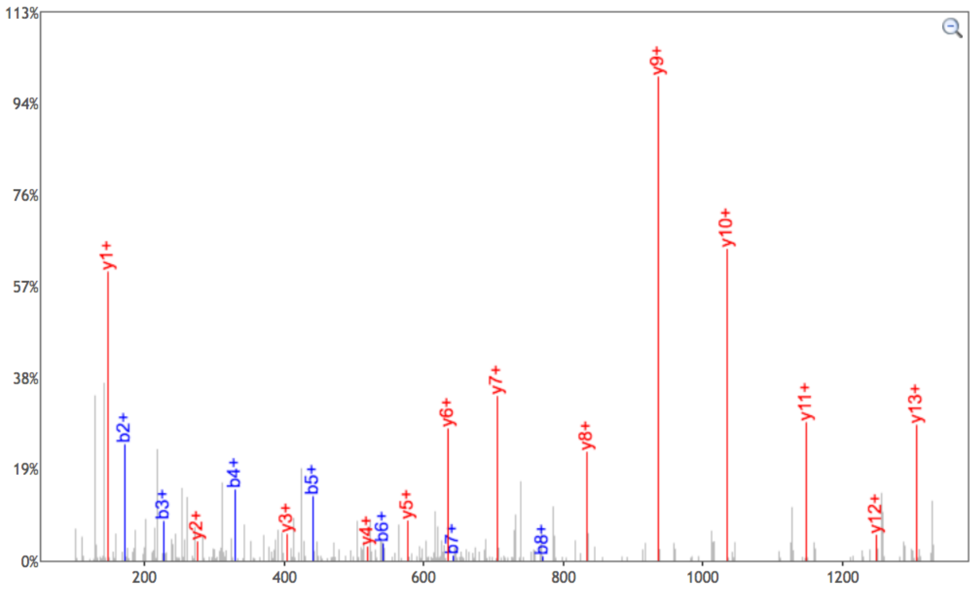

Click and drag in the plot to zoom X: Y: Zoom Out Print Enable tooltip

b+	#	Seq	#	y+
72.0444	1	A	15	
171.1128	2	V	14	1404.6853
228.1343	3	G	13	1305.6169
329.1819	4	T	12	1248.5954
442.2660	5	L	11	1147.5477
541.3344	6	V	10	1034.4637
642.3821	7	T	9	935.3952
771.4247	8	E	8	834.3476
842.4618	9	A	7	705.3050
899.4833	10	G	6	634.2679
956.5047	11	G	5	577.2464
1071.5317	12	D	4	520.2249
1200.5743	13	E	3	405.1980
1329.6169	14	E	2	276.1554
	15	K	1	147.1128

[Click] to move table

Static Modifications:
C: 57.02146

GGATTPILAASLQPQCVQQVAPEGAR, MH+ 2620.3512, m/z 874.1219

File: HEK293-acid-highAGC-360minDE15-75um25cm-1, Scan: 22436, Precursor m/z: 874.1259, Charge: 3

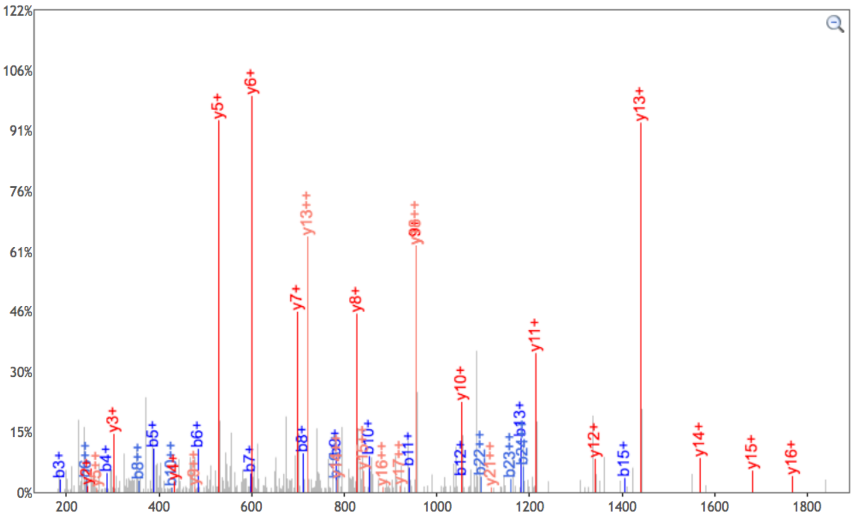

Click and drag in the plot to zoom X: Y: Zoom Out Print Enable tooltip

b+	b2+	#	Seq	#	y+	y2+
58.0287	29.5180	1	G	26		
115.0502	58.0287	2	G	25	2563.3297	1282.1685
186.0873	93.5473	3	A	24	2506.3082	1253.6578
287.1350	144.0711	4	T	23	2435.2711	1218.1392
388.1827	194.5950	5	T	22	2334.2234	1167.6154
485.2354	243.1214	6	P	21	2233.1758	1117.0915
598.3195	299.6634	7	I	20	2136.1230	1068.5651
711.4036	356.2054	8	L	19	2023.0389	1012.0231
782.4407	391.7240	9	A	18	1909.9549	955.4811
853.4778	427.2425	10	A	17	1838.9178	919.9625
940.5098	470.7585	11	S	16	1767.8806	884.4440
1053.5939	527.3006	12	L	15	1680.8486	840.9279
1181.6525	591.3299	13	Q	14	1567.7645	784.3859
1278.7052	639.8563	14	P	13	1439.7060	720.3566
1406.7638	703.8855	15	Q	12	1342.6532	671.8302
1566.7944	783.9009	16	C	11	1214.5946	607.8010
1665.8629	833.4351	17	V	10	1054.5640	527.7856
1793.9214	897.4644	18	Q	9	955.4956	478.2514
1921.9800	961.4936	19	Q	8	827.4370	414.2221
2021.0484	1011.0279	20	V	7	699.3784	350.1928
2092.0855	1046.5464	21	A	6	600.3100	300.6586
2189.1383	1095.0728	22	P	5	529.2729	265.1401
2318.1809	1159.5941	23	E	4	432.2201	216.6137
2375.2024	1188.1048	24	G	3	303.1775	152.0924
2446.2395	1223.6234	25	A	2	246.1561	123.5817
		26	R	1	175.1190	88.0631

[Click] to move table

Static Modifications:
C: 57.02146

PVTGATTTSRSSCWLQVRR, MH+ 2163.1087, m/z 1082.0580

File: HEK293-acid-highAGC-360minDE15-75um25cm-1, Scan: 13022, Precursor m/z: 1082.067, Charge: 2

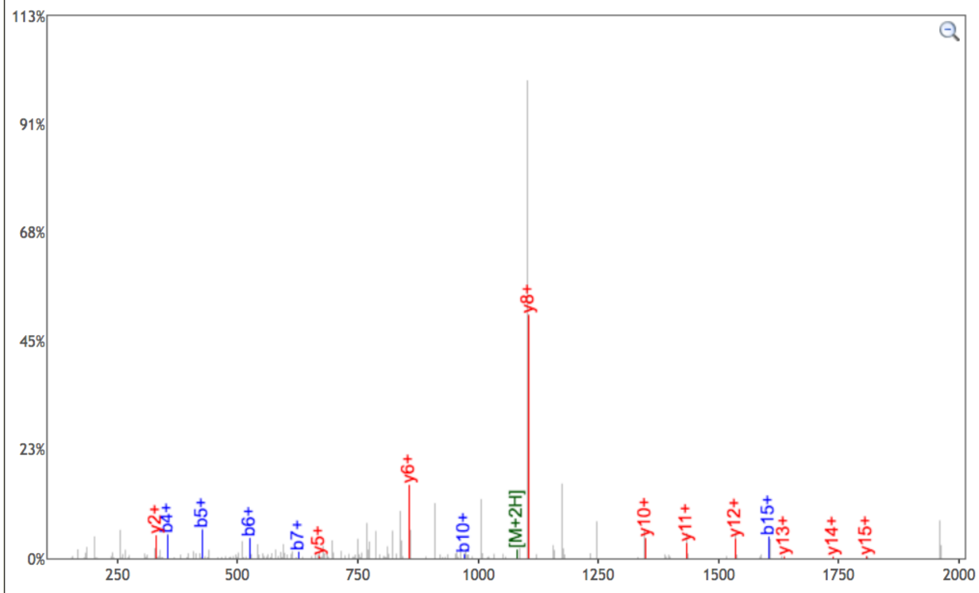

Click and drag in the plot to zoom X: ☑ Y: ☐ Zoom Out Print ☐Enable tooltip

b+	#	Seq	#	y+
98.0600	1	P	19	
197.1285	2	V	18	2066.0560
298.1761	3	T	17	1966.9876
355.1976	4	G	16	1865.9399
426.2347	5	A	15	1808.9184
527.2824	6	T	14	1737.8813
628.3301	7	T	13	1636.8336
729.3777	8	T	12	1535.7860
816.4098	9	S	11	1434.7383
972.5109	10	R	10	1347.7062
1059.5429	11	S	9	1191.6051
1146.5749	12	S	8	1104.5731
1306.6056	13	C	7	1017.5411
1492.6849	14	W	6	857.5104
1605.7690	15	L	5	671.4311
1733.8275	16	Q	4	558.3471
1832.8960	17	V	3	430.2885
1988.9971	18	R	2	331.2201
	19	R	1	175.1190

[Click] to move table

Static Modifications:
C: 57.02146

DPWYSTVGLLPPVR, MH+ 1599.8530, m/z 800.4301

File: HEK293-acid-highAGC-360minDE15-75um25cm-1, Scan: 28579, Precursor m/z: 800.4368, Charge: 2

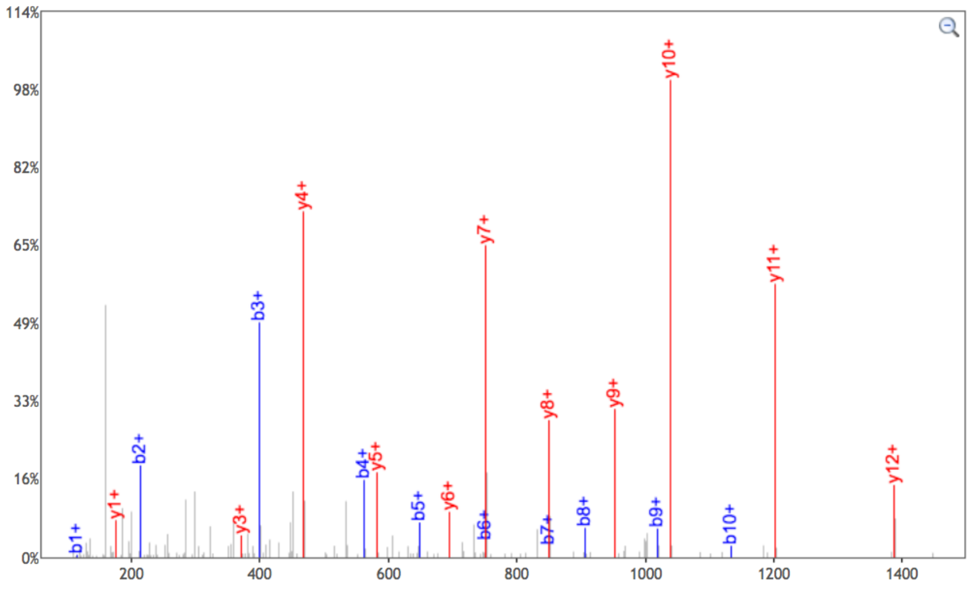

Click and drag in the plot to zoom X: ☑ Y: ☐ Zoom Out Print ☐Enable tooltip

b+	#	Seq	#	y+
116.0342	1	D	14	
213.0870	2	P	13	1484.8260
399.1663	3	W	12	1387.7732
562.2296	4	Y	11	1201.6939
649.2617	5	S	10	1038.6306
750.3093	6	T	9	951.5986
849.3777	7	V	8	850.5509
906.3992	8	G	7	751.4825
1019.4833	9	L	6	694.4610
1132.5673	10	L	5	581.3770
1229.6201	11	P	4	468.2929
1326.6729	12	P	3	371.2401
1425.7413	13	V	2	274.1874
	14	R	1	175.1190

[Click] to move table

Static Modifications:
C: 57.02146

TEQGPTGVTMTSNPITWGQIK, MH+ 2246.1122, m/z 1123.5597
File: HEK293-acid-highAGC-360minDE15-75um25cm-2, Scan: 25231, Precursor m/z: 1123.5707, Charge: 2

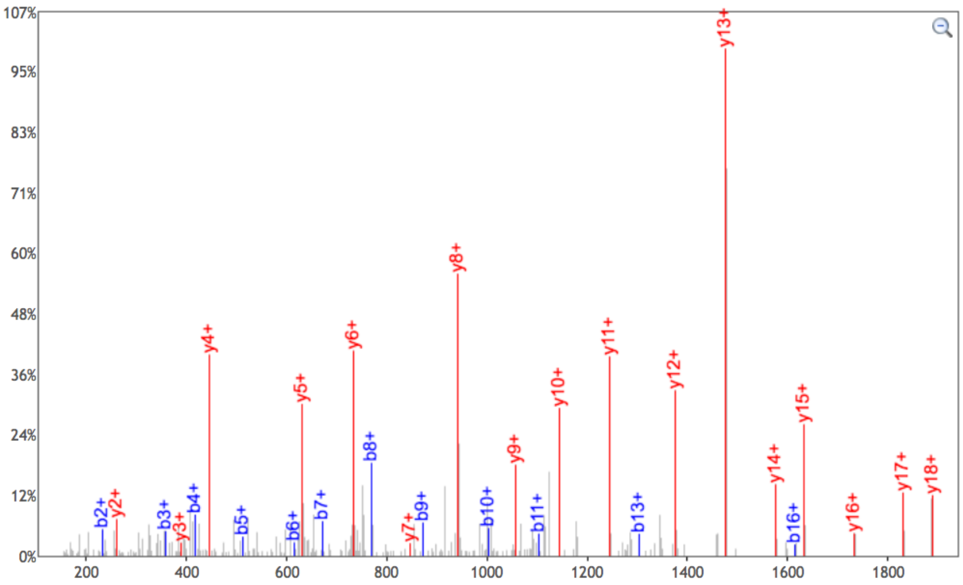

Click and drag in the plot to zoom X: ☑ Y: ☐ Zoom Out Print ☐Enable tooltip

b+	#	Seq	#	y+
102.0550	1	T	21	
231.0975	2	E	20	2145.0645
359.1561	3	Q	19	2016.0219
416.1776	4	G	18	1887.9633
513.2304	5	P	17	1830.9418
614.2780	6	T	16	1733.8891
671.2995	7	G	15	1632.8414
770.3679	8	V	14	1575.8199
871.4156	9	T	13	1476.7515
1002.4561	10	M	12	1375.7038
1103.5038	11	T	11	1244.6634
1190.5358	12	S	10	1143.6157
1304.5787	13	N	9	1056.5837
1401.6315	14	P	8	942.5407
1514.7155	15	I	7	845.4880
1615.7632	16	T	6	732.4039
1801.8425	17	W	5	631.3562
1858.8640	18	G	4	445.2769
1986.9226	19	Q	3	388.2554
2100.0066	20	I	2	260.1969
	21	K	1	147.1128

[Click] to move table

Static Modifications:
C: 57.02146

VGCLSPGLARVSQIHCAAK, MH+ 2024.0528, m/z 1012.5300
File: HEK293-acid-highAGC-360minDE15-75um25cm-1, Scan: 16550, Precursor m/z: 1012.5377, Charge: 2

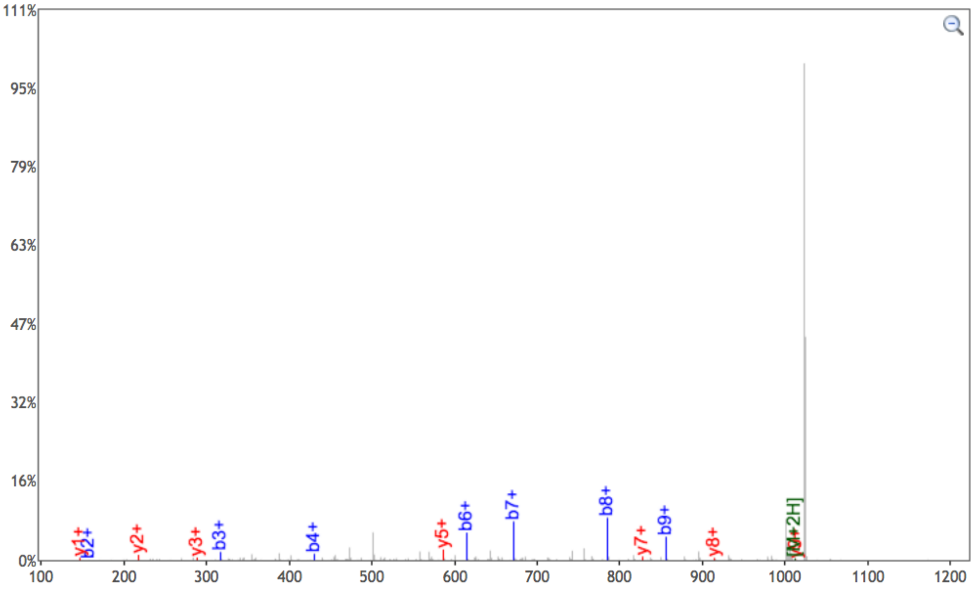

Click and drag in the plot to zoom X: ☑ Y: ☐ Zoom Out Print ☐Enable tooltip

b+	#	Seq	#	y+
100.0757	1	V	19	
157.0972	2	G	18	1924.9844
317.1278	3	C	17	1867.9629
430.2119	4	L	16	1707.9323
517.2439	5	S	15	1594.8482
614.2967	6	P	14	1507.8162
671.3181	7	G	13	1410.7634
784.4022	8	L	12	1353.7420
855.4393	9	A	11	1240.6579
1011.5404	10	R	10	1169.6208
1110.6088	11	V	9	1013.5197
1197.6408	12	S	8	914.4513
1325.6994	13	Q	7	827.4192
1438.7835	14	I	6	699.3607
1575.8424	15	H	5	586.2766
1735.8730	16	C	4	449.2177
1806.9102	17	A	3	289.1870
1877.9473	18	A	2	218.1499
	19	K	1	147.1128

[Click] to move table

Static Modifications:
C: 57.02146

KLNPQQQMKLQQLPSHLFIYF, MH+ 2601.4010, m/z 1301.2041

File: HEK293-C8-highAGC-360minDE15-75um15cm-2, Scan: 37882, Precursor m/z: 1301.2095, Charge: 2

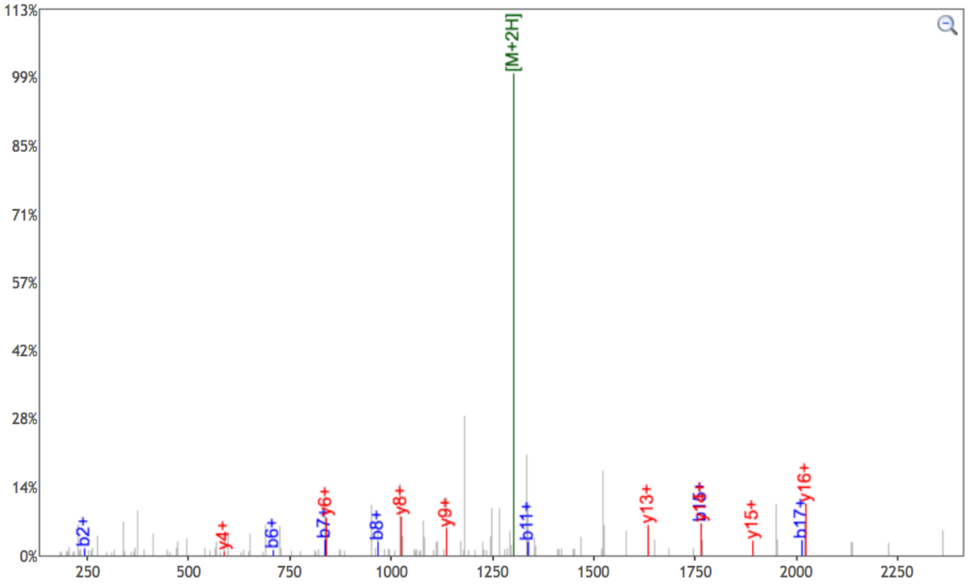

Click and drag in the plot to zoom X: ☑ Y: ☐ Zoom Out Print ☐Enable tooltip

b+	#	Seq	#	y+
129.1022	1	K	21	
242.1863	2	L	20	2473.3060
356.2292	3	N	19	2360.2220
453.2820	4	P	18	2246.1791
581.3406	5	Q	17	2149.1263
709.3991	6	Q	16	2021.0677
837.4577	7	Q	15	1893.0091
968.4982	8	M	14	1764.9506
1096.5932	9	K	13	1633.9101
1209.6772	10	L	12	1505.8151
1337.7358	11	Q	11	1392.7310
1465.7944	12	Q	10	1264.6725
1578.8785	13	L	9	1136.6139
1675.9312	14	P	8	1023.5298
1762.9633	15	S	7	926.4771
1900.0222	16	H	6	839.4450
2013.1062	17	L	5	702.3861
2160.1746	18	F	4	589.3021
2273.2587	19	I	3	442.2336
2436.3220	20	Y	2	329.1496
	21	F	1	166.0863

[Click] to move table

Static Modifications:
C: 57.02146

IREEAKSGEGGGGGTR, MH+ 1560.7725, m/z 520.9290

File: HEK293-C8-highAGC-360minDE15-75um15cm-1, Scan: 4881, Precursor m/z: 520.9326, Charge: 3

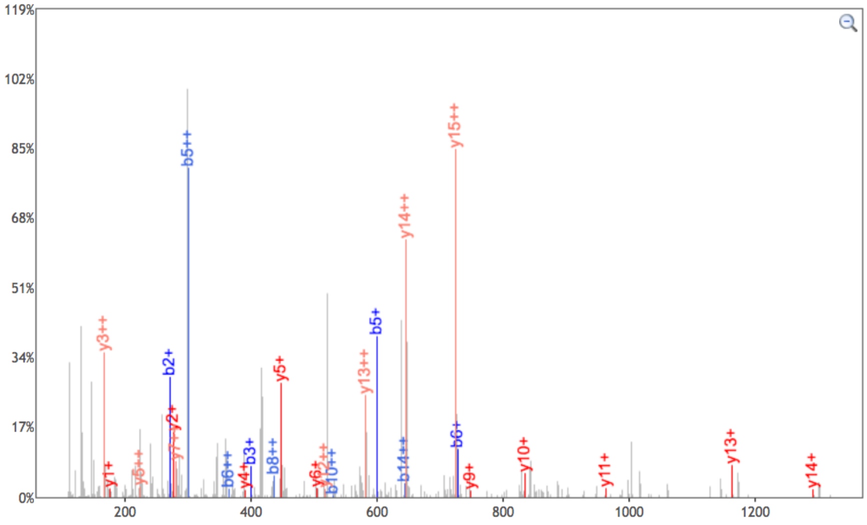

Click and drag in the plot to zoom X: ☑ Y: ☐ Zoom Out Print ☐Enable tooltip

b+	b2+	#	Seq	#	y+	y2+
114.0913	57.5493	1	I	16		
270.1925	135.5999	2	R	15	1447.6884	724.3478
399.2350	200.1212	3	E	14	1291.5873	646.2973
528.2776	264.6425	4	E	13	1162.5447	581.7760
599.3148	300.1610	5	A	12	1033.5021	517.2547
727.4097	364.2085	6	K	11	962.4650	481.7361
814.4417	407.7245	7	S	10	834.3700	417.6887
871.4632	436.2352	8	G	9	747.3380	374.1726
1000.5058	500.7565	9	E	8	690.3165	345.6619
1057.5273	529.2673	10	G	7	561.2739	281.1406
1114.5487	557.7780	11	G	6	504.2525	252.6299
1171.5702	586.2887	12	G	5	447.2310	224.1191
1228.5917	614.7995	13	G	4	390.2096	195.6084
1285.6131	643.3102	14	G	3	333.1881	167.0977
1386.6608	693.8340	15	T	2	276.1666	138.5870
		16	R	1	175.1190	88.0631

[Click] to move table

Static Modifications:
C: 57.02146

ERIIIETCSQR, MH+ 1404.7264, m/z 468.9136
File: HEK293-C8-highAGC-360minDE15-75um15cm-2, Scan: 3522, Precursor m/z: 468.9106, Charge: 3

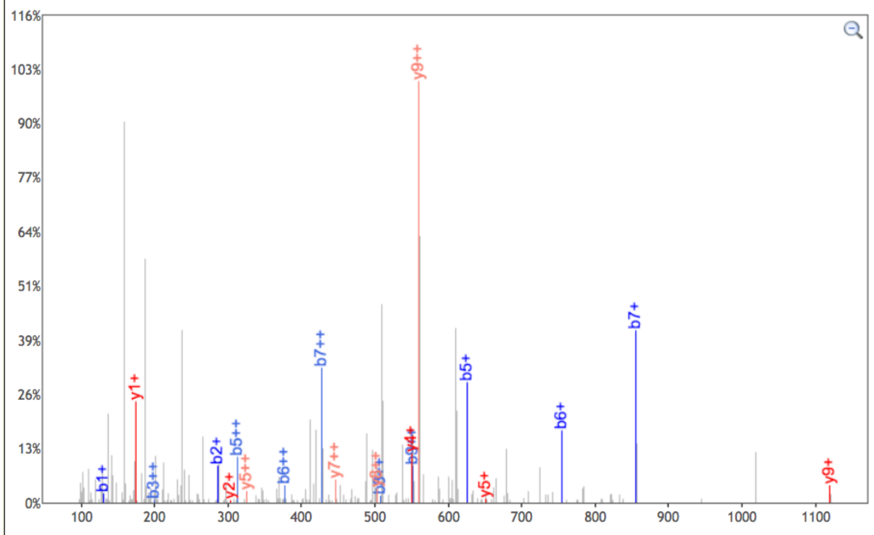

Click and drag in the plot to zoom X: Y: Zoom Out Print Enable tooltip

b+	b2+	#	Seq	#	y+	y2+
130.0499	65.5286	1	E	11		
286.1510	143.5791	2	R	10	1275.6838	638.3455
399.2350	200.1212	3	I	9	1119.5827	560.2950
512.3191	256.6632	4	I	8	1006.4986	503.7529
625.4032	313.2052	5	I	7	893.4145	447.2109
754.4458	377.7265	6	E	6	780.3305	390.6689
855.4934	428.2504	7	T	5	651.2879	326.1476
1015.5241	508.2657	8	C	4	550.2402	275.6237
1102.5561	551.7817	9	S	3	390.2096	195.6084
1230.6147	615.8110	10	Q	2	303.1775	152.0924
		11	R	1	175.1190	88.0631

[Click] to move table

Static Modifications:
C: 57.02146

LKEEVNWASANR, MH+ 1416.7230, m/z 472.9125
File: HEK293-C8-highAGC-360minDE15-75um15cm-2, Scan: 4505, Precursor m/z: 472.91544, Charge: 3

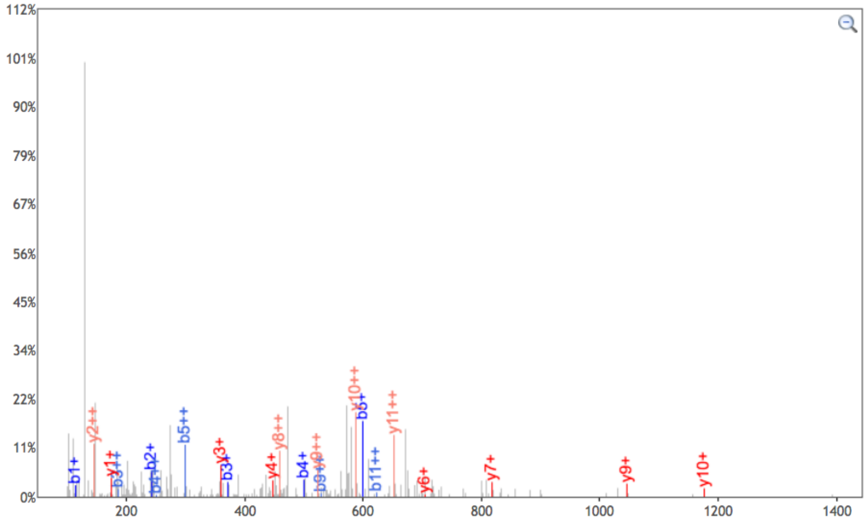

Click and drag in the plot to zoom X: Y: Zoom Out Print Enable tooltip

b+	b2+	#	Seq	#	y+	y2+
114.0913	57.5493	1	L	12		
242.1863	121.5968	2	K	11	1303.6389	652.3231
371.2289	186.1181	3	E	10	1175.5440	588.2756
500.2715	250.6394	4	E	9	1046.5014	523.7543
599.3399	300.1736	5	V	8	917.4588	459.2330
713.3828	357.1951	6	N	7	818.3904	409.6988
899.4621	450.2347	7	W	6	704.3474	352.6774
970.4993	485.7533	8	A	5	518.2681	259.6377
1057.5313	529.2693	9	S	4	447.2310	224.1191
1128.5684	564.7878	10	A	3	360.1990	180.6031
1242.6113	621.8093	11	N	2	289.1619	145.0846
		12	R	1	175.1190	88.0631

[Click] to move table

Static Modifications:
C: 57.02146

EGSREHNSLQSQKNIAK, MH+ 1925.9788, m/z 642.6644

File: HEK293-C8-highAGC-360minDE15-75um15cm-2, Scan: 13113, Precursor m/z: 642.6586, Charge: 3

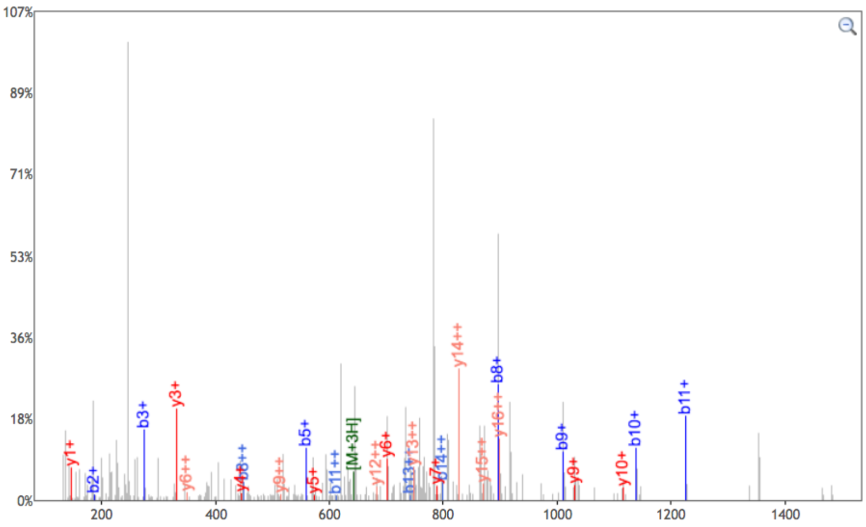

Click and drag in the plot to zoom X: Y: Zoom Out Print Enable tooltip

b+	b2+	#	Seq	#	y+	y2+
130.0499	65.5286	1	E	17		
187.0713	94.0393	2	G	16	1796.9362	898.9717
274.1034	137.5553	3	S	15	1739.9147	870.4610
430.2045	215.6059	4	R	14	1652.8827	826.9450
559.2471	280.1272	5	E	13	1496.7816	748.8944
696.3060	348.6566	6	H	12	1367.7390	684.3731
810.3489	405.6781	7	N	11	1230.6801	615.8437
897.3809	449.1941	8	S	10	1116.6371	558.8222
1010.4650	505.7361	9	L	9	1029.6051	515.3062
1138.5236	569.7654	10	Q	8	916.5211	458.7642
1225.5556	613.2814	11	S	7	788.4625	394.7349
1353.6142	677.3107	12	Q	6	701.4304	351.2189
1481.7091	741.3582	13	K	5	573.3719	287.1896
1595.7521	798.3797	14	N	4	445.2769	223.1421
1708.8361	854.9217	15	I	3	331.2340	166.1206
1779.8732	890.4403	16	A	2	218.1499	109.5786
		17	K	1	147.1128	74.0600

[Click] to move table

Static Modifications:
C: 57.02146

VGCLSPGLARVSQIHCAAK, MH+ 2024.0528, m/z 1012.5300

File: HEK293-acid-highAGC-360minDE15-75um25cm-1, Scan: 16550, Precursor m/z: 1012.5377, Charge: 2

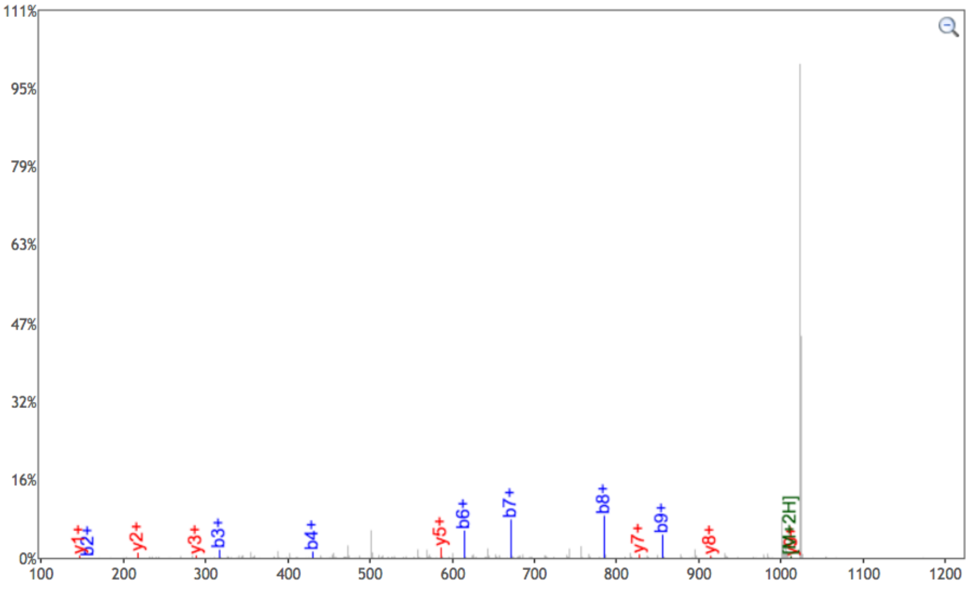

Click and drag in the plot to zoom X: Y: Zoom Out Print Enable tooltip

b+	#	Seq	#	y+
100.0757	1	V	19	
157.0972	2	G	18	1924.9844
317.1278	3	C	17	1867.9629
430.2119	4	L	16	1707.9323
517.2439	5	S	15	1594.8482
614.2967	6	P	14	1507.8162
671.3181	7	G	13	1410.7634
784.4022	8	L	12	1353.7420
855.4393	9	A	11	1240.6579
1011.5404	10	R	10	1169.6208
1110.6088	11	V	9	1013.5197
1197.6408	12	S	8	914.4513
1325.6994	13	Q	7	827.4192
1438.7835	14	I	6	699.3607
1575.8424	15	H	5	586.2766
1735.8730	16	C	4	449.2177
1806.9102	17	A	3	289.1870
1877.9473	18	A	2	218.1499
	19	K	1	147.1128

[Click] to move table

Static Modifications:
C: 57.02146

SSTGPGLYVTFKILK, MH+ 1610.9152, m/z 537.6433

File: HEK293-C8-highAGC-360minDE15-75um15cm-1, Scan: 10322, Precursor m/z: 537.6396, Charge: 3

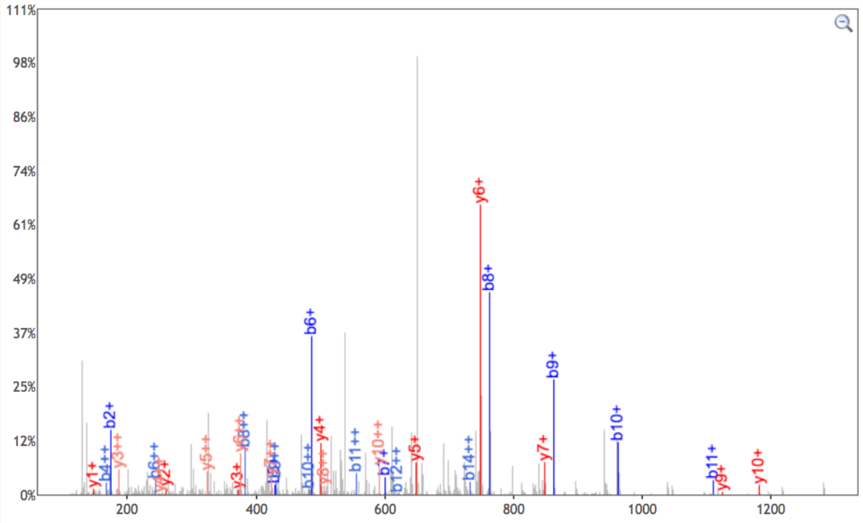

Click and drag in the plot to zoom X: Y: Zoom Out Print Enable tooltip

b+	b2+	#	Seq	#	y+	y2+
88.0393	44.5233	1	S	15		
175.0713	88.0393	2	S	14	1523.8832	762.4452
276.1190	138.5631	3	T	13	1436.8512	718.9292
333.1405	167.0739	4	G	12	1335.8035	668.4054
430.1932	215.6003	5	P	11	1278.7820	639.8946
487.2147	244.1110	6	G	10	1181.7293	591.3683
600.2988	300.6530	7	L	9	1124.7078	562.8575
763.3621	382.1847	8	Y	8	1011.6237	506.3155
862.4305	431.7189	9	V	7	848.5604	424.7838
963.4782	482.2427	10	T	6	749.4920	375.2496
1110.5466	555.7769	11	F	5	648.4443	324.7258
1238.6416	619.8244	12	K	4	501.3759	251.1916
1351.7256	676.3665	13	I	3	373.2809	187.1441
1464.8097	732.9085	14	L	2	260.1969	130.6021
		15	K	1	147.1128	74.0600

[Click] to move table

Static Modifications:
C: 57.02146

VLVVMVPLVGLIHLGWYR, MH+ 2064.2191, m/z 688.7445

File: HEK293-C8-highAGC-360minDE15-75um15cm-2, Scan: 36031, Precursor m/z: 688.74457, Charge: 3

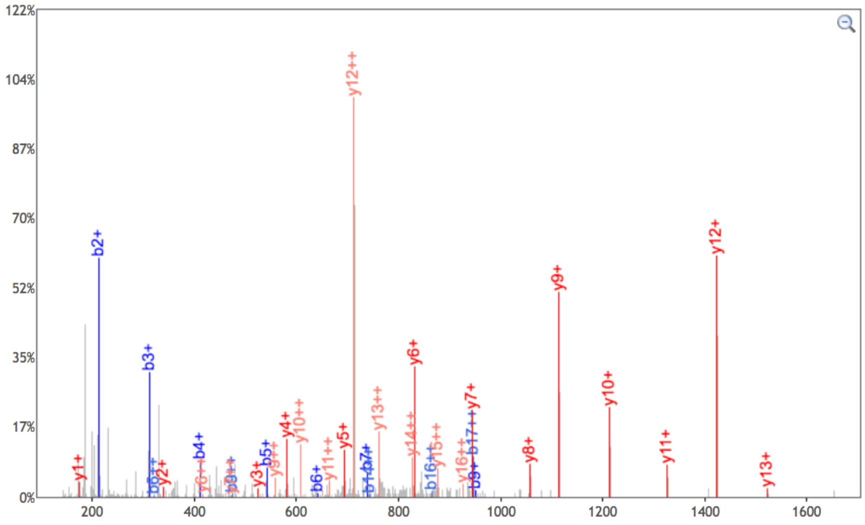

Click and drag in the plot to zoom X: Y: Zoom Out Print Enable tooltip

b+	b2+	#	Seq	#	y+	y2+
100.0757	50.5415	1	V	18		
213.1598	107.0835	2	L	17	1965.1507	983.0790
312.2282	156.6177	3	V	16	1852.0666	926.5369
411.2966	206.1519	4	V	15	1752.9982	877.0027
542.3371	271.6722	5	M	14	1653.9298	827.4685
641.4055	321.2064	6	V	13	1522.8893	761.9483
738.4582	369.7328	7	P	12	1423.8209	712.4141
851.5423	426.2748	8	L	11	1326.7681	663.8877
950.6107	475.8090	9	V	10	1213.6840	607.3457
1007.6322	504.3197	10	G	9	1114.6156	557.8115
1120.7163	560.8618	11	L	8	1057.5942	529.3007
1233.8003	617.4038	12	I	7	944.5101	472.7587
1370.8592	685.9333	13	H	6	831.4260	416.2167
1483.9433	742.4753	14	L	5	694.3671	347.6872
1540.9648	770.9860	15	G	4	581.2831	291.1452
1727.0441	864.0257	16	W	3	524.2616	262.6344
1890.1074	945.5573	17	Y	2	338.1823	169.5948
		18	R	1	175.1190	88.0631

[Click] to move table

Static Modifications:
C: 57.02146

VGNMSESELGR, MH+ 1178.5470, m/z 589.7771

File: HEK293-C8-highAGC-360minDE15-75um15cm-3, Scan: 3503, Precursor m/z: 589.78296, Charge: 2

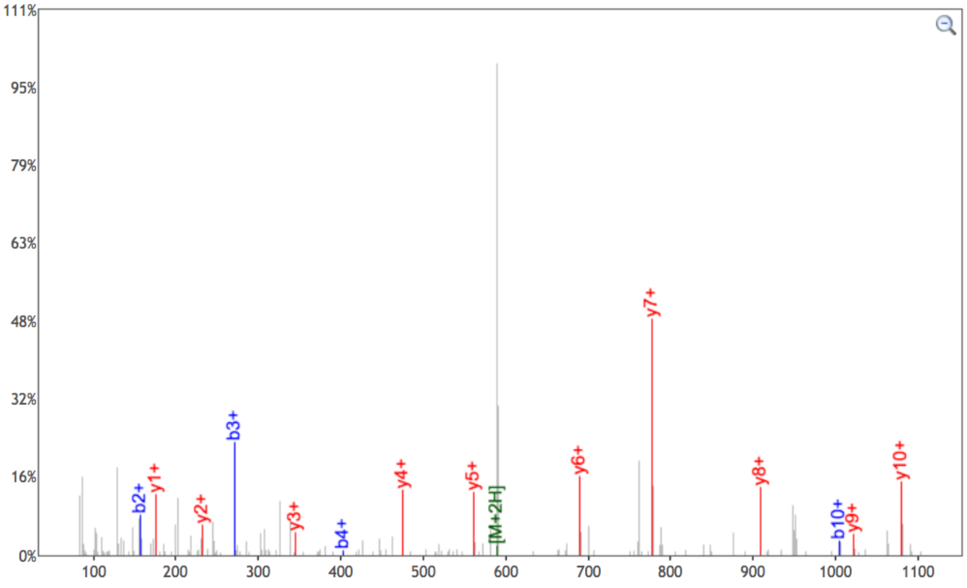

Click and drag in the plot to zoom X: Y: Zoom Out Print Enable tooltip

b+	#	Seq	#	y+
100.0757	1	V	11	
157.0972	2	G	10	1079.4786
271.1401	3	N	9	1022.4571
402.1806	4	M	8	908.4142
489.2126	5	S	7	777.3737
618.2552	6	E	6	690.3417
705.2872	7	S	5	561.2991
834.3298	8	E	4	474.2671
947.4139	9	L	3	345.2245
1004.4353	10	G	2	232.1404
	11	R	1	175.1190

[Click] to move table

Static Modifications:
C: 57.02146

CTSVMTCWSILLNR, MH+ 1740.8230, m/z 580.9458

File: HEK293-C8-highAGC-360minDE15-75um15cm-1, Scan: 26111, Precursor m/z: 580.9489, Charge: 3

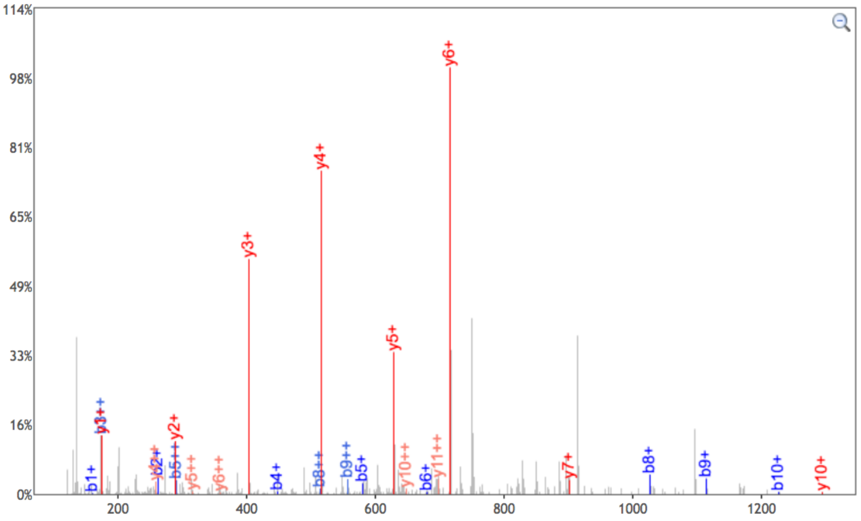

Click and drag in the plot to zoom X: Y: Zoom Out Print Enable tooltip

b+	b2+	#	Seq	#	y+	y2+
161.0379	81.0226	1	C	14		
262.0856	131.5464	2	T	13	1580.7923	790.8998
349.1176	175.0625	3	S	12	1479.7447	740.3760
448.1860	224.5967	4	V	11	1392.7126	696.8600
579.2265	290.1169	5	M	10	1293.6442	647.3257
680.2742	340.6407	6	T	9	1162.6037	581.8055
840.3048	420.6561	7	C	8	1061.5561	531.2817
1026.3842	513.6957	8	W	7	901.5254	451.2663
1113.4162	557.2117	9	S	6	715.4461	358.2267
1226.5003	613.7538	10	I	5	628.4141	314.7107
1339.5843	670.2958	11	L	4	515.3300	258.1686
1452.6684	726.8378	12	L	3	402.2459	201.6266
1566.7113	783.8593	13	N	2	289.1619	145.0846
		14	R	1	175.1190	88.0631

[Click] to move table

Static Modifications:
C: 57.02146

FQSSAVTALALQEASEAYLVGLFEDTNLCAIHAK, MH+ 3667.8312, m/z 1223.2819

File: HEK293-C8-highAGC-360minDE15-75um15cm-1, Scan: 40618, Precursor m/z: 1223.2932, Charge: 3

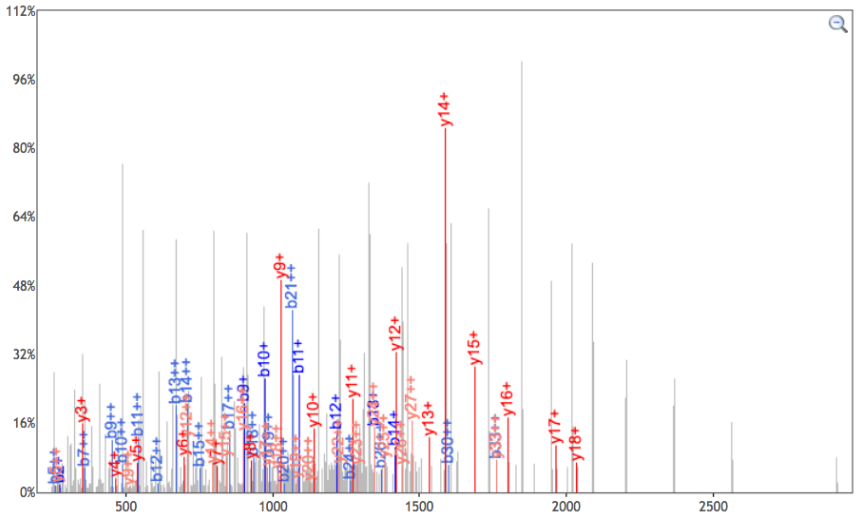

Click and drag in the plot to zoom X: Y: Zoom Out Print Enable tooltip

b+	b2+	#	Seq	#	y+	y2+
148.0757	74.5415	1	F	34		
276.1343	138.5708	2	Q	33	3520.7628	1760.8850
363.1663	182.0868	3	S	32	3392.7042	1696.8558
450.1983	225.6028	4	S	31	3305.6722	1653.3397
521.2354	261.1214	5	A	30	3218.6402	1609.8237
620.3039	310.6556	6	V	29	3147.6031	1574.3052
721.3515	361.1794	7	T	28	3048.5347	1524.7710
792.3886	396.6980	8	A	27	2947.4870	1474.2471
905.4727	453.2400	9	L	26	2876.4499	1438.7286
976.5098	488.7585	10	A	25	2763.3658	1382.1865
1089.5939	545.3006	11	L	24	2692.3287	1346.6680
1217.6525	609.3299	12	Q	23	2579.2446	1290.1260
1346.6951	673.8512	13	E	22	2451.1860	1226.0967
1417.7322	709.3697	14	A	21	2322.1435	1161.5754
1504.7642	752.8857	15	S	20	2251.1063	1126.0568
1633.8068	817.4070	16	E	19	2164.0743	1082.5408
1704.8439	852.9256	17	A	18	2035.0317	1018.0195
1867.9072	934.4573	18	Y	17	1963.9946	982.5009
1980.9913	990.9993	19	L	16	1800.9313	900.9693
2080.0597	1040.5335	20	V	15	1687.8472	844.4272
2137.0812	1069.0442	21	G	14	1588.7788	794.8930
2250.1652	1125.5863	22	L	13	1531.7573	766.3823
2397.2337	1199.1205	23	F	12	1418.6733	709.8403
2526.2762	1263.6418	24	E	11	1271.6049	636.3061
2641.3032	1321.1552	25	D	10	1142.5623	571.7848
2742.3509	1371.6791	26	T	9	1027.5353	514.2713
2856.3938	1428.7005	27	N	8	926.4876	463.7475
2969.4779	1485.2426	28	L	7	812.4447	406.7260
3129.5085	1565.2579	29	C	6	699.3607	350.1840
3200.5456	1600.7764	30	A	5	539.3300	270.1686
3313.6297	1657.3185	31	I	4	468.2929	234.6501
3450.6886	1725.8479	32	H	3	355.2088	178.1081
3521.7257	1761.3665	33	A	2	218.1499	109.5786
		34	K	1	147.1128	74.0600

[Click] to move table

Static Modifications:
C: 57.02146

GPAYEKR, MH+ 820.4312, m/z 410.7192

File: HEK293-C8-highAGC-360minDE15-75um15cm-1, Scan: 1053, Precursor m/z: 410.72186, Charge: 2

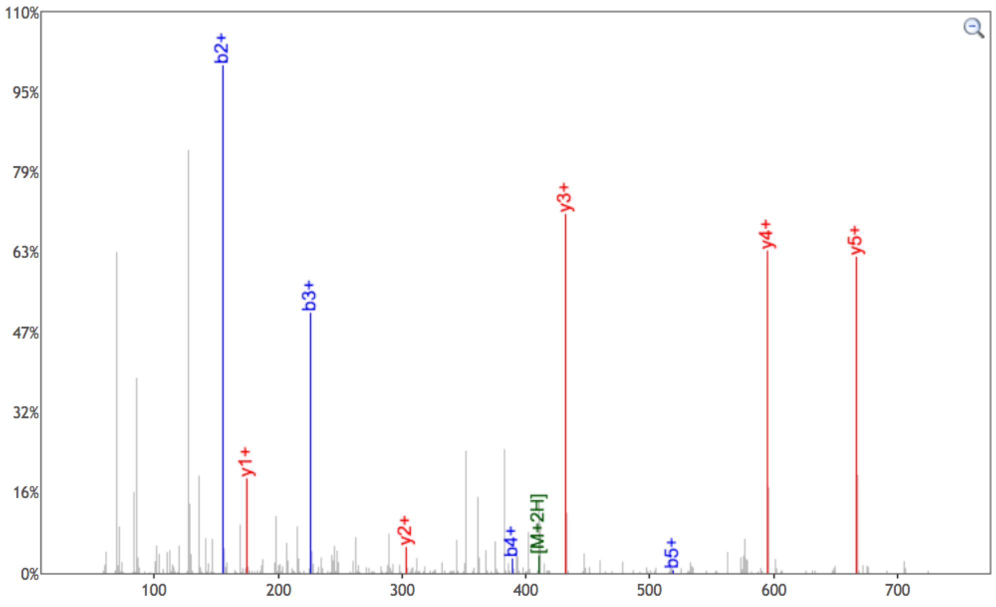

Click and drag in the plot to zoom X: Y: Zoom Out Print Enable tooltip

b+	#	Seq	#	y+
58.0287	1	G	7	
155.0815	2	P	6	763.4097
226.1186	3	A	5	666.3570
389.1819	4	Y	4	595.3198
518.2245	5	E	3	432.2565
646.3195	6	K	2	303.2139
	7	R	1	175.1190

[Click] to move table

Static Modifications:
C: 57.02146

RVEDEVNSGVGQDGSLLSSPFLK, MH+ 2433.2256, m/z 811.7467

File: HEK293-C8-highAGC-360minDE15-75um15cm-1, Scan: 20614, Precursor m/z: 811.7525, Charge: 3

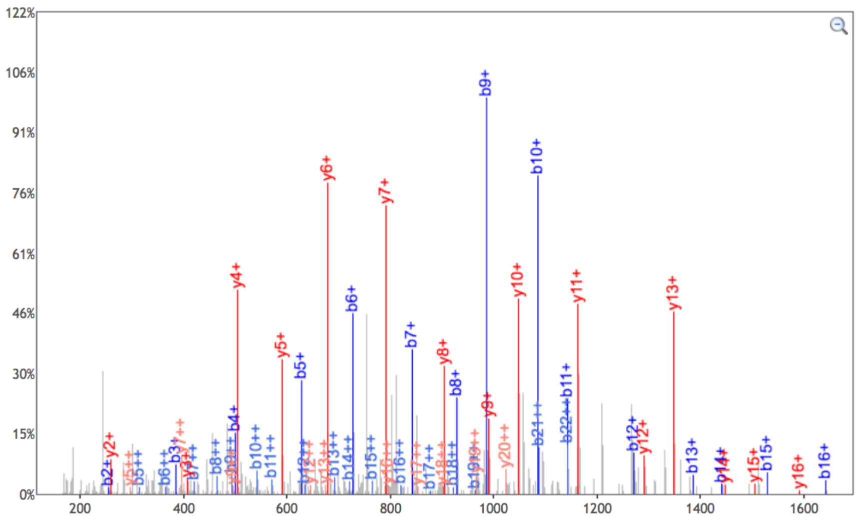

Click and drag in the plot to zoom X: Y: Zoom Out Print Enable tooltip

b+	b2+	#	Seq	#	y+	y2+
157.1084	79.0578	1	R	23		
256.1768	128.5920	2	V	22	2277.1245	1139.0659
385.2194	193.1133	3	E	21	2178.0561	1089.5317
500.2463	250.6268	4	D	20	2049.0135	1025.0104
629.2889	315.1481	5	E	19	1933.9865	967.4969
728.3573	364.6823	6	V	18	1804.9440	902.9756
842.4003	421.7038	7	N	17	1705.8755	853.4414
929.4323	465.2198	8	S	16	1591.8326	796.4199
986.4538	493.7305	9	G	15	1504.8006	752.9039
1085.5222	543.2647	10	V	14	1447.7791	724.3932
1142.5436	571.7755	11	G	13	1348.7107	674.8590
1270.6022	635.8047	12	Q	12	1291.6892	646.3483
1385.6292	693.3182	13	D	11	1163.6307	582.3190
1442.6506	721.8290	14	G	10	1048.6037	524.8055
1529.6827	765.3450	15	S	9	991.5823	496.2948
1642.7667	821.8870	16	L	8	904.5502	452.7788
1755.8508	878.4290	17	L	7	791.4662	396.2367
1842.8828	921.9450	18	S	6	678.3821	339.6947
1929.9148	965.4611	19	S	5	591.3501	296.1787
2026.9676	1013.9874	20	P	4	504.3180	252.6627
2174.0360	1087.5216	21	F	3	407.2653	204.1363
2287.1201	1144.0637	22	L	2	260.1969	130.6021
		23	K	1	147.1128	74.0600

[Click] to move table

Static Modifications:
C: 57.02146

GFLAGYVVAK, MH+ 1024.5826, m/z 512.7949
File: HEK293-C8-highAGC-360minDE15-75um15cm-2, Scan: 13612, Precursor m/z: 512.7979, Charge: 2

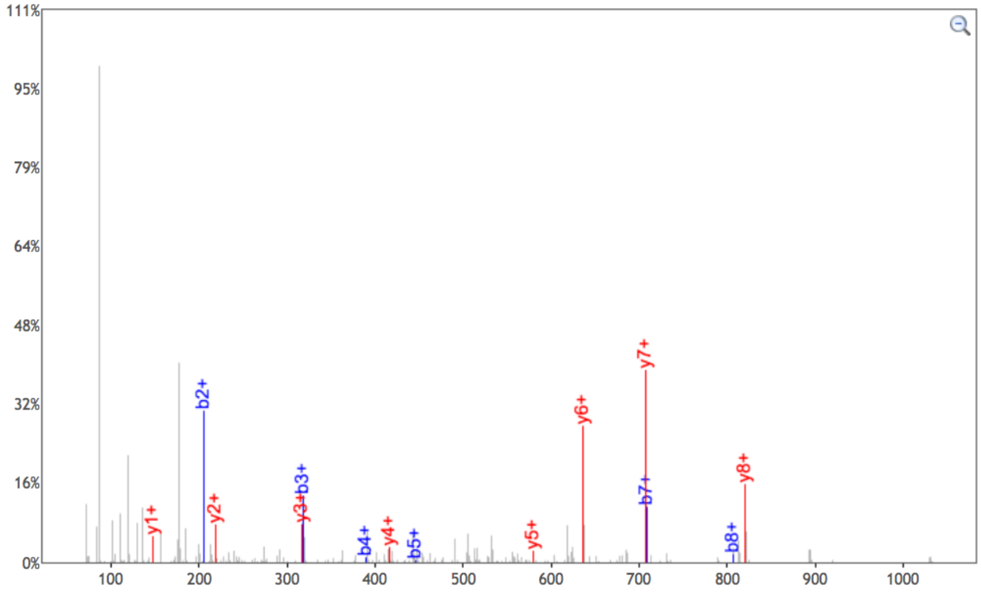

Click and drag in the plot to zoom X: Y: Zoom Out Print Enable tooltip

b+	#	Seq	#	y+
58.0287	1	G	10	
205.0972	2	F	9	967.5611
318.1812	3	L	8	820.4927
389.2183	4	A	7	707.4087
446.2398	5	G	6	636.3715
609.3031	6	Y	5	579.3501
708.3715	7	V	4	416.2867
807.4400	8	V	3	317.2183
878.4771	9	A	2	218.1499
	10	K	1	147.1128

[Click] to move table

Static Modifications:
C: 57.02146

NKGDLDSAFLGK, MH+ 1264.6532, m/z 632.8302
File: HEK293-C8-highAGC-360minDE15-75um15cm-1, Scan: 12626, Precursor m/z: 632.83563, Charge: 2

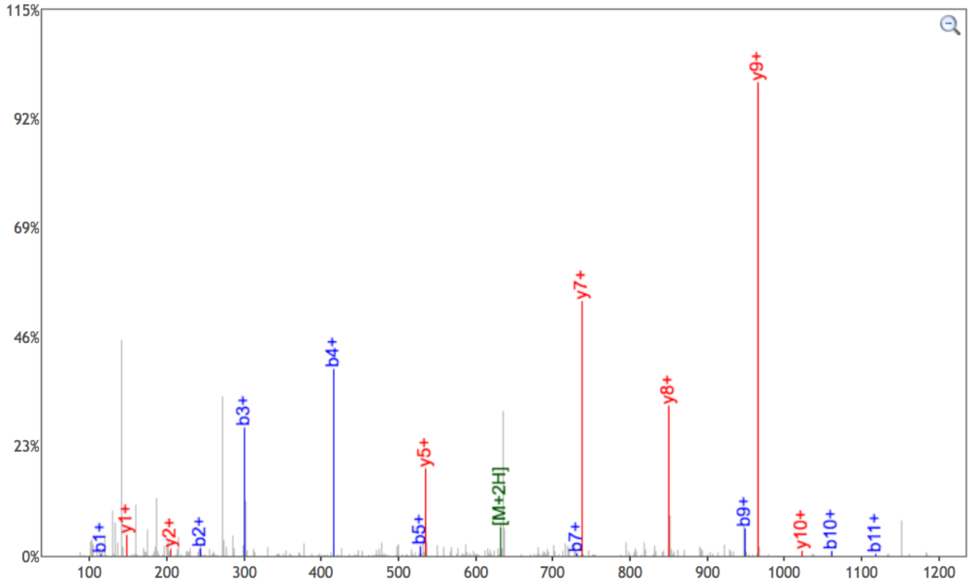

Click and drag in the plot to zoom X: Y: Zoom Out Print Enable tooltip

b+	#	Seq	#	y+
115.0502	1	N	12	
243.1452	2	K	11	1150.6103
300.1666	3	G	10	1022.5153
415.1936	4	D	9	965.4938
528.2776	5	L	8	850.4669
643.3046	6	D	7	737.3828
730.3366	7	S	6	622.3559
801.3737	8	A	5	535.3239
948.4421	9	F	4	464.2867
1061.5262	10	L	3	317.2183
1118.5477	11	G	2	204.1343
	12	K	1	147.1128

[Click] to move table

Static Modifications:
C: 57.02146

GSWFVQALCSILEEHGKELEIMQILTR, MH+ 3187.6278, m/z 797.6624

File: HEK293-C8-highAGC-360minDE15-75um15cm-2, Scan: 37330, Precursor m/z: 797.6665, Charge: 4

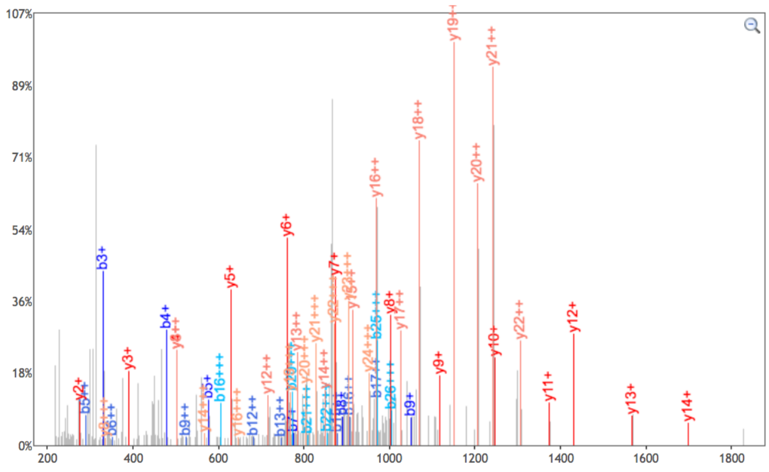

Click and drag in the plot to zoom X: ☑ Y: ☐ Zoom Out Print ☐Enable tooltip

b+	b2+	b3+	#	Seq	#	y+	y2+	y3+
58.0287	29.5180	20.0144	1	G	27			
145.0608	73.0340	49.0251	2	S	26	3130.6064	1565.8068	1044.2070
331.1401	166.0737	111.0515	3	W	25	3043.5744	1522.2908	1015.1963
478.2085	239.6079	160.0743	4	F	24	2857.4950	1429.2512	953.1699
577.2769	289.1421	193.0972	5	V	23	2710.4266	1355.7170	904.1471
705.3355	353.1714	235.7833	6	Q	22	2611.3582	1306.1827	871.1243
776.3726	388.6899	259.4624	7	A	21	2483.2996	1242.1535	828.4381
889.4567	445.2320	297.1571	8	L	20	2412.2625	1206.6349	804.7590
1049.4873	525.2473	350.5006	9	C	19	2299.1785	1150.0929	767.0643
1136.5193	568.7633	379.5113	10	S	18	2139.1478	1070.0775	713.7208
1249.6034	625.3053	417.2060	11	I	17	2052.1158	1026.5615	684.7101
1362.6875	681.8474	454.9007	12	L	16	1939.0317	970.0195	647.0154
1491.7301	746.3687	497.9149	13	E	15	1825.9477	913.4775	609.3207
1620.7727	810.8900	540.9291	14	E	14	1696.9051	848.9562	566.3065
1757.8316	879.4194	586.6154	15	H	13	1567.8625	784.4349	523.2923
1814.8530	907.9302	605.6225	16	G	12	1430.8036	715.9054	477.6060
1942.9480	971.9776	648.3208	17	K	11	1373.7821	687.3947	458.5989
2071.9906	1036.4989	691.3350	18	E	10	1245.6871	623.3472	415.9006
2185.0746	1093.0410	729.0297	19	L	9	1116.6445	558.8259	372.8864
2314.1172	1157.5623	772.0439	20	E	8	1003.5605	502.2839	335.1917
2427.2013	1214.1043	809.7386	21	I	7	874.5179	437.7626	292.1775
2558.2418	1279.6245	853.4188	22	M	6	761.4338	381.2205	254.4828
2686.3004	1343.6538	896.1050	23	Q	5	630.3933	315.7003	210.8026
2799.3844	1400.1959	933.7997	24	I	4	502.3348	251.6710	168.1164
2912.4685	1456.7379	971.4943	25	L	3	389.2507	195.1290	130.4217
3013.5162	1507.2617	1005.1769	26	T	2	276.1666	138.5870	92.7271
			27	R	1	175.1190	88.0631	59.0445

[Click] to move table

Static Modifications:
C: 57.02146

YEILALLK, MH+ 962.5921, m/z 481.7997

File: HEK293-C8-highAGC-360minDE15-75um15cm-1, Scan: 18256, Precursor m/z: 481.80188, Charge: 2

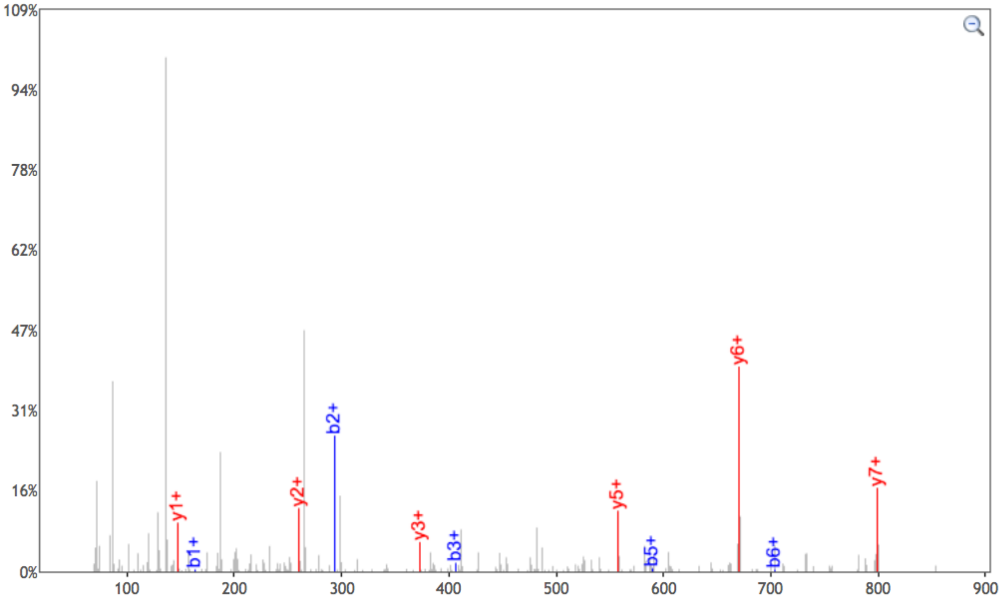

Click and drag in the plot to zoom X: ☑ Y: ☐ Zoom Out Print ☐Enable tooltip

b+	#	Seq	#	y+
164.0706	1	Y	8	
293.1132	2	E	7	799.5288
406.1973	3	I	6	670.4862
519.2813	4	L	5	557.4021
590.3184	5	A	4	444.3180
703.4025	6	L	3	373.2809
816.4866	7	L	2	260.1969
	8	K	1	147.1128

[Click] to move table

Static Modifications:
C: 57.02146

VRDFGGR, MH+ 806.4268, m/z 403.7170

File: HEK293-C8-highAGC-360minDE15-75um15cm-1, Scan: 4192, Precursor m/z: 403.7134, Charge: 2

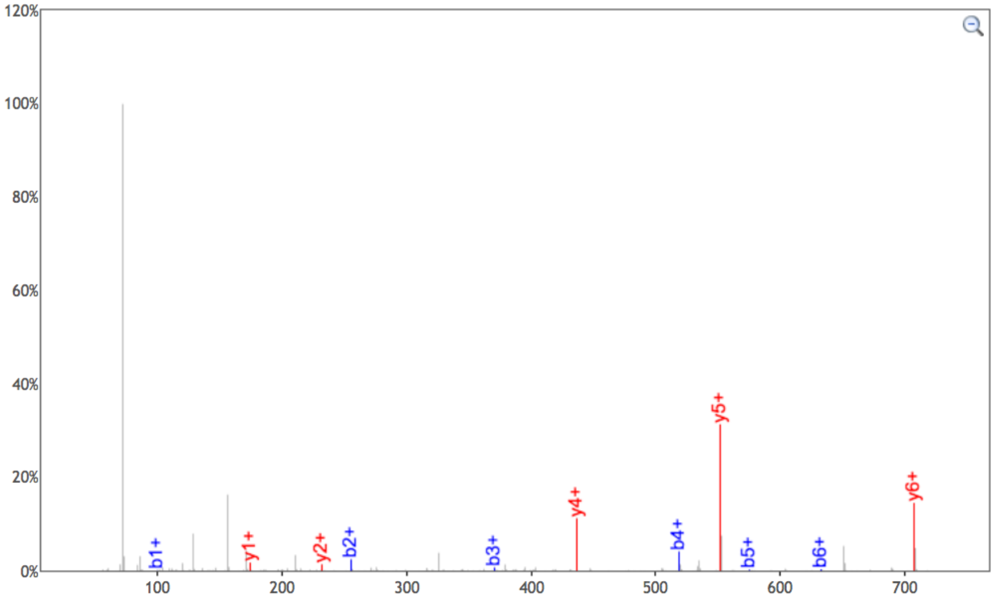

Click and drag in the plot to zoom X: Y: Zoom Out Print Enable tooltip

b+	#	Seq	#	y+
100.0757	1	V	7	
256.1768	2	R	6	707.3583
371.2037	3	D	5	551.2572
518.2722	4	F	4	436.2303
575.2936	5	G	3	289.1619
632.3151	6	G	2	232.1404
	7	R	1	175.1190

[Click] to move table

Static Modifications:
C: 57.02146

GPLRHRGWEGTTWGLWTLLCLWSQPTGW, MH+ 3351.6633, m/z 838.6713

File: HEK293-C8-highAGC-360minDE15-75um15cm-1, Scan: 23724, Precursor m/z: 838.6671, Charge: 4

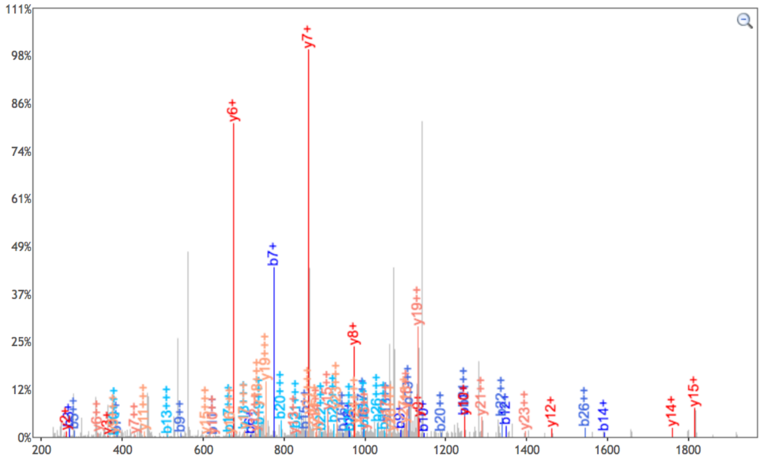

Click and drag in the plot to zoom X: Y: Zoom Out Print Enable tooltip

b+	b2+	b3+	#	Seq	#	y+	y2+	y3+
58.0287	29.5180	20.0144	1	G	28			
155.0815	78.0444	52.3654	2	P	27	3294.6418	1647.8245	1098.8855
268.1656	134.5864	90.0600	3	L	26	3197.5890	1599.2982	1066.5345
424.2667	212.6370	142.0937	4	R	25	3084.5050	1542.7561	1028.8398
561.3256	281.1664	187.7800	5	H	24	2928.4039	1464.7056	976.8061
717.4267	359.2170	239.8138	6	R	23	2791.3449	1396.1761	931.1198
774.4482	387.7277	258.8209	7	G	22	2635.2438	1318.1256	879.0861
960.5275	480.7674	320.8473	8	W	21	2578.2224	1289.6148	860.0790
1089.5701	545.2887	363.8615	9	E	20	2392.1431	1196.5752	798.0525
1146.5915	573.7994	382.8687	10	G	19	2263.1005	1132.0539	755.0383
1247.6392	624.3232	416.5513	11	T	18	2206.0790	1103.5431	736.0312
1348.6869	674.8471	450.2338	12	T	17	2105.0313	1053.0193	702.3486
1534.7662	767.8867	512.2603	13	W	16	2003.9836	1002.4955	668.6661
1591.7877	796.3975	531.2674	14	G	15	1817.9043	909.4558	606.6396
1704.8717	852.9395	568.9621	15	L	14	1760.8829	880.9451	587.6325
1890.9510	945.9792	630.9885	16	W	13	1647.7988	824.4030	549.9378
1991.9987	996.5030	664.6711	17	T	12	1461.7195	731.3634	487.9113
2105.0828	1053.0450	702.3658	18	L	11	1360.6718	680.8395	454.2288
2218.1669	1109.5871	740.0605	19	L	10	1247.5878	624.2975	416.5341
2378.1975	1189.6024	793.4040	20	C	9	1134.5037	567.7555	378.8394
2491.2816	1246.1444	831.0987	21	L	8	974.4730	487.7402	325.4959
2677.3609	1339.1841	893.1251	22	W	7	861.3890	431.1981	287.8012
2764.3929	1382.7001	922.1358	23	S	6	675.3097	338.1585	225.7747
2892.4515	1446.7294	964.8220	24	Q	5	588.2776	294.6425	196.7641
2989.5042	1495.2558	997.1729	25	P	4	460.2191	230.6132	154.0779
3090.5519	1545.7796	1030.8555	26	T	3	363.1663	182.0868	121.7269
3147.5734	1574.2903	1049.8626	27	G	2	262.1186	131.5629	88.0444
			28	W	1	205.0972	103.0522	69.0372

[Click] to move table

Static Modifications:
C: 57.02146

WPYTHLAWMAALF, MH+ 1606.7875, m/z 803.8974

File: HEK293-C8-highAGC-360minDE15-75um15cm-3, Scan: 23080, Precursor m/z: 803.9041, Charge: 2

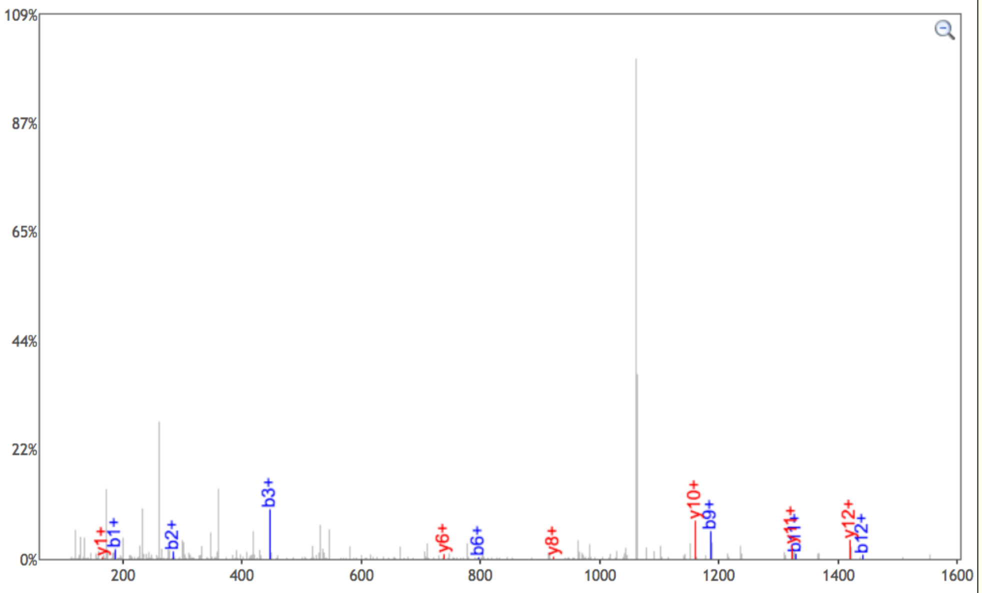

Click and drag in the plot to zoom X: Y: Zoom Out Print Enable tooltip

b+	#	Seq	#	y+
187.0866	1	W	13	
284.1394	2	P	12	1420.7082
447.2027	3	Y	11	1323.6554
548.2504	4	T	10	1160.5921
685.3093	5	H	9	1059.5444
798.3933	6	L	8	922.4855
869.4304	7	A	7	809.4015
1055.5098	8	W	6	738.3643
1186.5502	9	M	5	552.2850
1257.5874	10	A	4	421.2445
1328.6245	11	A	3	350.2074
1441.7085	12	L	2	279.1703
	13	F	1	166.0863

[Click] to move table

Static Modifications:
C: 57.02146

NQGAKWSGWAGPTPMEPSKLK, MH+ 2270.1386, m/z 568.2901

File: HEK293-C8-highAGC-360minDE15-75um15cm-2, Scan: 14565, Precursor m/z: 568.28766, Charge: 4

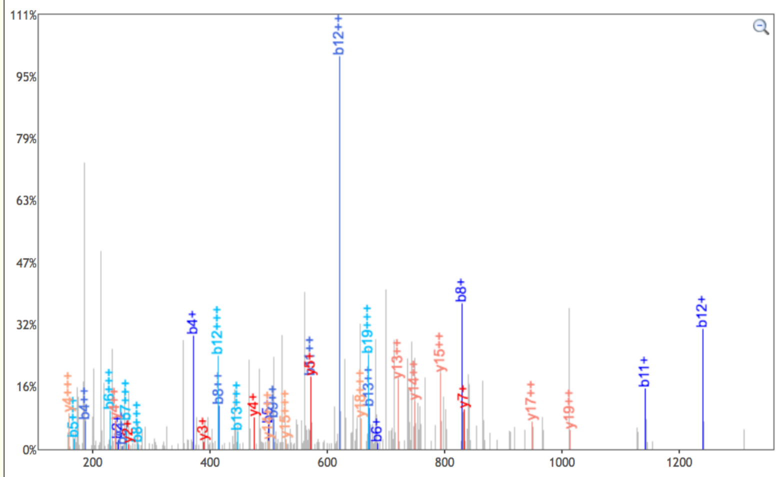

Click and drag in the plot to zoom X: Y: Zoom Out Print Enable tooltip

b+	b2+	b3+	#	Seq	#	y+	y2+	y3+
115.0502	58.0287	39.0216	1	N	21			
243.1088	122.0580	81.7078	2	Q	20	2156.0957	1078.5515	719.3701
300.1302	150.5688	100.7149	3	G	19	2028.0371	1014.5222	676.6839
371.1674	186.0873	124.3940	4	A	18	1971.0157	986.0115	657.6767
499.2623	250.1348	167.0923	5	K	17	1899.9786	950.4929	633.9977
685.3416	343.1745	229.1187	6	W	16	1771.8836	886.4454	591.2994
772.3737	386.6905	258.1294	7	S	15	1585.8043	793.4058	529.2729
829.3951	415.2012	277.1366	8	G	14	1498.7723	749.8898	500.2623
1015.4744	508.2409	339.1630	9	W	13	1441.7508	721.3790	481.2551
1086.5116	543.7594	362.8420	10	A	12	1255.6715	628.3394	419.2287
1143.5330	572.2701	381.8492	11	G	11	1184.6344	592.8208	395.5496
1240.5858	620.7965	414.2001	12	P	10	1127.6129	564.3101	376.5425
1341.6335	671.3204	447.8827	13	T	9	1030.5601	515.7837	344.1916
1438.6862	719.8467	480.2336	14	P	8	929.5125	465.2599	310.5090
1569.7267	785.3670	523.9138	15	M	7	832.4597	416.7335	278.1581
1698.7693	849.8883	566.9280	16	E	6	701.4192	351.2132	234.4779
1795.8221	898.4147	599.2789	17	P	5	572.3766	286.6919	191.4637
1882.8541	941.9307	628.2895	18	S	4	475.3239	238.1656	159.1128
2010.9491	1005.9782	670.9879	19	K	3	388.2918	194.6496	130.1021
2124.0331	1062.5202	708.6826	20	L	2	260.1969	130.6021	87.4038
			21	K	1	147.1128	74.0600	49.7091

[Click] to move table

Static Modifications:
C: 57.02146

AEAMPLFSLFVFILEK, MH+ 1855.0074, m/z 619.0073

File: HEK293-C8-highAGC-360minDE15-75um15cm-1, Scan: 24857, Precursor m/z: 619.0043, Charge: 3

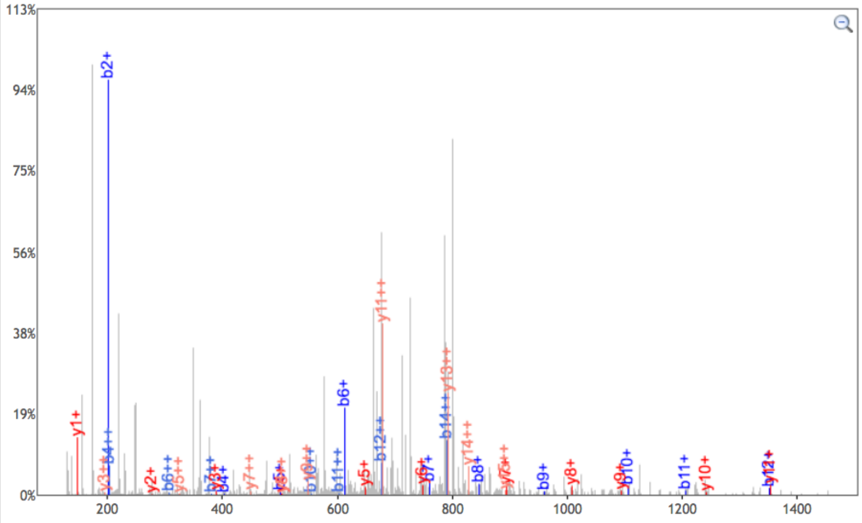

Click and drag in the plot to zoom X: ☑ Y: ☐ Zoom Out Print ☐Enable tooltip

b+	b2+	#	Seq	#	y+	y2+
72.0444	36.5258	1	A	16		
201.0870	101.0471	2	E	15	1783.9703	892.4888
272.1241	136.5657	3	A	14	1654.9277	827.9675
403.1646	202.0859	4	M	13	1583.8906	792.4489
500.2173	250.6123	5	P	12	1452.8501	726.9287
613.3014	307.1543	6	L	11	1355.7973	678.4023
760.3698	380.6885	7	F	10	1242.7133	621.8603
847.4019	424.2046	8	S	9	1095.6449	548.3261
960.4859	480.7466	9	L	8	1008.6128	504.8101
1107.5543	554.2808	10	F	7	895.5288	448.2680
1206.6227	603.8150	11	V	6	748.4604	374.7338
1353.6912	677.3492	12	F	5	649.3919	325.1996
1466.7752	733.8912	13	I	4	502.3235	251.6654
1579.8593	790.4333	14	L	3	389.2395	195.1234
1708.9019	854.9546	15	E	2	276.1554	138.5813
		16	K	1	147.1128	74.0600

[Click] to move table

Static Modifications:

C: 57.02146

WALGPWWLAPATCPI, MH+ 1738.8774, m/z 869.9423
File: HEK293-C8-highAGC-360minDE15-75um15cm-1, Scan: 29021, Precursor m/z: 869.9338, Charge: 2

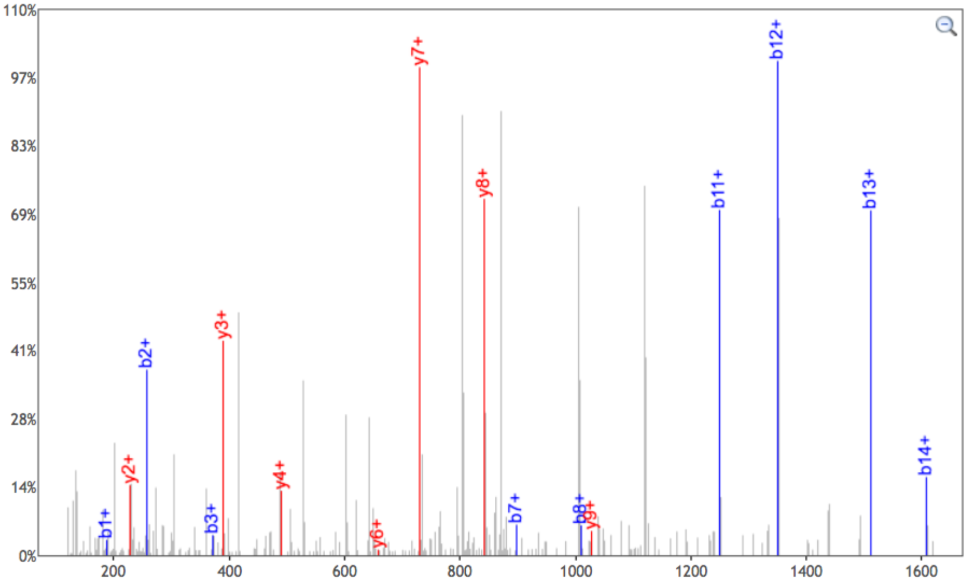

Click and drag in the plot to zoom X: ☑ Y: ☐ Zoom Out Print ☐Enable tooltip

b+	#	Seq	#	y+
187.0866	1	W	15	
258.1237	2	A	14	1552.7981
371.2078	3	L	13	1481.7610
428.2292	4	G	12	1368.6769
525.2820	5	P	11	1311.6554
711.3613	6	W	10	1214.6027
897.4406	7	W	9	1028.5234
1010.5247	8	L	8	842.4440
1081.5618	9	A	7	729.3600
1178.6146	10	P	6	658.3229
1249.6517	11	A	5	561.2701
1350.6994	12	T	4	490.2330
1510.7300	13	C	3	389.1853
1607.7828	14	P	2	229.1547
	15	I	1	132.1019

[Click] to move table

Static Modifications:
C: 57.02146

IREEAKSGEGGGGGTR, MH+ 1560.7725, m/z 520.9290
File: HEK293-C8-highAGC-360minDE15-75um15cm-3, Scan: 4698, Precursor m/z: 520.9334, Charge: 3

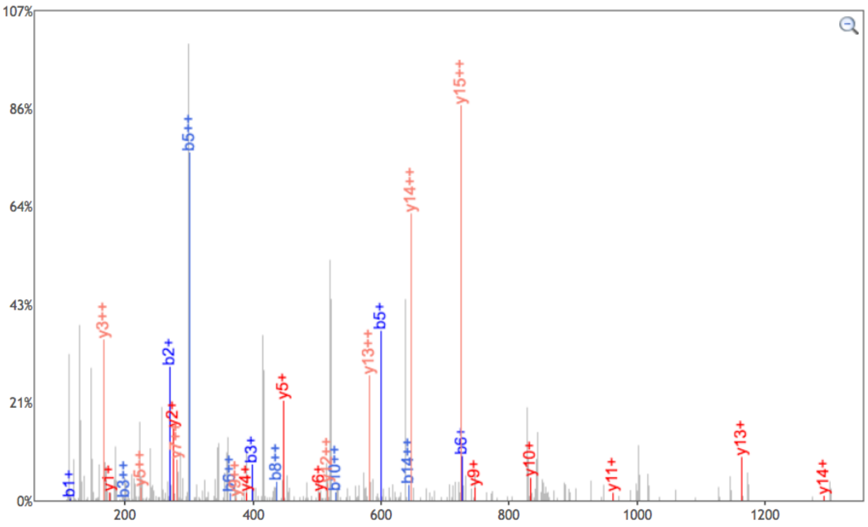

Click and drag in the plot to zoom X: ☑ Y: ☐ Zoom Out Print ☐Enable tooltip

b+	b2+	#	Seq	#	y+	y2+
114.0913	57.5493	1	I	16		
270.1925	135.5999	2	R	15	1447.6884	724.3478
399.2350	200.1212	3	E	14	1291.5873	646.2973
528.2776	264.6425	4	E	13	1162.5447	581.7760
599.3148	300.1610	5	A	12	1033.5021	517.2547
727.4097	364.2085	6	K	11	962.4650	481.7361
814.4417	407.7245	7	S	10	834.3700	417.6887
871.4632	436.2352	8	G	9	747.3380	374.1726
1000.5058	500.7565	9	E	8	690.3165	345.6619
1057.5273	529.2673	10	G	7	561.2739	281.1406
1114.5487	557.7780	11	G	6	504.2525	252.6299
1171.5702	586.2887	12	G	5	447.2310	224.1191
1228.5917	614.7995	13	G	4	390.2096	195.6084
1285.6131	643.3102	14	G	3	333.1881	167.0977
1386.6608	693.8340	15	T	2	276.1666	138.5870
		16	R	1	175.1190	88.0631

[Click] to move table

Static Modifications:
C: 57.02146

DGLAPTWSLPCPLLPGPLPPDAALPGAVR, MH+ 2948.5703, m/z 983.5283

File: HEK293-acid-highAGC-360minDE15-75um25cm-1, Scan: 36173, Precursor m/z: 983.5316, Charge: 3

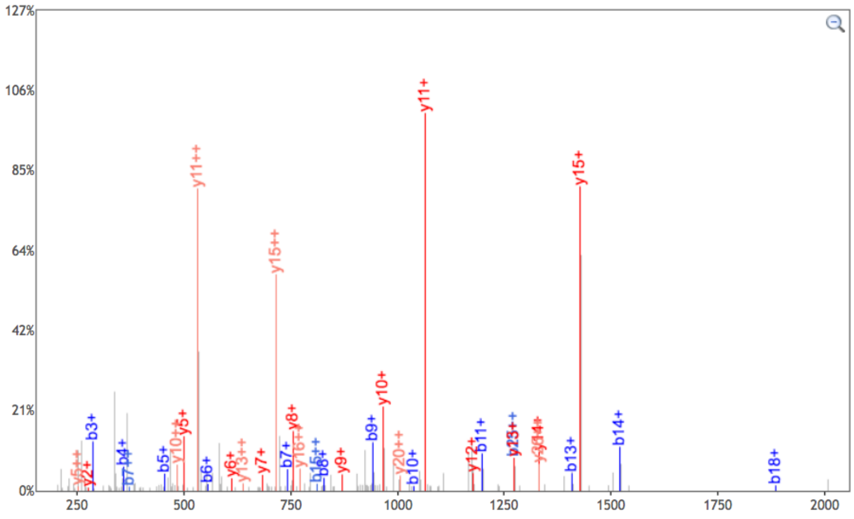

Click and drag in the plot to zoom X: ☑ Y: ☐ Zoom Out Print ☐Enable tooltip

b+	b2+	#	Seq	#	y+	y2+
116.0342	58.5207	1	D	29		
173.0557	87.0315	2	G	28	2833.5433	1417.2753
286.1397	143.5735	3	L	27	2776.5219	1388.7646
357.1769	179.0921	4	A	26	2663.4378	1332.2225
454.2296	227.6185	5	P	25	2592.4007	1296.7040
555.2773	278.1423	6	T	24	2495.3479	1248.1776
741.3566	371.1819	7	W	23	2394.3002	1197.6538
828.3886	414.6980	8	S	22	2208.2209	1104.6141
941.4727	471.2400	9	L	21	2121.1889	1061.0981
1038.5255	519.7664	10	P	20	2008.1048	1004.5561
1198.5561	599.7817	11	C	19	1911.0521	956.0297
1295.6089	648.3081	12	P	18	1751.0214	876.0143
1408.6929	704.8501	13	L	17	1653.9687	827.4880
1521.7770	761.3921	14	L	16	1540.8846	770.9459
1618.8298	809.9185	15	P	15	1427.8005	714.4039
1675.8512	838.4293	16	G	14	1330.7478	665.8775
1772.9040	886.9556	17	P	13	1273.7263	637.3668
1885.9881	943.4977	18	L	12	1176.6735	588.8404
1983.0408	992.0241	19	P	11	1063.5895	532.2984
2080.0936	1040.5504	20	P	10	966.5367	483.7720
2195.1205	1098.0639	21	D	9	869.4839	435.2456
2266.1576	1133.5825	22	A	8	754.4570	377.7321
2337.1948	1169.1010	23	A	7	683.4199	342.2136
2450.2788	1225.6431	24	L	6	612.3828	306.6950
2547.3316	1274.1694	25	P	5	499.2987	250.1530
2604.3531	1302.6802	26	G	4	402.2459	201.6266
2675.3902	1338.1987	27	A	3	345.2245	173.1159
2774.4586	1387.7329	28	V	2	274.1874	137.5973
		29	R	1	175.1190	88.0631

[Click] to move table

Static Modifications:
C: 57.02146

STSSSGRDPEMSLTQQQPSQEASVIQAGQPK, MH+ 3259.5495, m/z 1087.1880

File: HEK293-acid-highAGC-360minDE15-75um25cm-2, Scan: 19680, Precursor m/z: 1087.1965, Charge: 3

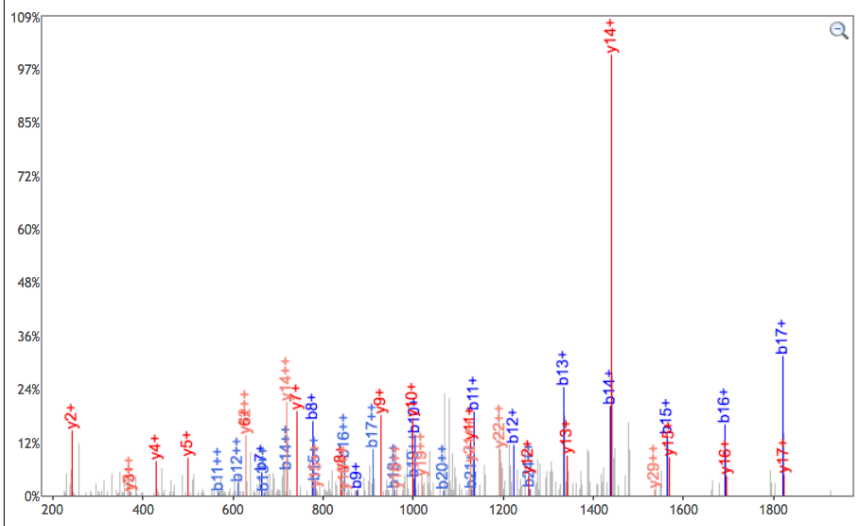

Click and drag in the plot to zoom X: Y: Zoom Out Print Enable tooltip

b+	b2+	#	Seq	#	y+	y2+
88.0393	44.5233	1	S	31		
189.0870	95.0471	2	T	30	3172.5175	1586.7624
276.1190	138.5631	3	S	29	3071.4698	1536.2386
363.1510	182.0792	4	S	28	2984.4378	1492.7225
450.1831	225.5952	5	S	27	2897.4058	1449.2065
507.2045	254.1059	6	G	26	2810.3737	1405.6905
663.3056	332.1565	7	R	25	2753.3523	1377.1798
778.3326	389.6699	8	D	24	2597.2512	1299.1292
875.3853	438.1963	9	P	23	2482.2242	1241.6158
1004.4279	502.7176	10	E	22	2385.1715	1193.0894
1135.4684	568.2379	11	M	21	2256.1289	1128.5681
1222.5005	611.7539	12	S	20	2125.0884	1063.0478
1335.5845	668.2959	13	L	19	2038.0564	1019.5318
1436.6322	718.8197	14	T	18	1924.9723	962.9898
1564.6908	782.8490	15	Q	17	1823.9246	912.4659
1692.7494	846.8783	16	Q	16	1695.8660	848.4367
1820.8079	910.9076	17	Q	15	1567.8075	784.4074
1917.8607	959.4340	18	P	14	1439.7489	720.3781
2004.8927	1002.9500	19	S	13	1342.6961	671.8517
2132.9513	1066.9793	20	Q	12	1255.6641	628.3357
2261.9939	1131.5006	21	E	11	1127.6055	564.3064
2333.0310	1167.0191	22	A	10	998.5629	499.7851
2420.0630	1210.5352	23	S	9	927.5258	464.2665
2519.1315	1260.0694	24	V	8	840.4938	420.7505
2632.2155	1316.6114	25	I	7	741.4254	371.2163
2760.2741	1380.6407	26	Q	6	628.3413	314.6743
2831.3112	1416.1592	27	A	5	500.2827	250.6450
2888.3327	1444.6700	28	G	4	429.2456	215.1264
3016.3912	1508.6993	29	Q	3	372.2241	186.6157
3113.4440	1557.2256	30	P	2	244.1656	122.5864
		31	K	1	147.1128	74.0600

[Click] to move table

PAWAPSPFAPGLSGPRPQAGPRPQAAPR, MH+ 2834.4961, m/z 945.5036

File: HEK293-acid-highAGC-360minDE15-75um25cm-2, Scan: 22458, Precursor m/z: 945.5069, Charge: 3

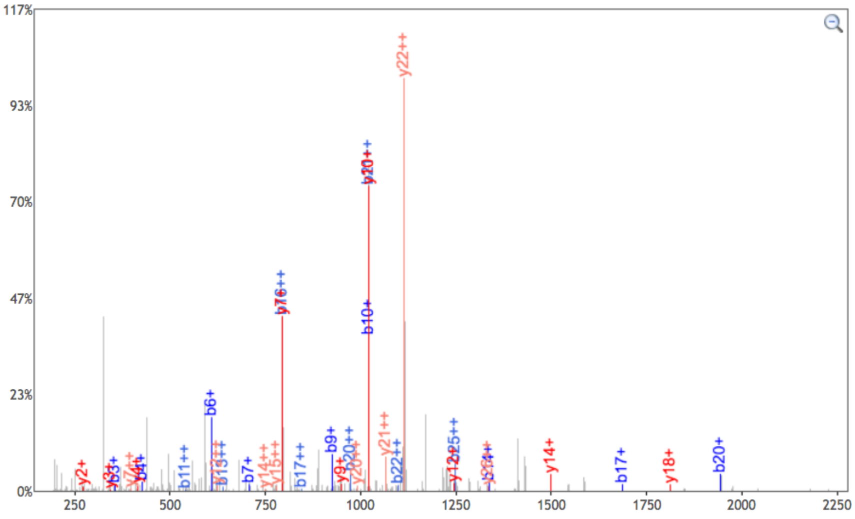

Click and drag in the plot to zoom X: Y: Zoom Out Print Enable tooltip

b+	b2+	#	Seq	#	y+	y2+
98.0600	49.5337	1	P	28		
169.0972	85.0522	2	A	27	2737.4434	1369.2253
355.1765	178.0919	3	W	26	2666.4062	1333.7068
426.2136	213.6104	4	A	25	2480.3269	1240.6671
523.2663	262.1368	5	P	24	2409.2898	1205.1485
610.2984	305.6528	6	S	23	2312.2371	1156.6222
707.3511	354.1792	7	P	22	2225.2050	1113.1062
854.4196	427.7134	8	F	21	2128.1523	1064.5798
925.4567	463.2320	9	A	20	1981.0839	991.0456
1022.5094	511.7584	10	P	19	1910.0467	955.5270
1079.5309	540.2691	11	G	18	1812.9940	907.0006
1192.6150	596.8111	12	L	17	1755.9725	878.4899
1279.6470	640.3271	13	S	16	1642.8884	821.9479
1336.6684	668.8379	14	G	15	1555.8564	778.4318
1433.7212	717.3642	15	P	14	1498.8350	749.9211
1589.8223	795.4148	16	R	13	1401.7822	701.3947
1686.8751	843.9412	17	P	12	1245.6811	623.3442
1814.9337	907.9705	18	Q	11	1148.6283	574.8178
1885.9708	943.4890	19	A	10	1020.5697	510.7885
1942.9922	971.9998	20	G	9	949.5326	475.2699
2040.0450	1020.5261	21	P	8	892.5112	446.7592
2196.1461	1098.5767	22	R	7	795.4584	398.2328
2293.1989	1147.1031	23	P	6	639.3573	320.1823
2421.2575	1211.1324	24	Q	5	542.3045	271.6559
2492.2946	1246.6509	25	A	4	414.2459	207.6266
2563.3317	1282.1695	26	A	3	343.2088	172.1081
2660.3844	1330.6959	27	P	2	272.1717	136.5895
		28	R	1	175.1190	88.0631

[Click] to move table

Static Modifications:
C: 57.02146

YGQISEK, MH+ 824.4149, m/z 412.7111

File: HEK293-acid-highAGC-360minDE15-75um25cm-2, Scan: 3854, Precursor m/z: 412.715, Charge: 2

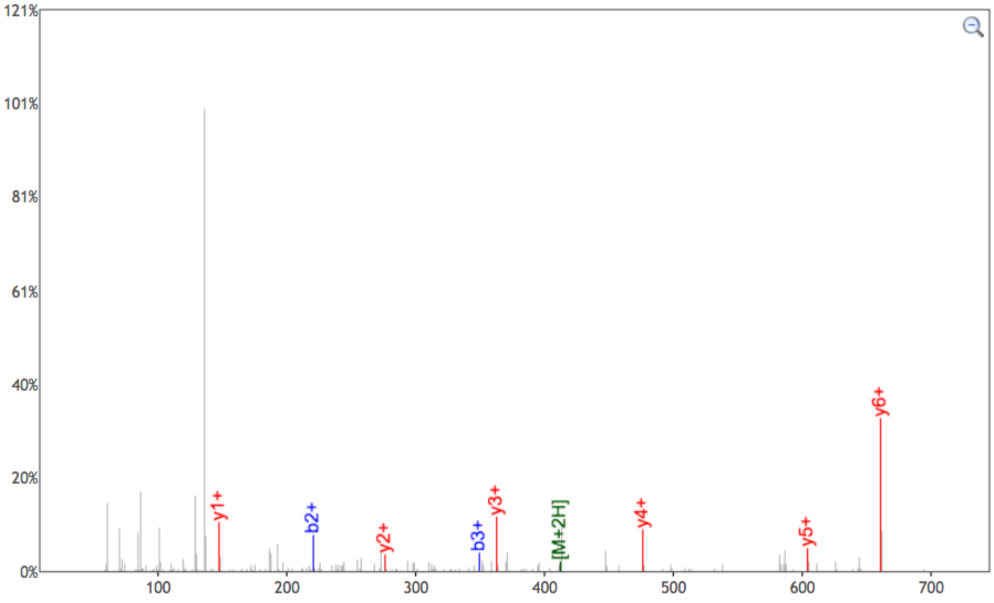

Click and drag in the plot to zoom X: Y: Zoom Out Print Enable tooltip

b+	#	Seq	#	y+
164.0706	1	Y	7	
221.0921	2	G	6	661.3515
349.1506	3	Q	5	604.3301
462.2347	4	I	4	476.2715
549.2667	5	S	3	363.1874
678.3093	6	E	2	276.1554
	7	K	1	147.1128

[Click] to move table

Static Modifications:
C: 57.02146

Fig F: Annotated MS2 spectra of detected peptides and corresponding MS2 spectra of the synthetic peptides. Spectra and their ion annotations are shown for all 45 detected peptides. Synthetic control spectra are shown for comparison.
